# Degradable Ureido‐Polycarbonate Block Copolymers with a Complex UCST Thermoresponse

**DOI:** 10.1002/marc.202500029

**Published:** 2025-03-22

**Authors:** Javier Martin‐Martin, Miriam Abad, Xabier Lopez de Pariza, Tiberio A. Ezquerra, Aurora Nogales, Haritz Sardon, Víctor Sebastián, Luis Oriol, Milagros Piñol

**Affiliations:** ^1^ Instituto de Nanociencia y Materiales de Aragón (INMA) CSIC‐Universidad de Zaragoza Zaragoza 50009 Spain; ^2^ Departamento de Química Orgánica Facultad de Ciencias Universidad de Zaragoza Pedro Cerbuna, 12 Zaragoza 50009 Spain; ^3^ POLYMAT and Department of Polymers and Advanced Materials: Physics Chemistry and Technology Faculty of Chemistry University of the Basque Country UPV/EHU Donostia‐San Sebastián 20018 Spain; ^4^ Instituto de Estructura de la Materia IEM‐CSIC C/Serrano, 121 Madrid 28006 Spain; ^5^ Department of Chemical Engineering and Environmental Technologies University of Zaragoza Zaragoza 50018 Spain; ^6^ Networking Research Center on Bioengineering Biomaterials and Nanomedicine (CIBER‐BBN) Madrid 28029 Spain; ^7^ Laboratorio de Microscopías Avanzadas Universidad de Zaragoza Zaragoza 50018 Spain

**Keywords:** amphiphilic block copolymers, degradable polymers, polymer self‐assembly, thermoresponsive polymers, UCST behavior

## Abstract

In this work, amphiphilic block copolymers (BCs) consisting of a hydrophilic poly(ethylene glycol) methyl ether (PEG) and a degradable polycarbonate block derived from 2,2‐bis(hydroxymethyl)propionic acid (bis‐MPA) with pendant ureido units, along with corresponding homopolycarbonates are described. Polymers are synthesized by combining ring opening polymerization (ROP) and thiol‐ene/yne functionalization to incorporate UCST‐promoting ureido groups. For homopolycarbonates, increasing the ureido groups density along the polymer chain facilitates the upper critical solution temperature (UCST)‐type thermoresponse in water. Because of their amphiphilic character, BCs form stable self‐assemblies either by direct dispersion in water, co‐solvent method or microfluidics. Upon heating, these self‐assemblies swell, and collapse due to extensive hydration of the polycarbonate block, rather than becoming solubilized. Thermoresponsiveness is analyzed in terms of the number of ureido groups in the polycarbonate for a given polycarbonate block length as well as the length of polycarbonate block. As a proof of concept, the potential of these self‐assemblies as thermoresponsive drug nanocarriers is evaluated, using curcumin as a hydrophobic model drug.

## Graphical abstract

Amphiphilic block copolymers with degradable ureido‐functionalized polycarbonate segments self‐assemble into spherical micelles in water, exhibiting an intriguing upper critical solution temperature (UCST) behavior. Upon heating, these micelles swell, collapse, and reorganize into highly hydrated condensates that mimic polymeric nanogels. Their potential as temperature‐responsive nanocarriers for hydrophobic drugs is demonstrated using curcumin as a drug model.

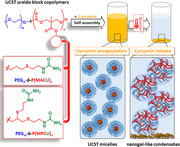

## 1 Introduction

Thermoresponsive polymers in water suffer changes in their physical and/or chemical properties, such as solubility, due to small variations of temperature. These polymers can be classified in two categories according to their temperature‐dependent solubility. The first category includes the most widespread type, which exhibits a lower critical solution temperature (LCST). They are soluble below a certain temperature, commonly known as cloud point (T_cp_), but insoluble above it. The second category encompasses the less common polymers exhibiting an upper critical solution temperature (UCST), which display the opposite behavior, being insoluble below T_cp_ and soluble above it.^[^ ^1^, ^2^ ^]^ In the last decade, great attention has been given to the development of new UCST polymers usually based on ionic polymers such as polyzwitterionic (polysulfobetaines)^[^ ^3^ ^]^ and polyelectrolytes.^[^ ^4^ ^]^ Non‐ionic polymers such as poly(*N*‐acryloylglycinamide),^[^ ^5^ ^]^ poly(acrylamide‐*co*‐acrylonitrile),^[^ ^6^ ^]^ and polymers bearing ureido groups^[^ ^7^ ^]^ have also been described, in which phase separation mainly results from reversible intra and inter‐chain hydrogen bonding interactions. Since the T_cp_ of non‐ionic polymers is less sensitive to changes in physiological media, they are more suitable and reliable for certain biomedical applications.

Temperature‐responsive amphiphilic block copolymers (BCs) exhibiting UCST behavior have been envisioned as a promising alternative for the development of smart drug nanocarriers. In particular, when consisting of a permanent hydrophilic block linked to a UCST block, they can spontaneously self‐assemble in water below the T_cp_, enabling drug encapsulation. Upon heating above the T_cp_, these drug‐loaded self‐assemblies may dissociate and dissolve, resulting in the release of the drug.^[^ ^8^, ^9^, ^10^ ^]^ However, some examples have been described where UCST self‐assemblies exhibit more complex thermoresponsive behavior, including changes in the morphology,^[^ ^11^, ^12^ ^]^ swelling^[^ ^13^ ^]^ or fusion^[^ ^14^, ^15^ ^]^ prior to their solubilization. In this way, Deane et al. reported on PEG‐*b*‐poly(hydroxybutyl acrylate) amphiphilic BCs, which underwent changes from micelles‐to‐worms‐to‐vesicles morphologies due to the progressive partial hydration of the hydrophobic block, though solubilization was not achieved.^[^ ^12^ ^]^ Still, Augé et al. described micelles from triblock copolymers with hydrophilic poly(*N*,*N*‐dimethylacrylamide) terminal blocks and a UCST poly(acrylamide‐*co*‐acrylonitrile) central block, which swell due to a hydration process and behave like nanogels before dissolving with increasing temperature.^[^ ^13^ ^]^ However, self‐assemblies may not swell due to water uptake but instead aggregate into clusters before solubilizing during the heating process. This is the case for micelles and worms self‐assembled from poly(*N*,*N*‐dimethylacrylamide)‐*b*‐poly(*N*‐cyanomethylacrylamide), which form fibers upon heating to 70 °C that precipitate before solubilizing at 90 °C.^[^ ^16^ ^]^ Also, Baddam et al. reported the coalescence of UCST micelles into large and loose particles prior to their solubilization at higher temperatures.^[^ ^14^ ^]^

Most reported UCST amphiphilic BCs rely on vinylic or (meth)acrylate polymers, which are non‐biodegradable being less suitable for biomedical applications. Therefore, access to degradable UCST‐based nanocarriers is highly desirable. To the best of our knowledge, only degradable BCs based on polypeptides,^[^ ^17^ ^]^ polyesters^[^ ^18^, ^19^ ^]^ and poly(phosphonate)s^[^ ^20^ ^]^ with UCST properties have been described to date. Aliphatic polycarbonates are polymers whose main chain degrades into small hydroxyl compounds and carbon dioxide due to the hydrolysis of the carbonate groups. These degradation products are featured by being non‐acid, in contrast to those generated in the degradation of aliphatic polyesters, resulting in a reduction of any inflammatory and immune response, when they are used in a biological medium.^[^ ^21^, ^22^ ^]^ Despite this advantage, only a UCST methacrylamide polymer with carbonate linkages in the side chain has been described up to now. However, after the hydrolysis of carbonate groups, the polymethacrylamide scaffold remained intact due to its lack of degradability.^[^ ^23^ ^]^ Therefore, UCST polycarbonates where the main chain is degradable and disintegrated into small molecules have not been described so far.

In this context, the main aim of this work was to develop non‐ionic UCST degradable polycarbonates and temperature responsive BC nanoparticles comprising the polycarbonate block and PEG as hydrophilic block. Therefore, the synthesis of amphiphilic BCs featuring a PEG block (degree of polymerization, DP, approx. 45 and number molar mass, M_n_, approx. 2000 g mol^−1^) and a polycarbonate block was targeted. PEG was selected due to its solubility in water below 100 °C and its common use in stabilizing amphiphilic self‐assembled structures in water. The thermosensitive block was a neutral aliphatic polycarbonate with ureido side groups whose UCST behavior in water stems from intra‐ and intermolecular hydrogen bonding.

**Figure 1:**
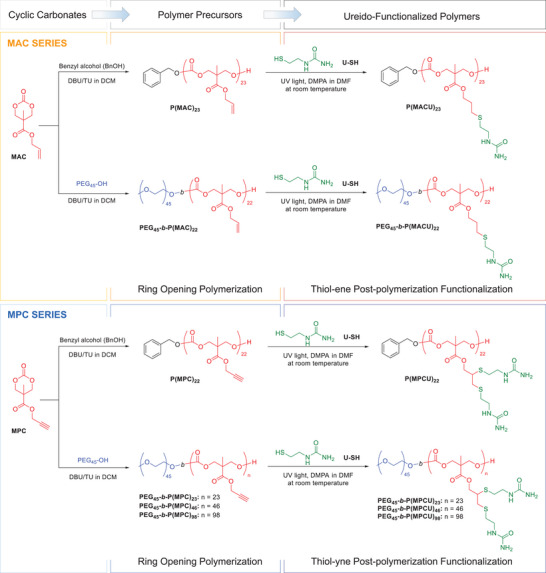
Synthesis of homopolycarbonates and amphiphilic BCs.

In polymers, thermoresponse requires functional macromolecules with precisely defined composition, dispersity, molecular mass, and terminal groups to guarantee reliable self‐assembling properties at the nanoscale but also changes in solubility within well‐defined intervals. Thus, a synthetic strategy combining a controlled ring opening polymerization (ROP) and post‐polymerization functionalization by thiol‐ene/yne chemistry was devised. Two polymer series were synthesized using 2,2‐bis(hydroxymethyl)propionic acid (bis‐MPA) derived cyclic carbonate monomers 5‐methyl‐5‐allyloxycarbonyl‐1,3‐dioxan‐2‐one (MAC) and 5‐methyl‐5‐propargyloxycarbonyl‐1,3‐dioxan‐2‐one (MPC) (**Figure** **1**). This approach has proven effective for the incorporation of either one or two UCST‐promoter ureido groups per repeating unit within the polycarbonate block, offering a versatile method to modulate the thermoresponsiveness.

## 2 Results and Discussion

### 2.1 Synthesis and Characterization of Ureido‐Functionalized Polymers

First, homopolycarbonates and the corresponding amphiphilic BC precursors with either allyl or propargyl side groups were obtained by ROP of MAC and MPC,^[^ ^24^ ^]^ using either benzyl alcohol or PEG with a M_n_ ≈ 2000 g mol^−1^ (average DP = 45) as initiators (Figure 1). ROP was performed in dichloromethane (DCM) at 35 °C using the organocatalytic system of 1,8‐diazabicyclo(5.4.0)undec‐7‐ene (DBU) and 1‐(3,5‐bis(trifluoromethyl)‐phenyl)‐3‐cyclohexylthiourea (TU), which has previously described to provide good control and fast polymerization rates for these specific cyclic monomers.^[^ ^25^, ^26^ ^]^ The degree of polymerization (DP^theo^) of homopolycarbonates and polycarbonate blocks was initially set at 25. Besides, DP^theo^ of 55, and 105 were also targeted for PEG_45_‐*b*‐P(MPC)_n_. The ROP was stopped after 2–3 h when reaching approx. 80% conversion in order to avoid side‐reactions that can take place at higher conversions.^[^ ^26^ ^]^ Due to the challenge in extending the polycarbonate chain length beyond DP ≈ 25 using Schlenk techniques, the ROP of BCs with polycarbonate blocks of DP ≈ 50 and 90 was conducted in a glovebox, where superior inert and dry conditions could be maintained. The control of the polymerization and the absence of side‐reactions was verified by ^1^H NMR, and the monomodal mass distributions and low dispersities found by size exclusion chromatography (SEC) analysis, and MALDI‐TOF mass spectrometry. The DP and M_n_ were determined by end‐group analysis using ^1^H NMR. Both DP^NMR^ and M_n_^NMR^ were consistent with the values obtained from SEC, considering that poly(methyl methacrylate) (PMMA) was used as the calibration standard. Relevant information is gathered in **Table** **1** (Figures S16–S20, Supporting Information).

**Table 1:** Composition and molar masses of the precursor polymers with ally or propargyl side groups prepared by ROP.

Polymer	DP^theo^ ^a)^	DP^NMR^ ^b)^	M_n_^NMR^ [g mol^−1^] ^c)^	M_n_^SEC^ [g mol^−1^] ^d)^	*Đ* ^d)^
P(MAC)_23_	25	23	4712	7790	1.12
P(MPC)_22_	25	22	4468	7504	1.12
PEG_45_‐*b*‐P(MAC)_22_	25	22	6418	10 582	1.12
PEG_45_‐*b*‐P(MPC)_23_	25	23	6572	10 900	1.14
PEG_45_‐*b*‐P(MPC)_46_	55	46	11 130	14 362	1.10
PEG_45_‐*b*‐P(MPC)_98_	105	98	21 435	23 046	1.11

^a)^ Theoretical DP of the polycarbonate determined from the [monomer]₀/[I]₀ ratio at 100% conversion;

^b)^ DP determined by ^1^H NMR;

^c)^ Number molar mass calculated from DP^NMR^. For BCs, M_n_^NMR^ was calculated as the sum of M_n_ of PEG block (assuming DP = 45 for PEG_45_‐OH) and M_n_^NMR^ of polycarbonate block;

^d)^ M_n_ and dispersity (*Đ*) determined by SEC in THF (1 mL min^−1^) using PMMA calibration standards and an evaporative light scattering detector.

Ureido polymers were synthesized by UV initiated thiol‐ene or thiol‐yne reactions using 1‐(2‐mercaptoethyl)urea (U‐SH) (Figure 1). The reaction was carried out in *N,N*‐dimethylformamide (DMF) using the photoinitiator, 2,2‐dimethoxy‐2‐phenyl acetophenone (DMPA) and an excess of thiol under exposure to 365 nm light. The reaction was monitored by ^1^H NMR until completion, within the limits of the technique. The functionalization of polymers from the MAC series was monitored by the disappearance of signals corresponding to vinyl protons and appearance of new methylenic proton signals. Accordingly, for MPC series, the resonance corresponding to methylenic protons of propargyl group shifted upfield. Additionally, the functionalization of MPC series was assessed by FTIR spectroscopy following the disappearance of the terminal alkyne vibration band (C≡CH*^st^* at 2123 cm^−1^) and the emergence of new bands corresponding to the ureido group (Figures S21 and S22, Supporting Information). Full experimental details are given in Supporting Information

The thermal properties of ureido polymers in bulk were evaluated by thermogravimetric analysis (TGA) and differential scanning calorimetry (DSC) (Table S2 and Figure S23, Supporting Information). Ureido polymers exhibited good thermal stability under nitrogen atmosphere with decomposition temperatures leading to significant mass loss at approx. 190 °C. Thermal transitions were studied by DSC. Once removing the previous thermal history, both homopolycarbonates were identified as amorphous polymers. P(MACU)_23_ showed a glass transition temperature (*T_g_*) at 17 °C, whereas P(MPCU)_22_ exhibited a higher *T_g_* ≈40 °C. Regarding the BCs, PEG_45_‐*b*‐P(MACU)_22_ was obtained as a viscous oil with a sub‐ambient glass transition at ‒8 °C and traces of crystallinity corresponding to PEG segment were not detected. This single recorded *T_g_* was significantly lower than that of P(MACU)_23_ homopolycarbonate, suggesting (at least partial) miscibility of the blocks in the bulk. Polymers of the MPC series were also found to be amorphous with a single *T_g_*. For PEG_45_‐*b*‐P(MPCU)_23_, *T_g_* was 21 °C, significantly lower than that of P(MPCU)_22_, indicating again miscibility of the blocks. As the length of the polycarbonate increased, from PEG_45_‐*b*‐P(MPCU)_46_ and PEG_45_‐*b*‐P(MPCU)_98_, a similar behavior was observed with the *T_g_* increasing to 33 and 40 °C, respectively, according to the expected behavior for moderate DPs.

### 2.2 Self‐Assembly Properties of BCs

**Figure 2:**
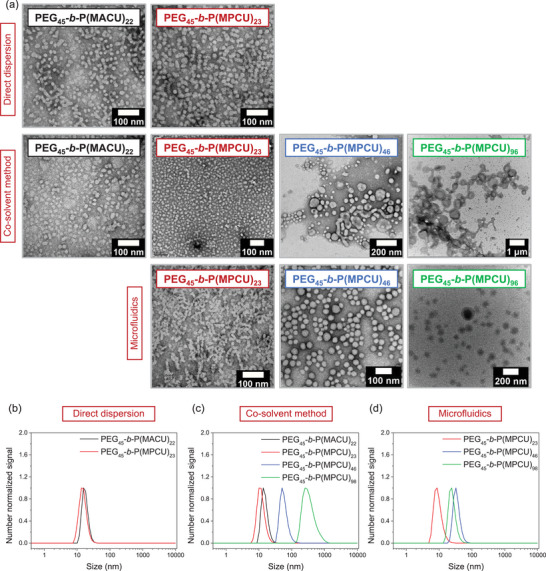
a) TEM images (samples stained with uranyl acetate) and DLS number size distributions at 25 °C of self‐assemblies prepared by b) direct dispersion, c) co‐solvent method and d) microfluidics. Samples were prepared at 1.0 mg mL^−1^ polymer concentration in water.

Self‐assembly of amphiphilic BCs in water involves both thermodynamic and kinetic factors, meaning that the resulting morphology can vary depending on the preparation conditions. Therefore, methods such as direct dispersion, co‐solvent approach and microfluidics, were employed to prepare self‐assemblies in water. The self‐assembled structures were analyzed by combining transmission electron microscopy (TEM) to examine the morphology, and dynamic light scattering (DLS), for determining the particle size and distribution (**Figure** **2**).

PEG_45_‐*b*‐P(MACU)_22_ and PEG_45_‐*b*‐P(MPCU)_23_ were first self‐assembled by direct dispersion in Milli‐Q water. This technique, well‐suited for BCs with moderate hydrophobicity, stands out for its simplicity and efficiency as polymer is dispersed directly in water, avoiding organic solvents that can be detrimental in biomedical applications.^[^ ^27^ ^]^ Therefore, self‐assemblies dispersions were prepared by suspending the polymer in Milli‐Q water and stirring it in an ultrasonic bath for 10 min at room temperature (rt). Small micelles were observed by TEM having diameters of 12 ± 4 and 15 ± 4 nm for PEG_45_‐*b*‐P(MACU)_22_ and PEG_45_‐*b*‐P(MPCU)_23_, respectively (Figure 2a). By DLS analysis, average hydrodynamic diameter (*D_h_*) of approx. 15 nm were determined on the number size distribution (Figure 2b). Intensity size distribution plots also revealed a few amount of micellar aggregates (Figure S24a, Supporting Information).^[^ ^27^, ^28^, ^29^ ^]^

Self‐assembly of BCs having longer polycarbonate chains, PEG_45_‐*b*‐P(MPCU)_46_ and PEG_45_‐*b*‐P(MPCU)_98_, was not possible by direct dispersion in water, likely due to their higher hydrophobic‐to‐hydrophilic ratio. Therefore, nanoprecipitation using co‐solvent technique was employed using dimethylsulfoxide (DMSO) as good solvent for both blocks, and water as selective solvent (Figure S25a, Supporting Information). This methodology usually results in better control over the self‐assembly process and morphologies compared to direct dispersion in water.^[^ ^30^ ^]^ The critical aggregation concentration (CAC) of the BCs was determined by fluorescence spectroscopy employing Nile Red as a polarity sensitive probe to estimate the thermodynamic stability of the self‐assemblies (Figure S25b, Supporting Information). Typically, the CAC decreases if the hydrophobic‐to‐hydrophilic block length ratio increases. Longer hydrophobic polymer blocks confer greater thermodynamic stability because enhanced hydrophobic interactions favor cohesion and the formation of micellar cores at lower polymer concentrations.^[^ ^31^ ^]^ Therefore, as expected, CAC of PEG_45_‐*b*‐P(MPCU)_n_ self‐assemblies decreased while increasing the length (n) of the polycarbonate block from 54 µg mL^−1^ for *n* = 23, to 45 µg mL^−1^ for *n* = 46 and 42 µg mL^−1^ for *n* = 98. When PEG_45_‐*b*‐P(MACU)_22_ was compared with PEG_45_‐*b*‐P(MPCU)_23_, both having similar polycarbonate length, the CAC of PEG_45_‐*b*‐P(MACU)_22_, with a lower ureido content, was 37 µg mL^−1^. This trend might be somehow unexpected because the incorporation of hydrogen bonding in a core‐forming hydrophobic block has typically been described to improve micelle stabilization. Thus, the observed increase in CAC should be more likely due to differences in hydrophobicity. The calculated log *P* parameter was used to estimate the hydrophobicity of the repeating units.^[^ ^7^, ^32^ ^]^ Log *P* calculated for repeating unit of MACU (+0.23) and MPCU (‒0.63) confirmed that the incorporation of two ureido groups significantly decreases hydrophobicity, and this reduction can explain the decrease in thermodynamic stability of micelles composed of MPC polymers.

PEG_45_‐*b*‐P(MACU)_22_ and PEG_45_‐*b*‐P(MPCU)_23_ dispersions were transparent and comprised stable spherical micelles of similar sizes to those prepared directly in water (Figure 2a,c; Figure S24b, Supporting Information). According to TEM images, spherical micelles with average diameters of approx. 14 nm were observed and exhibited similar *D_h_* values by DLS (15 and 11 nm). By increasing the length of the hydrophobic polycarbonate block in PEG_45_‐*b*‐P(MPCU)_n_ series, the visual appearance of the dispersions changed from transparent to cloudy. TEM analysis revealed that this change was due to a transition in self‐assembly morphology, involving a progressive evolution from micelles‐to‐worms‐to‐vesicles (Figure 2a). Therefore, TEM characterization of PEG_45_‐*b*‐P(MPCU)_46_ revealed the coexistence of larger spheres with an average diameter of 40 ± 16 nm alongside a small population of cylindrical micelles (cross‐sectional diameter of 34 ± 15 nm). For PEG_45_‐*b*‐P(MPCU)_98_ a predominant population of deflated vesicles was observed, some of which appeared to be interconnected by worms. Additionally, DLS analysis indicated a significant increase in size, although it has to be noted that the apparent *D_h_* might not be representative for non‐spherical morphologies (Figure 2c). Furthermore, after dialysis of PEG_45_‐*b*‐P(MPCU)_98_ dispersion, a small portion of precipitate appeared due to a possible sedimentation of the larger polymeric aggregates.

Self‐assembly of PEG_45_‐*b*‐P(MPCU)_n_ polymers was also conducted by microfluidics. This technology offers notable advantages over conventional batch methods such as high reproducibility and scalability of the production of self‐assemblies. Polymer nanostructures with well‐defined characteristics such as controlled size, narrow size distribution, and controlled morphology can be fabricated by adjusting different parameters such as flow rate, aqueous/organic solution phase ratios, polymer concentration or temperature.^[^ ^33^, ^34^ ^]^ In this study, self‐assembly via microfluidics was conducted by mixing a 5.0 mg mL^−1^ polymeric solution in DMSO with Milli‐Q water in a commercial slit interdigital microstructured mixer.^[^ ^33^ ^]^ Both solutions were fed in continuous and non‐pulsed flow into the micromixer using two syringe pumps. The self‐assemblies were generated using an aqueous/organic phase ratio of 4:1 and a residence time of 48 ms (10 mL min^−1^ flow rate). This specific aqueous/organic solution ratio was chosen because self‐assembly was previously observed with this ratio using the co‐solvent method. The residence time was determined to be appropriate based on previous research.^[^ ^35^ ^]^ When comparing PEG_45_‐*b*‐P(MPCU)_23_ dispersions, no significant differences either in morphology or size were observed between the microfluidic and co‐solvent methods (Figure 2). In contrast, the dispersions of PEG_45_‐*b*‐P(MPCU)_46_ and PEG_45_‐*b*‐P(MPCU)_98_ were turbid, but to a lesser extent than those obtained by the co‐solvent method. Furthermore, no precipitate formation was observed for PEG_45_‐*b*‐P(MPCU)_98_ self‐assemblies. Unlike the self‐assemblies produced by the co‐solvent method, only homogeneous stable spherical nanostructures with a diameter of 28 ± 11 nm for PEG_45_‐*b*‐P(MPCU)_46_ and 65 ± 25 nm for PEG_45_‐*b*‐P(MPCU)_98_ were detected in TEM images (Figure 2a). Using this processing methodology, the transition to non‐spherical morphologies when increases the length of the polycarbonate block was not observed. The *D_h_* of PEG_45_‐*b*‐P(MPCU)_46_ and PEG_45_‐*b*‐P(MPCU)_98_ measured from the DLS number size distributions were 34 ± 8 and 26 ± 7 nm, respectively (Figure 2d; Figure S24c, Supporting Information).

### 2.3 Thermal Behavior of P(MACU)_23_ and P(MPCU)_22_ Homopolycarbonates in Water

**Figure 3:**
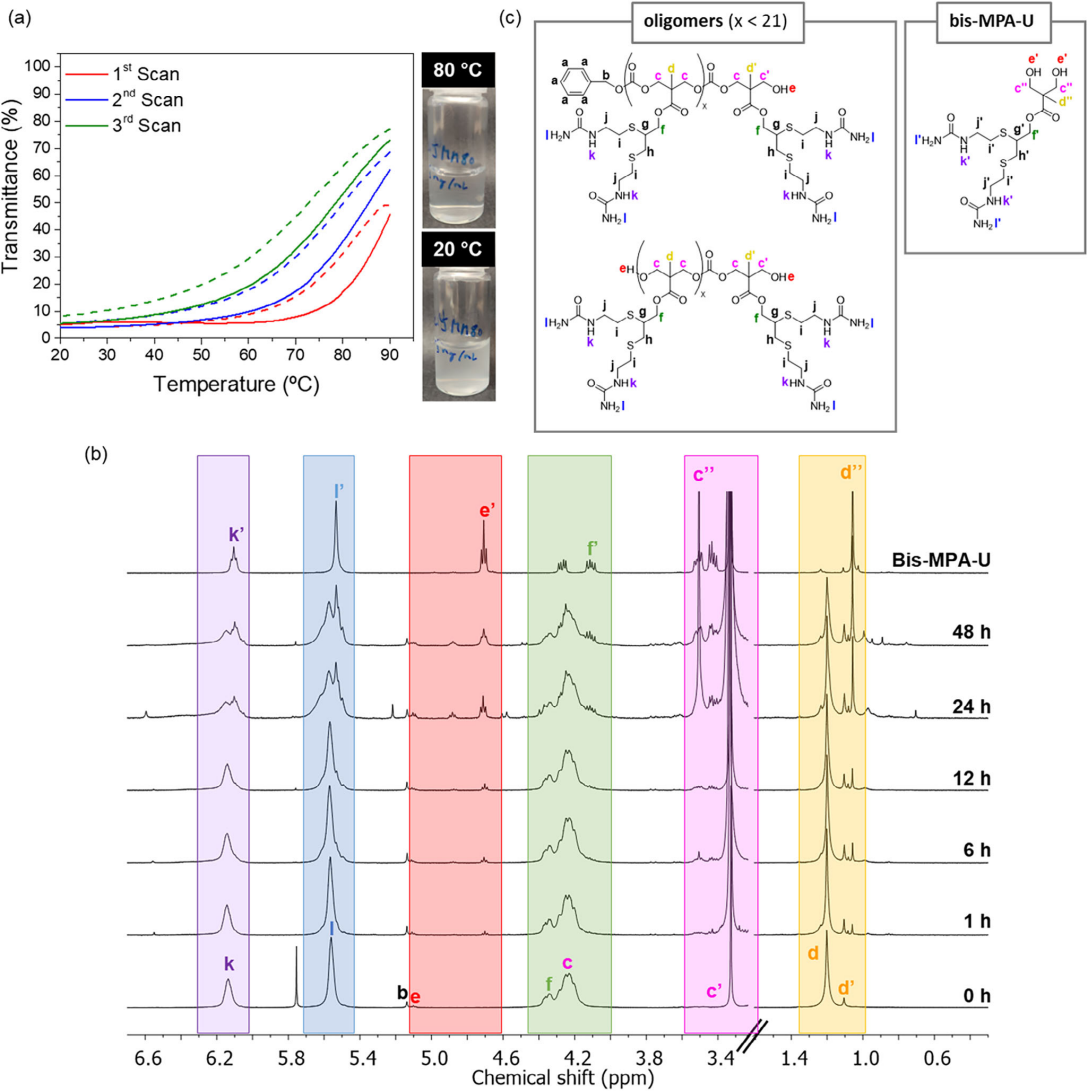
a) Temperature‐dependent transmittance curves of P(MPCU)_22_ in water (0.3 mg mL^−1^) upon repeated heating (solid line) and cooling (dashed line) at 1.0 °C min^−1^. Inserted photographs of P(MPCU)_22_ at 20 and 80 °C. b) ^1^H NMR (400 MHz, DMSO‐d_6_) spectra of freeze‐dried P(MPCU)_22_ samples after heating at 80 °C in Milli‐Q water for different time periods (^1^H NMR of bis‐MPA‐U is included as reference). c) Main expected degradation products generated by carbonate hydrolysis of P(MPCU)_22._

Although homopolycarbonates do not form self‐assemblies, polymer dispersions were prepared by dissolving the polymer in DMSO and gradual addition of water, followed by dialysis against Milli‐Q water. This alternative was preferred over direct dispersion in hot water because it allows for homogeneous dispersions of fine particles while avoiding prolonged exposure to high temperatures, which could lead to polymer degradation (see below). The thermoresponse of P(MACU)_23_ and P(MPCU)_22_ dispersions was evaluated by turbidimetry. Transmittance curves at 650 nm were recorded upon three consecutive heating/cooling scans from 20 to 90 °C at a scanning rate of 1.0 °C min^−1^. Visual inspection and transmission plots of the P(MACU)_23_ sample, registered at different polymer concentrations from 0.1 to 1.0 mg mL^−1^, indicated that this polymer did not dissolve when the dispersion was heated (Figures S26a–c, Supporting Information). Conversely, a preliminary visual assessment of the P(MPCU)_22_ suspension in water at 0.3 mg mL^−1^ indicated that the polymer solubility increased upon heating. Upon cooling to rt, the dispersion became cloudier. Transmittance curves recorded at 0.3 mg mL^−1^ were consistent with visual inspection (**Figure** **3**a). During heating, an increase in the optical transmittance above 60 °C was associated with progressive polymer solubilization, which is indicative of UCST behavior. However, the polymer was not fully solubilized at 90 °C. This behavior was reversible upon cooling, showing some hysteresis, which is common when the responsiveness of UCST polymers is driven by hydrogen bonds within polymer chains. Transmittance changes as a function of temperature were also measured at different concentrations, i.e., 0.1 and 1.0 mg mL^−1^ (Figures S26d,e, Supporting Information). Higher temperatures were needed to solubilize the polymer as the concentration increased, characteristic of typical UCST behavior.^[^ ^36^, ^37^ ^]^ Differences in thermal response between P(MPCU)_22_ and P(MACU)_23_ may be attributed to a reduction in the hydrophobicity of the polymer when the number of ureido groups per repeating unit increases, as deduced from the comparisons of log *P* (see above). The decrease of hydrophobicity is expected to facilitate the solubilization of the polymer upon heating.

When the reproducibility of the P(MPCU)_22_ thermoresponse was examined along three subsequent heating/cooling cycles, a notable decrease in temperature transition was observed with a simultaneous increase in transmittance at the maximum registered temperature (90 °C) (Figure 3a; Figure S26d,e, Supporting Information). This performance was likely attributed to the hydrolysis of carbonate groups,^[^ ^23^ ^]^ which might have resulted in the polycarbonate degradation, generating shorter chains of improved solubility in water and thus lowering the T_cp_. Therefore, ^1^H NMR was employed to evaluate the stability of the polycarbonate in water (Figure 3b). P(MPCU)_22_ was incubated at 80 °C in water (1.0 mg mL^−1^) for different time intervals, after which the samples were freeze‐dried and the resulting solids dissolved in DMSO‐d_6_ for ^1^H NMR study. Hydrolytic cleavage of the carbonate groups of the polymeric main chain can generate different degradation compounds such as monohydroxy‐ or dihydroxy‐terminated oligomers and the bis‐MPA‐U 1,3‐diol (Figure 3c) as major products. The bis‐MPA‐U diol was synthesized as a reference to identify the ^1^H NMR signals. Upon heating for more than 1 h, the ^1^H NMR polymer signals became sharper and better resolved as expected if the macromolecule is hydrolyzed into smaller fragments with increased mobility. This fragmentation was supported by the increase of the relative integration of hydroxyl and methyl signals corresponding to the terminal units of polycarbonate main chain at 1.11 and 5.11 ppm (labeled as *d’* and *e*). Besides, new signals corresponding to the bis‐MPA‐U at 4.71 ppm (labelled as *e’*), 3.43 ppm (labeled as *c’’*) and 1.06 ppm (labelled as *d’’*) appeared and increased over time as result of progressive degradation of carbonate groups connecting repeating units.

### 2.4 Thermal Behavior of BC Self‐Assemblies in Water

**Figure 4:**
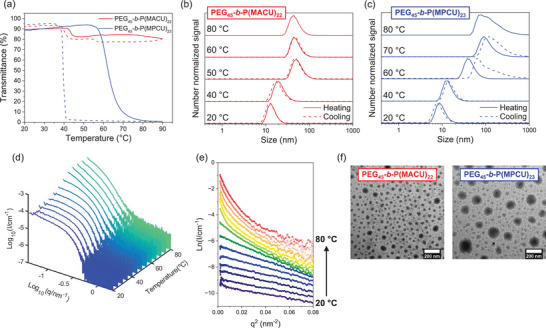
Temperature‐dependent a) transmittance (650 nm) curves and, b,c) DLS number size distributions of PEG_45_‐*b*‐P(MACU)_22_ and PEG_45_‐*b*‐P(MPCU)_23_ at 5.0 mg mL^−1^ concentration in water upon heating (solid line) and cooling (dashed line). d) SAXS patterns and e) Guinier plot for PEG_45_‐*b*‐P(MPCU)_23_ at 1.0 mg mL^‒1^ polymer concentration recorded on heating between 20 and 80 °C. f) TEM images of PEG_45_‐*b*‐P(MACU)_22_ and PEG_45_‐*b*‐P(MPCU)_23_ dispersions at 70 °C (stained with phosphotungstic acid). All samples were prepared by direct dispersion in water.

Despite the high UCST temperature of the homopolycarbonates, amphiphilic BC self‐assemblies were investigated as the increase of hydrophilicity produced by the PEG block is expected to cause a decrease in the T_cp_ of the polycarbonate block.^[^ ^38^, ^39^, ^40^ ^]^ The thermoresponse of PEG_45_‐*b*‐P(MACU)_22_ and PEG_45_‐*b*‐P(MPCU)_23_ micelles was evaluated using samples prepared by direct dispersion in Milli‐Q water at a 5.0 mg mL^−1^ polymer concentration by turbidimetry and DLS. At rt, both PEG_45_‐*b*‐P(MACU)_22_ and PEG_45_‐*b*‐P(MPCU)_23_ samples were transparent to the naked eye. The high initial transmittance of PEG_45_‐*b*‐P(MACU)_22_ dispersion slightly decreased at 45 °C upon heating, correlating with growth in size from 14 nm at 20 °C to approx. 65 nm above 45 °C, as determined by DLS (**Figure** **4**a,b). On cooling, the thermoresponsive behavior was reversible (only a small hysteresis was observed). Besides, a second small jump was observed at ≈55 °C on the transmittance curve. On consecutive heating‐cooling cycles a similar behavior was observed although transition temperatures were slightly downshifted probably due to partial hydrolysis (Figure S27a, Supporting Information).

PEG_45_‐*b*‐P(MPCU)_23_ dispersion exhibited a more significant drop in transmittance upon heating, at a higher temperature, ≈60 °C (Figure 4a). This decline in transmittance correlated with a significant increase in the apparent diameter of the self‐assemblies, which progressively grew to approx. 145 nm at 80 °C, according to DLS number signal, accompanied by a remarkable broad size distribution (Figure 4c). On cooling, changes were reversible with a significant hysteresis of approx. 20 °C, which could be attributed to the higher number of hydrogen bonds resulting from the greater amount of ureido groups when compared to PEG_45_‐*b*‐P(MACU)_22_. The transmittance curves were reproducible upon consecutive heating/cooling scans considering the possibility of partial hydrolysis of the polycarbonate chain (Figure S27b, Supporting information). Interestingly, upon cooling from 80 °C, the average number size estimated from the broad DLS signals initially increased but then progressively decreased, ultimately returning to the initial average number size and dispersity values when samples were cooled below the transition temperature, ≈40 °C (Figure 4c; Figure S27c, Supporting Information).

The influence of polymer concentration in the thermoresponse was also evaluated at 1.0 and 10.0 mg mL^−1^ (Figure S28, Supporting Information). At 10.0 mg mL^−1^, PEG_45_‐*b*‐P(MACU)_22_ showed similar temperature‐induced changes to 5.0 mg mL^−1^, with the main difference being a more pronounced variation in transmittance. When reducing the polymer concentration to 1.0 mg mL^−1^, transmittance changes were hardly detectable. Nevertheless, a clear increase in size was observed by DLS, reaching comparable values across different concentrations, although the transition was broader, occurring between 40 and 50 °C. Again, for PEG_45_‐*b*‐P(MPCU)_23_, the turbidity and size evolution at 10.0 mg mL^−1^ appeared similar to that described for 5.0 mg mL^−1^. However, for the lower polymer concentration (1.0 mg mL^−1^), temperature‐dependent transmittance curves showed some evident differences. Upon heating, only a small change in transmittance was observed. Thereby, a slight decrease in transmittance at ≈65 °C was registered (even with a partial recovery of transmittance). However, the temperature evolution of average number *D_h_* plot showed a progressive increase in the micellar size from 45 to 80 °C. On cooling, changes were more dramatic. In the temperature range from 80 to approx. 45 °C, turbidity gradually increased along with an increase in *D_h_*. Upon further cooling, in the temperature range from 45 to 20 °C, turbidity and micellar size sharply decreased, and the initial values were almost fully recovered at 35 °C.

The thermal behavior was also investigated in PBS as a relevant physiological medium (pH 7.4) at 5.0 mg mL^−1^ polymer concentration. Upon heating, a decrease in the transmittance of PEG_45_‐*b*‐P(MACU)_22_ and PEG_45_‐*b*‐P(MPCU)_23_ dispersions were registered at 35 and 15 °C, respectively (Figure S29 and Video S1, Supporting Information). The thermoresponse was reversible on cooling and reproducible during several heating/cooling cycles. Thus, the phase transition in PBS occurred at lower temperatures compared to water, with this effect being significantly more pronounced in the case of the PEG_45_‐*b*‐P(MPCU)_23_ copolymer.

The thermal response of PEG_45_‐*b*‐P(MPCU)_23_ in water was investigated in more detail by small‐angle X‐ray scattering (SAXS) analysis at variable temperatures between 20 and 80 °C, both upon heating and subsequent cooling for a dispersion with a polymer concentration of 1.0 mg mL^−1^ (Figure 4d,e; Figure S30, Supporting Information). At 25 °C, the scattering curve was well‐fitted to globular nanostructures of ≈18 nm in diameter, as estimated using the Guinier approach for globular objects, which was consistent with DLS analysis and TEM observations. Between 40 and 50 °C, the SAXS analysis indicated a slight increase in the sphere diameter, reaching up to 28 nm. However, above 55 °C, the scattering curves no longer fit well to a simple sphere distribution model. On cooling, the thermal behavior was reversible and the SAXS curves could be fitted again to globular self‐assemblies below 55 °C. Changes on PEG_45_‐*b*‐P(MPCU)_23_ at 70 °C were visualized by TEM revealing a transformation from the initial micelles to larger spherical aggregates of ≈100–150 nm in size (Figure 4f), which correlated well with average *D_h_* values determined by DLS. Therefore, SAXS experiments conducted above 55 °C would be more consistent with an aggregation process rather than a simple swelling‐driven growth of the spherical micelles. The large size of the aggregates likely accounts for the loss of adjustment, as it exceeds the resolution limits imposed by the restricted q range. TEM images collected for PEG_45_‐*b*‐P(MACU)_22_ at 70 °C reveal a similar situation with spherical aggregates of approx. 30–40 nm (Figure 4f).

**Figure 5:**
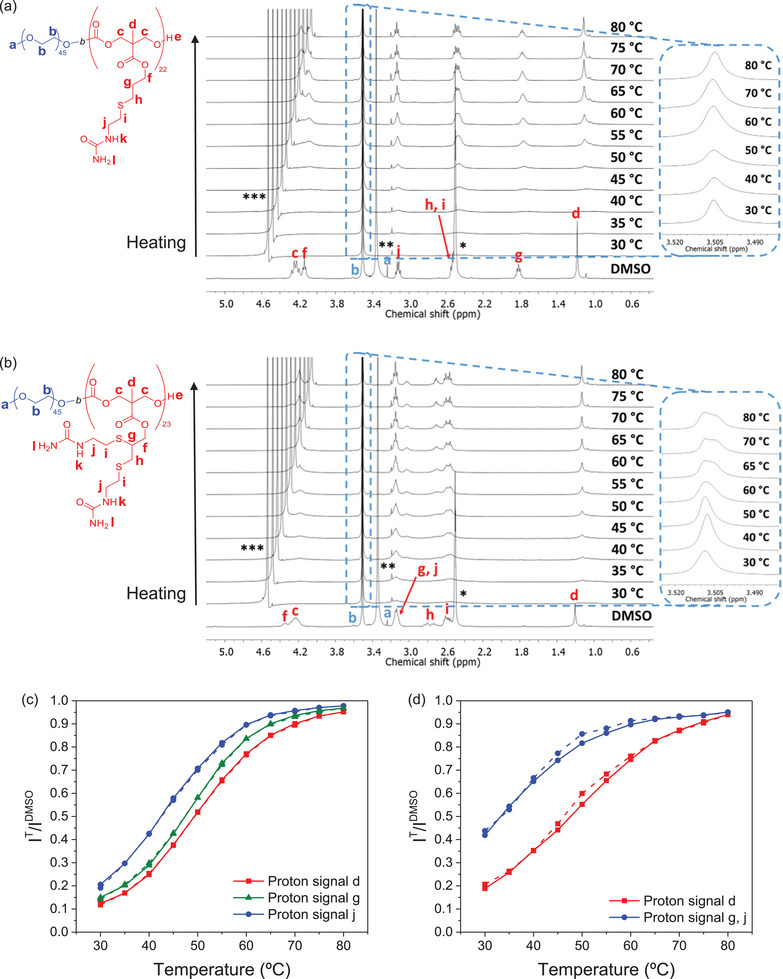
^1^H NMR (400 MHz, DMSO‐d_6_) reference spectrum at 25 °C and temperature‐dependent ^1^H NMR (400 MHz, D_2_O) spectra of a) PEG_45_‐*b*‐P(MACU)_22_ and b) PEG_45_‐*b*‐P(MPCU)_23_ at 5.0 mg mL^−1^ concentration registered upon heating, with temperature intervals of 5 °C and equilibrium time of 5 min between each measurement. * Resonances corresponding to DMSO. ** Resonances corresponding to H_2_O. *** Resonances corresponding to HDO. Temperature‐dependent evolution of the NMR normalized intensities in D_2_O relative to normalized intensities in DMSO‐d_6_ of c) PEG_45_‐*b*‐P(MACU)_22_ and d) PEG_45_‐*b*‐P(MPCU)_23_ upon heating (solid line) and cooling (dashed line).

From the results discussed above, neither PEG_45_‐*b*‐P(MACU)_22_ nor PEG_45_‐*b*‐P(MPCU)_23_ micelles dissociate and dissolve into individual polymer chains when heated up to 80 °C.^[^ ^7^, ^41^ ^]^ In order to gain deeper information about the molecular mechanism that causes the size growth of the spherical polymer assemblies, variable temperature ^1^H NMR experiments were conducted in D_2_O at 5.0 mg mL^−1^ polymer concentration. The dispersion was heated and cooled from 30 to 80 °C, with ^1^H NMR spectra recorded every 5 °C after equilibrating for 5 min (**Figure** **5**). The resonances were assigned to the different protons using as reference the ^1^H NMR spectrum recorded in DMSO‐d_6_ to ensure complete dissolution of both blocks at rt. In D_2_O, the proton signals of the PEG block were well resolved, indicating that the PEG was fully dissolved and mobile in aqueous media at rt. In contrast, the proton signals of the polycarbonate chains were significantly attenuated and difficult to detect, reflecting the restricted mobility and limited solvation of this hydrophobic segment. This observation supported the formation of micelles with a polycarbonate compact core and a stabilizing PEG corona. Upon heating, the signals corresponding to polycarbonate progressively gained intensity and resolution. This provided evidence of an increased mobility of the protons as a consequence of extensive hydration of the polycarbonate chains. Upon cooling, all the changes reversed to the initial spectra (Figure S32, Supporting Information). For PEG_45_‐*b*‐P(MPCU)_23_, the PEG signal was split above 60 °C although the overall intensity remained constant. This splitting can be due to a slight change in the polarity of the microenvironment or interactions between the two blocks when large aggregates were formed at elevated temperatures.^[^ ^42^ ^]^ However, for PEG_45_‐*b*‐P(MACU)_22_, the shape and intensity of methylenic signal of the PEG block did not change. Nevertheless, additional ROE experiments recorded for PEG_45_‐*b*‐P(MACU)_22_ at elevated temperature (70 °C) demonstrated that the two blocks are in close spatial proximity (less than 0.5 nm), as evidenced by weak cross‐peaks between their signals (Figure S31, Supporting Information).

Variable temperature spectra were used to obtain information about the apparent hydration degree of the polycarbonate block. Signal labeled as *d*, corresponding to methyl protons of the polycarbonate main chain, and signals labeled as *g* and *j*, corresponding to side chain protons located at different distances from the ureido group were selected and their normalized integrated intensity was determined using the PEG signal labeled as *b* as reference. It was assumed that PEG block is well solvated in D_2_O and its overall intensity remains constant along all temperature range.^[^ ^14^ ^]^ Then, the normalized integrated intensities of these signals at a given temperature (I^T^) in D_2_O were compared to their normalized integrated intensities in DMSO‐d_6_ (I^DMSO^), where the polycarbonate was fully dissolved. In this context, it can be assumed that when the I^T^/I^DMSO^ ratio equals 1, the polymer is fully solvated, which can be related to an apparent hydration degree of 100% at the given temperature T.^[^ ^12^ ^]^ In general, the I^T^/I^DMSO^ parameter showed a clear and strong dependence on the temperature and significantly increased upon heating, regardless of the selected protons (Figure 5c,d). Hence, as the temperature increased, the polycarbonate block became progressively more hydrated as would have been expected from an UCST polymer. A broad transition in the apparent hydration degree was observed between 40 and 60 °C, which closely correlates with the temperature‐dependent evolution of the *D_h_* recorded by DLS.

For PEG_45_‐*b*‐P(MACU)_22_, I^30^/I^DMSO^ values ranged between 0.12 and 0.21. Protons *j*, located adjacent to the ureido, showed the highest value, while protons *d*, located at the backbone, exhibited the lowest (Figure 5c). These results are consistent with literature reports indicating that groups closer to hydrophilic units exhibit higher apparent hydration degrees than those further away and also with the fact that the backbone groups experience stronger aggregation within the hydrophobic domains leading to a reduced mobility compared to side chain ones.^[^ ^12^, ^43^ ^]^ The differences between signals were even more pronounced for PEG_45_‐*b*‐P(MPCU)_23_ where I^30^/I^DMSO^ for protons *g* and *j*, considered together, was 0.42, while for protons *d* was 0.19 (Figure 5d). Final I^80^/I^DMSO^ values were quite similar in all cases, nearing 0.95. Furthermore, the temperature‐dependent evolution of the I^T^/I^DMSO^ parameter varied with the selected protons, with the apparent hydration degree increasing faster for groups closer to the ureido moiety. All this suggests that the number of pendant hydrophilic ureido groups significantly influences segmental mobility and the hydration of the micellar core. This might be likely related to the above‐mentioned lower hydrophobicity of MPCU repeating units.

**Figure 6:**
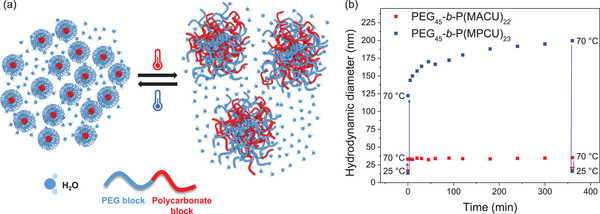
a) Schematic representation of the thermoresponse of PEG_45_‐*b*‐P(MACU)_22_ and PEG_45_‐*b*‐P(MPCU)_23_ in water promoted by the UCST behavior of the polycarbonate segment functionalized with ureido groups. b) Evolution of average number *D_h_* of PEG_45_‐*b*‐P(MACU)_22_ and PEG_45_‐*b*‐P(MPCU)_23_ dispersions (1.0 mg mL^−1^) as the samples were rapidly heated from 25 to 70 °C, maintained at 70 °C, and then cooled back to 25 °C.

The experiments discussed so far revealed two seemingly divergent conclusions. On one hand, changes in turbidity and DLS measurements apparently indicated a behavior that is not fully consistent with UCST characteristics. On the other hand, NMR data clearly demonstrated that the polycarbonate segment of the micellar core experiences a significant increase in hydration upon heating, which aligns closely with UCST‐like behavior, although without complete solubilization of the micelles into unimers. In the context of self‐assembled UCST polymers, few studies have unambiguously reported an increase in particle size with temperature. Augé et al. reported a swelling process of UCST polymer micelles before their dissociation upon heating above the T_cp_.^[^ ^13^ ^]^ According to Augé et al., swelling of the micelles due to water uptake is accompanied by a reduction in mass density, a decrease in the refractive index, and a reduction in light scattering intensity (i.e., an increase in transmittance). Therefore, in our case, the occurrence of only a swelling process is unlikely, particularly for PEG_45_‐*b*‐P(MPCU)_23_ in which a larger *D_h_* was detected above the transition temperature, accompanied by an increase in turbidity. Instead, swelling and fusion of the hydrated micelles into loose aggregates of a larger size, driven by the UCST‐like behavior of the ureido polycarbonate, may provide a more suitable explanation, aligning more closely with the work of Baddam et al.^[^ ^14^ ^]^ This was also supported by SAXS measurements above 55 °C, where the scattering objects were no longer defined as spherical micelles, but as rather loose and larger aggregates. On this basis, we assume that below the transition temperature, BCs self‐assemble into stable and small micelles. Above the transition temperature, the ureido polycarbonate block becomes more hydrophilic and the micelle core becomes progressively hydrated. Upon heating, BCs do not dissolve; instead, the hydrated micelles collapse into larger hydrated and loose aggregates with the process being reversible and dependent on the polymer concentration (**Figure** **6**a).

Swelling and collapse of the micelles should also explain the transmittance and *D_h_* evolution on heating/cooling dispersions of PEG_45_‐*b*‐P(MPCU)_23_ at low concentration (1.0 mg mL^‒1^). Swelling and fusion of the micelles can be observed as a dynamic process that evolves over time toward an equilibrium state as corroborated by analyzing the time‐dependent evolution of *D_h_* at 70 °C (Figure 6b; Figure S33, Supporting Information). When the dispersion was rapidly heated from 25 to 70 °C and maintained at this temperature up to 6 h, *D_h_* showed a sharp initial increase that rapidly slowed down, approaching a constant value. The initial values were recovered by cooling back to 25 °C. This dynamic process may explain the temperature‐dependent turbidity and DLS evolution upon cooling from 80 to 90 °C, if the equilibrium has not yet been reached, swelling and fusion may continue. When the same study was approached with PEG_45_‐*b*‐P(MACU)_22_, the *D_h_* increased to the final value and remained constant thereafter. The different behavior of PEG_45_‐*b*‐P(MPCU)_23_ can be attributed to the higher hydrophilicity of the MPCU repeating unit. Due to the reversibility of the process, the UCST‐like transition of these BCs can be observed as a transition from micelles to physical polymeric nanogels. In the highly hydrated nanometric condensates, the hydrated cross‐linked polymeric network is stabilized by non‐covalent interactions, primarily hydrophobic interactions between alkyl chains of the polycarbonate scaffold and hydrogen bonds through water bridges between the ureido groups thus avoiding the complete solubilization.

PEG_45_‐*b*‐P(MPCU)_n_ with longer DP for polycarbonate chain were examined to analyze how the length of this block affects thermoresponsiveness (Figure S34, Supporting Information). The first observation was that upon heating the PEG_45_‐*b*‐P(MPCU)_98_ vesicles dispersion, a precipitate formed at the bottom of the cuvette, even at a concentration of 1.0 mg mL^‒1^. This precipitate did not redisperse upon subsequent cooling or reheating of the aqueous dispersion. The loss of thermoresponse, on increasing the length of hydrophobic segment, was also reported by Buksa et al.^[^ ^44^ ^]^ As it was stated, PEG_45_‐*b*‐P(MPCU)_46_ was self‐assembled by the co‐solvent method into larger spherical and cylindrical micelles resulting in cloudy dispersion. During the heating of the polymer dispersion at 1.0 mg mL^−1^, the transmittance gradually decreased above 65 °C, accompanied by a gradual increase of the apparent number *D_h_* from 58 ± 18 nm at 20 °C to 110 ± 37 nm at 80 °C. Upon cooling, the transmittance sharply recovered its initial values at 30 °C. DLS revealed two number size distributions, a large size one coincident with that of the initial sample and a smaller one with a *D_h_* of 22 ± 4 nm at 20 °C. Subsequent turbidity curves recorded during heating differed from the first but were reproducible and similar to the cooling curves. These differences between the first and subsequent heating curves might be attributed to changes in self‐assembled morphologies generated after the initial heating. Unlike the PEG_45_‐*b*‐P(MPCU)_23_ dispersion, the phase transition occurred at lower temperatures and the growth of self‐assemblies was lower above the transition temperature.

### 2.5 Encapsulation and Temperature Induced Release of Curcumin

**Figure 7:**
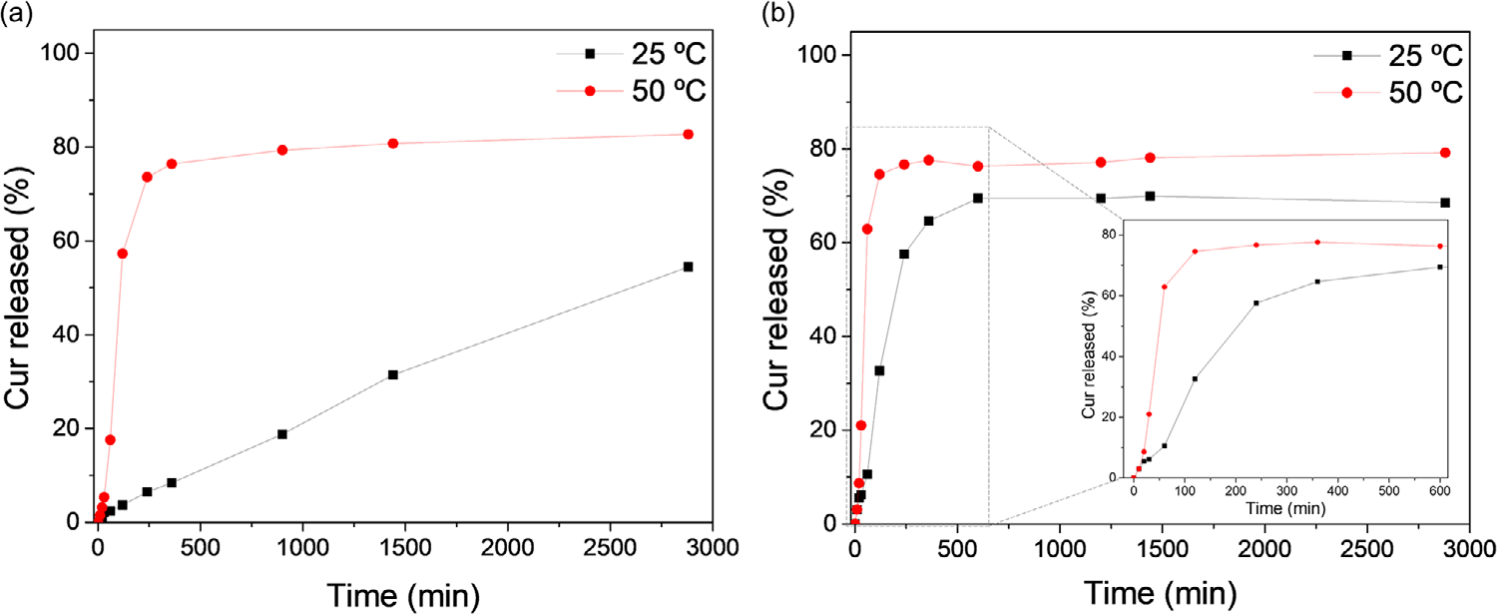
Cur release profiles over time from a) PEG_45_‐*b*‐P(MACU)_22_ and b) PEG_45_‐*b*‐P(MPCU)_23_ nanocarriers in water at 0.1 mg mL^−1^ polymer concentration at 25 and 50 °C.

After evaluation of the thermoresponsive behavior, the thermal‐triggered release of drugs initially encapsulated into the PEG_45_‐*b*‐P(MACU)_22_ and PEG_45_‐*b*‐P(MPCU)_23_ micelles at rt was evaluated using Curcumin (Cur) as a hydrophobic model drug. Cur has anti‐inflammatory, anticancer, and antioxidant properties, but its poor solubility (below 1 µg mL^−1^ in water), stability, and bioavailability limit its therapeutic use, which can be improved by encapsulating Cur in liposomes or polymer self‐assemblies.^[^ ^45^, ^46^, ^47^ ^]^ Cur was encapsulated during micellar self‐assembly at rt by the co‐solvent method using a 1.5:10.0 mg mg^−1^ Cur/polymer ratio (see details in Supporting Information). For both polymers, drug loading (DL) was ≈9.5%, corresponding to a Cur concentration of approx. 115 µg mL^−1^. Cur release at 25 and 50 °C was monitored by UV–vis spectroscopy using a polymer concentration of 0.1 mg mL^−1^, which is still above the CAC, to avoid the saturation of the absorbance at the maximum absorption wavelength of the Cur (436 nm) (**Figure** **7**; Figure S35, Supporting Information). During the experiments, a yellow solid of Cur was progressively deposited at the bottom of the cuvette, indicating Cur release. Consequently, the release was examined by measuring the decrease in absorbance at 436 nm, considering that values could be underestimated due to the increase of scattered light due to suspended particles of Cur. For Cur/PEG_45_‐*b*‐P(MACU)_22_ significant differences were observed in the Cur release profiles at 25 and 50 °C (Figure 7a). At 25 °C, a sustained release was recorded up to 2 days, reaching a 55% release. The release was found to be largely accelerated at 50 °C, being 80% after 4 h. However, for PEG_45_‐*b*‐P(MPCU)_23_, although the trigger effect of temperature was also observed, similar release profiles were recorded at both temperatures (Figure 7b). At 25 °C, approx. 70% of the loaded Cur was released after 10 h, and ≈75% after 2 h at 50 °C. Additionally, the Cur release was studied at the physiological temperature of 37 °C, showing an intermediate release behavior between those observed at 50 and 25 °C, as expected (Figure S36, Supporting Information).

Therefore, for both polymers, Cur release was temperature‐dependent and triggered by the transition into hydrated nanogel‐like systems above the UCST‐like transition. Besides, the release rate was higher for PEG_45_‐*b*‐P(MPCU)_23_, even in micelles at 25 °C likely due to the higher hydrophilicity and increased hydration ability than the PEG_45_‐*b*‐P(MACU)_22_ counterpart.

## 3 Conclusion

In conclusion, degradable ureido functionalized homopolycarbonates have been successfully synthesized by ROP and thiol‐ene/yne post‐polymerization functionalization. The results demonstrate that increasing the density of ureido groups promotes UCST behavior in P(MPCU)_22_ although solubilization of the polymer requires high temperature (T_cp_ > 90 °C). This behavior is attributed to the increased hydrophilicity of P(MPCU)_22_ compared to P(MACU)_22_.

The synthetic strategy has been successfully extended to prepare the corresponding BCs, PEG_45_‐*b*‐P(MACU)_22_ and PEG_45_‐*b*‐P(MPCU)_n_, with PEG hydrophilic block. At rt, these copolymers are amphiphilic in nature and can self‐assemble in water using different methodologies. The morphology and size of these self‐assemblies are influenced by both the hydrophobic/hydrophilic balance and the self‐assembly method.

PEG_45_‐*b*‐P(MACU)_22_ and PEG_45_‐*b*‐P(MPCU)_23_ micelles exhibit a unique thermoresponsive behavior, which was not initially expected. Thus, the UCST‐transition of the ureido polycarbonate segment does not lead to micellar disassembly into solvated unimers at the transition temperature, likely because the polymers remain insufficiently hydrophilic even above this temperature. Instead, the polymeric micelles evolve into larger, strongly hydrated aggregates or nanogel‐like systems a process that is fully reversible. Besides, the thermoresponse depends on the composition of the BCs and the length of the polycarbonate block. Doubling the number of ureido groups, large nanogel‐like condensates are observed above the transition temperature, probably due to the higher hydrophilicity of the polymer. Conversely, self‐assemblies with longer polycarbonate block partially lost their thermoresponsive behavior. Finally, the potential of these systems for the encapsulation and thermal‐triggered release of curcumin as hydrophobic model drug have been demonstrated.

Overall, this study highlights the potential of UCST‐responsive polycarbonate‐based block copolymers for advanced applications, such as smart and degradable nanocarriers, where precise control of self‐assembly and thermoresponsive properties is crucial.

## Conflict of Interest

The authors declare no conflict of interest.

## Supporting information



Supporting Information

Supplemental Video 1

## Data Availability

The data that support the findings of this study are available from the corresponding author upon reasonable request.
