# Supplementary material for: Degradable Ureido‐Polycarbonate Block Copolymers with a Complex UCST Thermoresponse
Source: Macromol Rapid Commun. 2025 Mar 22;46(14):2500029. doi: 10.1002/marc.202500029 (PMC12272536; doi:10.1002/marc.202500029)
Supplement: Supplementary file 1 — Supporting Information [file MARC-46-2500029-s002.pdf]

Macromolecular Rapid Communications

Supporting Information

for *Macromol. Rapid Commun.*, DOI 10.1002/marc.202500029

Degradable Ureido-Polycarbonate Block Copolymers with a Complex UCST Thermoresponse

*Javier Martin-Martin, Miriam Abad, Xabier Lopez de Pariza, Tiberio A. Ezquerra, Aurora Nogales, Haritz Sardon, Víctor Sebastián, Luis Oriol and Milagros Piñol**

Supporting Information

Degradable Ureido-Polycarbonate Block Copolymers with a Complex UCST Thermoresponse

Javier Martin-Martin, Miriam Abad, Xabier Lopez de Pariza, Tiberio A. Ezquerra, Aurora Nogales, Haritz Sardon, Víctor Sebastián, Luis Oriol and Milagros Piñol

J. Martin-Martin, M. Abad, L. Oriol, M. Piñol
Instituto de Nanociencia y Materiales de Aragón (INMA), CSIC-Universidad de Zaragoza, Zaragoza 50009, Spain. E-mail: mpinol@unizar.es
Departamento de Química Orgánica, Facultad de Ciencias, Universidad de Zaragoza, Pedro Cerbuna, 12, Zaragoza 50009, Spain.

X. Lopez de Pariza, H. Sardon
POLYMAT and Department of Polymers and Advanced Materials: Physics, Chemistry and Technology, Faculty of Chemistry, University of the Basque Country UPV/EHU, Donostia-San Sebastián 20018, Spain.

T. A. Ezquerra, A. Nogales
Instituto de Estructura de la Materia, IEM-CSIC, C/Serrano, 121, Madrid 28006, Spain.

V. Sebastián
Instituto de Nanociencia y Materiales de Aragón (INMA), CSIC-Universidad de Zaragoza, Zaragoza 50009, Spain.
Department of Chemical Engineering and Environmental Technologies, University of Zaragoza, Zaragoza 50018, Spain.
Networking Research Center on Bioengineering, Biomaterials and Nanomedicine (CIBER-BBN), 28029 Madrid, Spain.
Laboratorio de Microscopías Avanzadas, Universidad de Zaragoza, 50018 Zaragoza, Spain.

Table of contents

1. Materials 3

2. Characterization techniques 3

3. Experimental procedures 4

3.1. Synthesis of 1-(2-mercaptoethyl)urea U-SH 4

3.2. General procedure for the synthesis of homopolycarbonates by ROP 8

3.3. General procedure for the synthesis of BCs by ROP 10

3.4. General procedure for the thiol-ene or thiol-yne additions initiated by UV light 15

3.5. Synthesis and characterization of bis-MPA-U 26

4. Turbidity measurements 28

5. Variable temperature DLS measurements 28

6. TEM images 28

7. Variable temperature ^{1}H NMR experiments 28

8. Variable temperature SAXS experiments 28

9. Preparation of self-assemblies in water 28

10. Determination of the Critical Aggregation Concentration (CAC) 29

11. Degradability studies of P(MPCU)$_{22}$ in water 29

12. Preparation and quantification of Cur loaded into polymer micelles 30

13. In vitro release of Cur from polymer self-assemblies 30

14. Supplementary figures and tables 31

14.1. MALDI-TOF analysis of homopolycarbonate and BC precursors 33

1. Materials

Cyclic carbonate monomers, 5-methyl-5-allyloxycarbonyl-1,3-dioxan-2-one (MAC) and 5-methyl-5-propargyloxycarbonyl-1,3-dioxan-2-one (MPC), were synthesized as reported in the literature.[1] 1-(3,5-Bis(trifluoromethyl)-phenyl)-3-cyclohexylthiourea (TU) was synthesized as reported in the literature.[2] Poly(ethylene glycol) methyl ether, PEG_{45}-OH (M_n provided by the supplier was verified by MALDI-TOF mass analysis M_n = 1942 g mol^{-1}, using Polytools Bruker software, and according to SEC analysis *Đ* was 1.02, using the same conditions as the employed for BCs see Figure S18) was purchased from Sigma-Aldrich, dried three times by azeotropic distillation in toluene and stored under vacuum at 70 ºC overnight before use. Benzyl alcohol was stored over 4 Å molecular sieves. DCM was distilled from $CaCl_2$. Curcumin was purchased in MedChemExpress. Phosphate buffered saline (PBS) from Gibco™ pH 7.4 (1X) was purchased from Fisher Scientific. All other reagents and solvents were purchased from Sigma-Aldrich and Fisher Scientific and were used as received.

2. Characterization techniques

Infrared (IR) spectra were recorded on a Bruker Vertex 70 spectrophotometer using KBr pellets at product concentration of 1-2 % (w/w). ^{1}H NMR and ^{13}C NMR spectra were registered on a Bruker AV-400 spectrometer using either $CDCl_3$, DMSO-d_6 or D_2O. Rotating-frame nuclear Overhauser effect (ROE) spectra were registered in a Bruker AV-500 spectrometer with a mixing time of 400 ms. Matrix-assisted laser desorption ionization mass spectroscopy (MALDI-TOF-MS) was performed on an Autoflex Bruker mass spectrometer using dithranol or trans-2-[3-(4-tert-butylphenyl)-2-methyl-2-propenylidene]malononitrile (DCTB) as matrix. Size exclusion chromatography (SEC) was carried out on a Water Alliance 2695 liquid chromatography system equipped with a Waters 2424 evaporative light scattering detector using two columns connected in series, Styragel (HR2, 500 Å and HR4, 10^4 Å) from Waters (5 µm, 7.8×300 mm), and HPLC grade THF as eluent at a flow rate of 1.0 mL min^{-1} at 35 ºC. Poly(methyl methacrylate) narrow molecular weight standards were used to calibrate the system. Thermogravimetric analysis (TGA) was performed at heating rate of 10 ºC min^{-1} under nitrogen atmosphere on a SDT2960 Simultaneous DTA-TGA from TA instruments. Decomposition temperature associated to mass loss was read as the onset point in the weight loss curve. Differential scanning calorimetry (DSC) was performed on a DSC Q2000 calorimeter from TA instruments in the range of –50 to 130 °C at scanning rate of 10 ºC min^{-1}, using approx. 2.0 mg of the sample sealed in aluminium pans. The glass transition temperature

(T_g) was determined at the half height of baseline jump and the melting temperature (T_m) was determined at the maximum of the corresponding peak. Turbidity measurements during self-assembly by co-solvent method were conducted on ATI-Unicam UV4-200 spectrophotometer as transmittance modification at 650 nm. Turbidity measurements were conducted on a Cary 6000i UV-Vis-NIR spectrophotometer equipped with a Peltier temperature controller. Fluorescence measurements were performed on a Perkin Elmer LS 50B fluorescence spectrophotometer. Dynamic light scattering (DLS) measurements were carried out in a Malvern Instrument Nano ZS using a He-Ne laser with a 633 nm wavelength and a detector angle of 173 º. The size measurements were registered three times to ensure reproducibility. Transmission Electron Microscopy (TEM) was carried out on a FEI TECNAI G20 electron microscope operating at 200 kV. Small Angle X-ray Scattering (SAXS) experiments were performed at beamline BL11 NCD (ALBA, Spain) using a wavelength of 0.1 nm. The SAXS detector, a photon counting Dectris 2D detector (model Pilatus 1 M), was located at 3.83 m distance from the sample position. For experiments performed upon heating and cooling ramps, a capillary containing the sample was inserted into a Linkam THM600 hot stage. Log *P* of repeating units of polycarbonates was determined by Chemdraw professional 15.0.

3. Experimental procedures

3.1. Synthesis of 1-(2-mercaptoethyl)urea U-SH

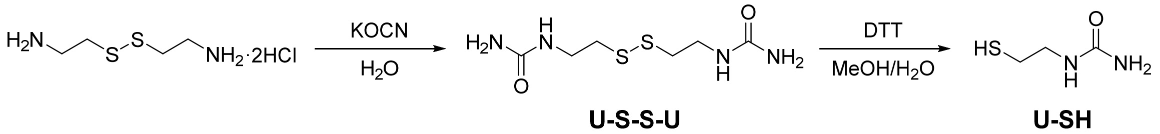

Scheme S1. Synthesis of U-SH

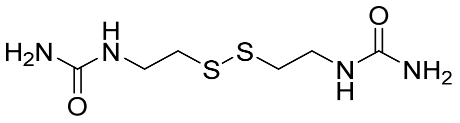

Synthesis and characterization of U-S-S-U disulfide. Potassium cyanate (10.37 g, 122.77 mmol) was added to a solution of cystamine dihydrochloride (12.00 g, 51.15 mmol) in water (100 mL). The reaction was stirred at rt for 24 h. Then, water was removed under vacuum. The crude was purified by flash column chromatography on silica gel, eluted with a mixture of DCM/methanol (MeOH) (8:2). The U-S-S-U disulfide was obtained as a white solid. Yield: 62%. FTIR (KBr), (ν, cm^{-1}): 3426, 3358, 3213 (N-H^{st}), 2920, 2870 (Csp^3-H^{st}), 1647, (C=O^{st}), 1603, 1553 (N-H^{δ}). ^1H NMR (400 MHz, DMSO-d_6) δ (ppm): 6.20 (t, *J* =

6.0 Hz, 2H, N**H₂**), 5.55 (s broad, 4H, N**H**), 3.25 (q, *J* = 6.6 Hz, 4H, NHC**H₂**), 2.73 (t, *J* = 6.8 Hz, 4H, C**H₂**S). ^{13}C NMR (100 MHz, DMSO-d_6) δ (ppm): 158.6, 38.3, 38.3.

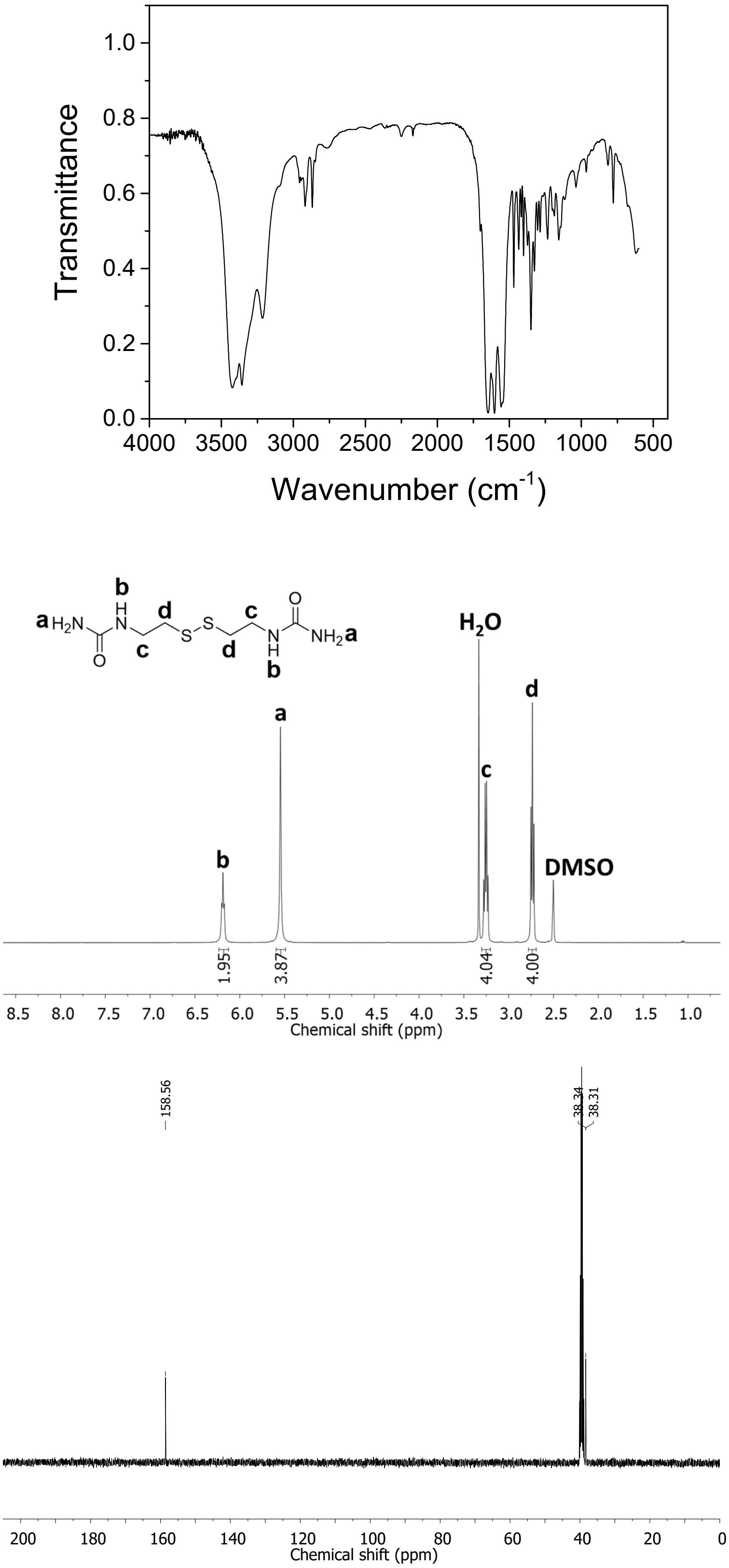

Figure S1. FTIR (top), ^{1}H NMR (middle) and ^{13}C NMR (bottom) spectra of U-S-S-U

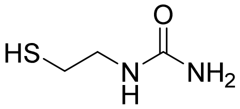

Synthesis and characterization of U-SH thiol. The U-S-S-U disulfide (550.0 mg, 2.30 mmol) was dissolved in MeOH/H_2O (1:1) (80 mL) and the solution was purged with argon. Then, DL-dithiothreitol (DTT) (562.5 mg, 3.65 mmol) was added to the mixture. The reaction was stirred under argon atmosphere at rt for 24 h. Afterwards, the solvent was evaporated under reduced pressure. The crude was purified by flash column chromatography on silica gel, using as eluent a mixture of DCM/MeOH (97:3) and increasing progressively the polarity to DCM/MeOH (93:7). The U-SH thiol was obtained as a white solid. Yield: 85%. FTIR (KBr), (ν, cm^{-1}): 3415, 3353, 3214 (N-H^{st}), 2940 (Csp^3-H^{st}), 1649, (C=O^{st}), 1601, 1540 (N-H^{δ}). ^1H NMR (400 MHz, DMSO-d_6) δ (ppm): 6.11 (t, J = 5.4 Hz, 1H, N**H**), 5.47 (s, 2H, N**H_2**), 3.10 (q, J = 6.5 Hz, 2H, C**H_2**NH), 2.47 (m, 2H, SHC**H_2**), 2.23 (t, J = 8.0 Hz, 1H, S**H**). ^{13}C NMR (100 MHz, $CDCl_3$) δ (ppm): 158.5, 42.7, 24.5.

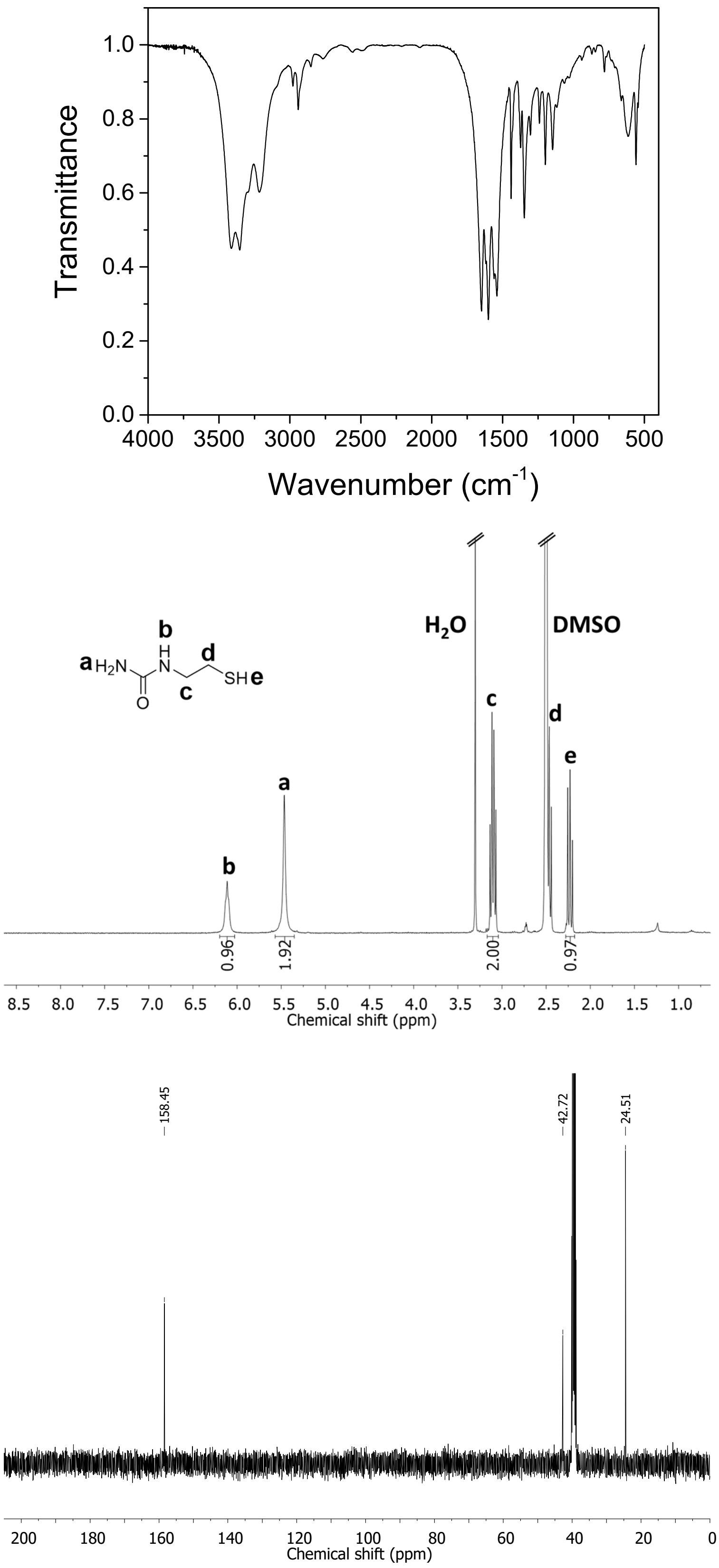

Figure S2. FTIR (top), ^{1}H NMR (middle) and ^{13}C NMR (bottom) spectra of U-SH

3.2. General procedure for the synthesis of homopolycarbonates by ROP

The polymerization was performed employing Schlenk line techniques. Benzyl alcohol (1.00 mmol), DBU (0.25 mmol) and TU (1.25 mmol) were dissolved in anhydrous DCM under argon atmosphere. Then, a solution of MAC or MPC (25.00 mmol) in anhydrous DCM was added *via* cannula to the initiator solution. The concentration of monomer was 0.5 M. The polymerization was performed at 35 ºC for 2-3 h until achieving a monomer conversion of 80% monitored by ^{1}H NMR. Then, the reaction was quenched with benzoic acid (5.00 mmol to DBU) and precipitated into cold hexane. The crude was purified by precipitation into cold MeOH three times. The homopolycarbonates were obtained as transparent viscous oils.

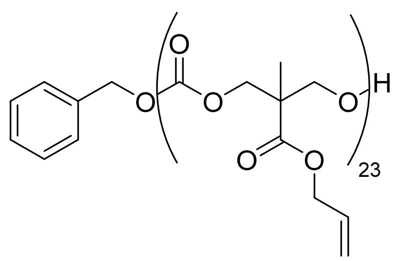

Characterization of P(MAC)23. The $P(MAC)_{23}$ was synthesized following the aforementioned general procedure, employing the following reactants and solvents: MAC (1.50 g, 7.50 mmol), benzyl alcohol (31.3 µL, 0.30 mmol), DBU (11.3 µL, 0.08 mmol), TU (138.9 mg, 0.38 mmol) and DCM (15 mL). The polymer was isolated as a colourless glassy. Yield: 40%. FTIR (KBr), (ν, cm^{-1}): 3090 (Csp^2-H_2st), 2981, 2893 (Csp^3-H^{st}), 1753 ($C{=}O^{st}$), 1651 ($C{=}C^{st}$). ^{1}H NMR (400 MHz, $CDCl_3$) δ (ppm): 7.39-7.31 (m, $C_6\mathbf{H_5}CH_2$), 5.95-5.79 (m, $CH_2C\mathbf{H}CH_2$), 5.36-5.17 (m, $CH_2CHC\mathbf{H_2}$), 5.14 (s, $C_6H_5C\mathbf{H_2}$), 4.66-4.57 (m, $C(O)OC\mathbf{H_2}CH$), 4.38-4.22 (m, $OC(O)OC\mathbf{H_2}$), 3.76-3.67 (m, $C\mathbf{H_2}OH$), 2.48 (t, *J* = 6.7 Hz, O**H**), 1.28-1.24 (m, $CC\mathbf{H_3}$), 1.22 (s, $CC\mathbf{H_3}$).

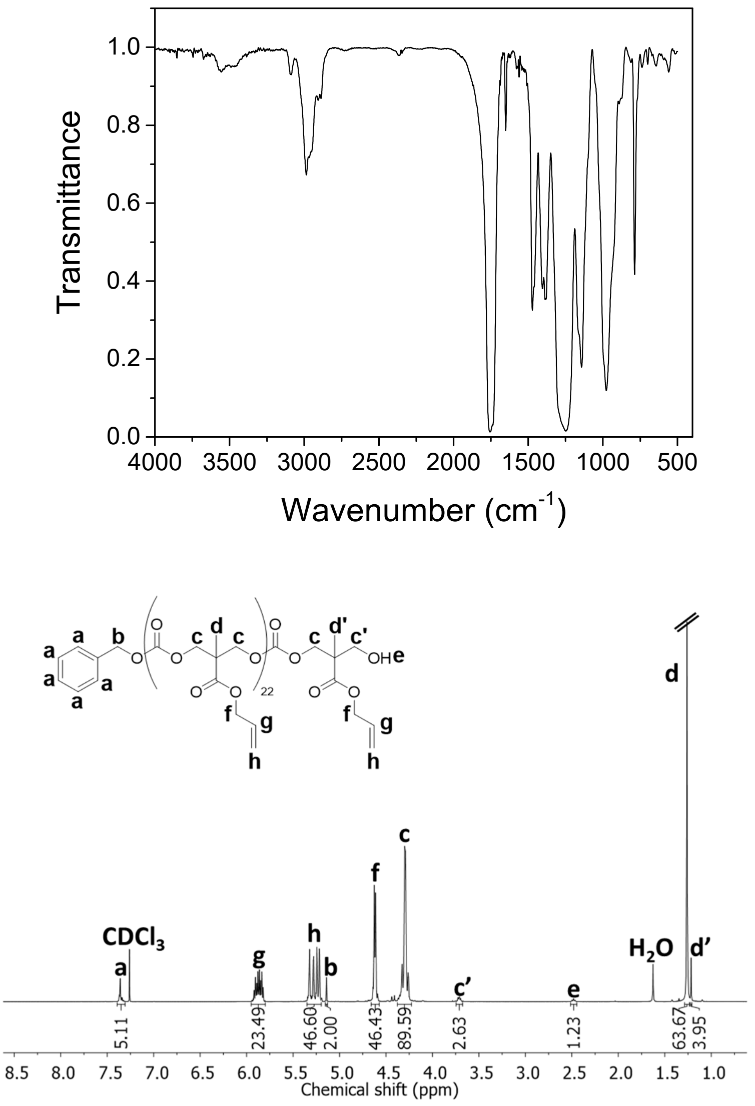

Figure S3. FTIR (top) and ^{1}H NMR (down) spectra of P(MAC)$_{23}$

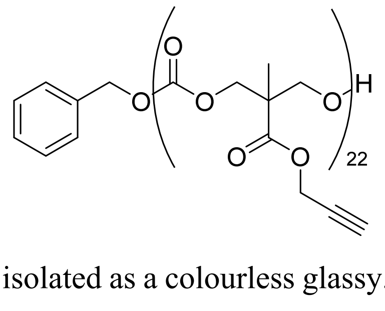

Characterization of P(MPC)$_{22}$. The P(MPC)$_{22}$ was synthesized following the aforementioned general procedure, employing the following reactants and solvents: MPC (1.00 g, 5.05 mmol), benzyl alcohol (23.0 µL, 0.22 mmol), DBU (7.6 µL, 0.05 mmol), TU (89.2 mg, 0.25 mmol) and DCM (10 mL). The polymer was isolated as a colourless glassy. Yield: 60%. FTIR (KBr), (ν, cm^{-1}): 3293 (Csp-Hst), 2981 (Csp^3-Hst), 2130 (C≡Cst), 1753 (C=Ost). ^{1}H NMR (400 MHz, $CDCl_3$) δ (ppm): 7.39-7.32 (m, C_6**H_5**CH_2), 5.15 (s, C_6H_5C**H_2**), 4.75-4.68 (m, C(O)OC**H_2**CCH), 4.38-4.24 (m, OC(O)OC**H_2**),

3.78-3.67 (m, C**H**$_2$OH), 2.56-2.49 (m, CH$_2$CC**H**), 2.46 (t, *J* = 2.4 Hz, CH$_2$CC**H**), 2.40 (t, *J* = 6.8 Hz, O**H**), 1.32-1.25 (m, CC**H**$_3$), 1.23 (s, CC**H**$_3$).

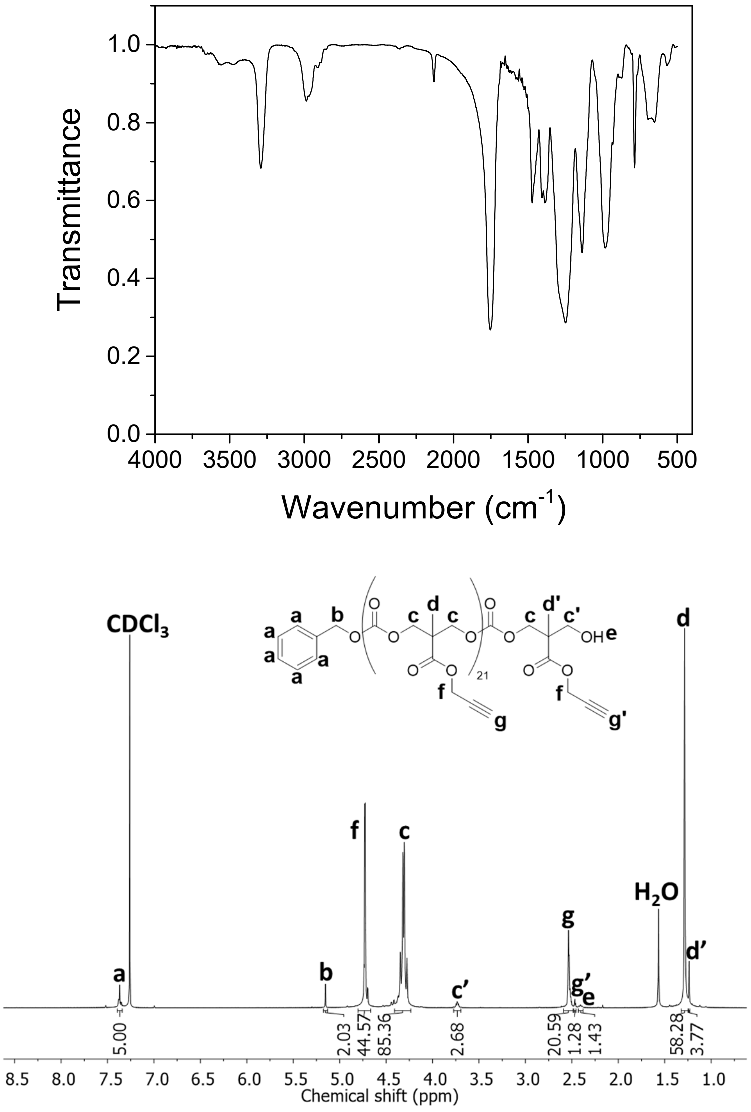

Figure S4. FTIR (top) and ^{1}H NMR (down) spectra of P(MPC)$_{22}$

3.3. General procedure for the synthesis of BCs by ROP

All polymerizations were conducted either in a glovebox or by Schlenk-line techniques. PEG$_{45}$-OH (1.00 mmol), DBU (1 mmol % relative to the monomer) and TU (5 mmol % relative to the monomer) were dissolved in anhydrous DCM under argon atmosphere. Then, a solution of MAC or MPC in anhydrous DCM was added *via* cannula to the initiator solution. The initial monomer concentration was 0.5 M. The fed molar ratios of each reagent are gathered in Table S1. The polymerization was conducted under stirring at 35 ºC until a monomer conversion of 80% was reached, as monitored by ^{1}H NMR spectroscopy, which typically requiered 2-3 h.

Then, the reaction was quenched with benzoic acid (5.00 mmol to DBU) and precipitated into cold hexane. Afterwards, the crude was purified by precipitation into cold diethyl ether three times.

Table S1. Summary of fed molar ratios of reactants relative to macroinitiator in the ROP of BCs

Polymer	PEG_{45}-OH	MPC	MAC	DBU	TU
PEG_{45}-*b*-$P(MAC)_{22}$	1	-	25	0.25	1.25
PEG_{45}-*b*-$P(MPC)_{23}$	1	25	-	0.25	1.25
PEG_{45}-*b*-$P(MPC)_{46}$	1	55	-	0.55	2.75
PEG_{45}-*b*-$P(MPC)_{98}$	1	105	-	1.05	5.25

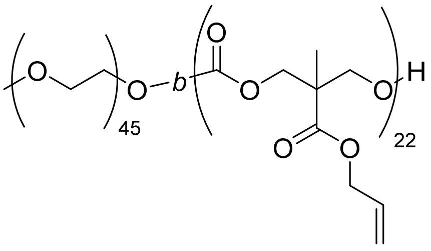

Characterization of PEG_{45}-b-$P(MAC)_{22}$. PEG_{45}-*b*-$P(MAC)_{22}$ was synthesized by Schlenk line technique, following the aforementioned general procedure, employing the following reactants and solvents: MAC (1.25 g, 6.25 mmol), PEG_{45}-OH (499.0 mg, 0.25 mmol), DBU (9.4 µL, 0.06 mmol), TU (115.7 mg, 0.31 mmol) and DCM (12 mL). The polymer was isolated as a colourless oil. Yield: 42%. FTIR (KBr), (ν, cm^{-1}): 3092 (Csp^2-H_2^{st}), 2940, 2883 (Csp^3-H^{st}), 1753 (C=O^{st}), 1646 (C=C^{st}). ^{1}H NMR (400 MHz, $CDCl_3$) δ (ppm): 5.93-5.80 (m, CH_2C**H**CH_2), 5.34-5.19 (m, CH_2CHC**H₂**), 4.61 (dt, *J* = 5.6, 1.3 Hz, C(O)OC**H₂**CH), 4.38-4.19 (m, OC(O)OC**H₂**), 3.76-3.51 (m, OC**H₂**C**H₂**O), 3.36 (s, CH_2OC**H₃**), 2.49 (t, *J* = 6.2 Hz, O**H**), 1.30-1.22 (m, CC**H₃**), 1.22 (s, CC**H₃**).

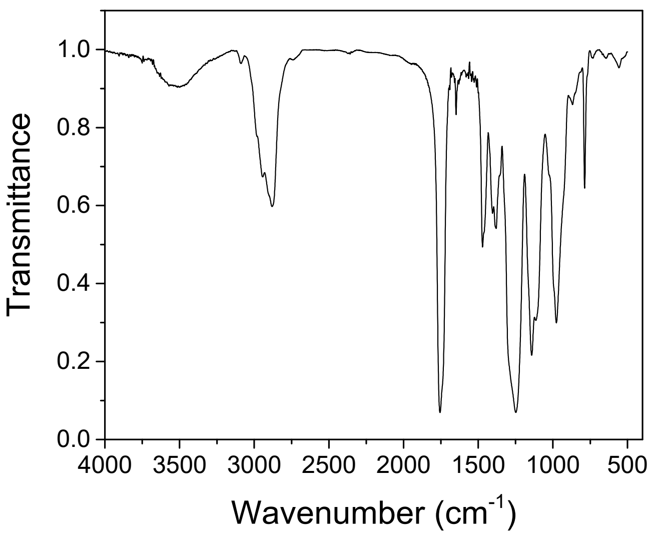

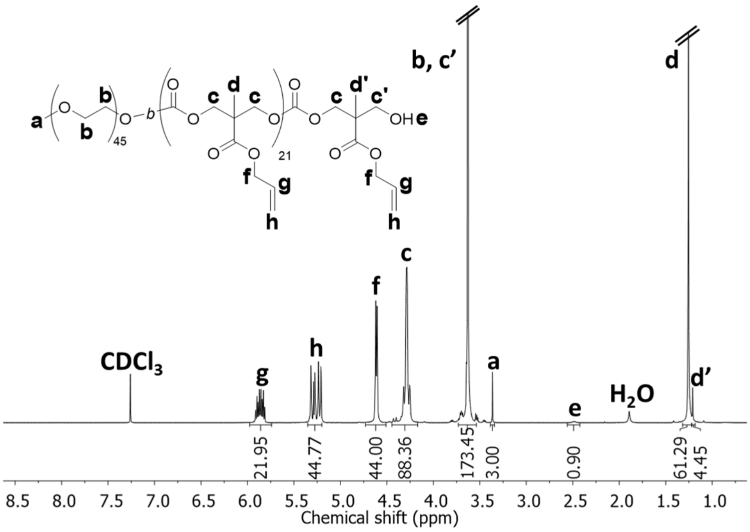

Figure S5. FTIR (top) and ^{1}H NMR (down) spectra of PEG45-*b*-P(MAC)22

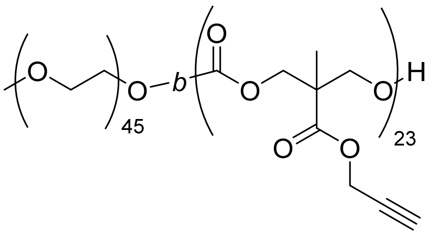

Characterization of PEG45-b-P(MPC)23. PEG45-*b*-P(MPC)23 was synthesized by Schlenk line technique, following the aforementioned general procedure, employing the following reactants and solvents: MPC (1.24 g, 6.26 mmol), PEG45-OH (499.0 mg, 0.25 mmol), DBU (9.4 µL, 0.06 mmol), TU (115.7 mg, 0.31 mmol) and DCM (12 mL). The polymer was isolated as a colourless oil. Yield: 51%. FTIR (KBr), (ν, cm^{-1}): 3289 (Csp-H^{st}), 2948, 2888 (Csp^3-H^{st}), 2130 (C≡C^{st}), 1753 (C=O^{st}). ^1H NMR (400 MHz, $CDCl_3$) δ (ppm): 4.72 (d, *J* = 2.4 Hz, C(O)OC**H2**CCH), 4.37-4.22 (m, OC(O)OC**H2**), 3.75-3.51 (m, OC**H2**C**H2**O), 3.37 (s, CH2OC**H3**), 2.53 (t, *J* = 2.6 Hz, CH2CC**H**), 2.42 (s broad, O**H**), 1.31-1.25 (m, CC**H3**), 1.23 (s, CC**H3**).

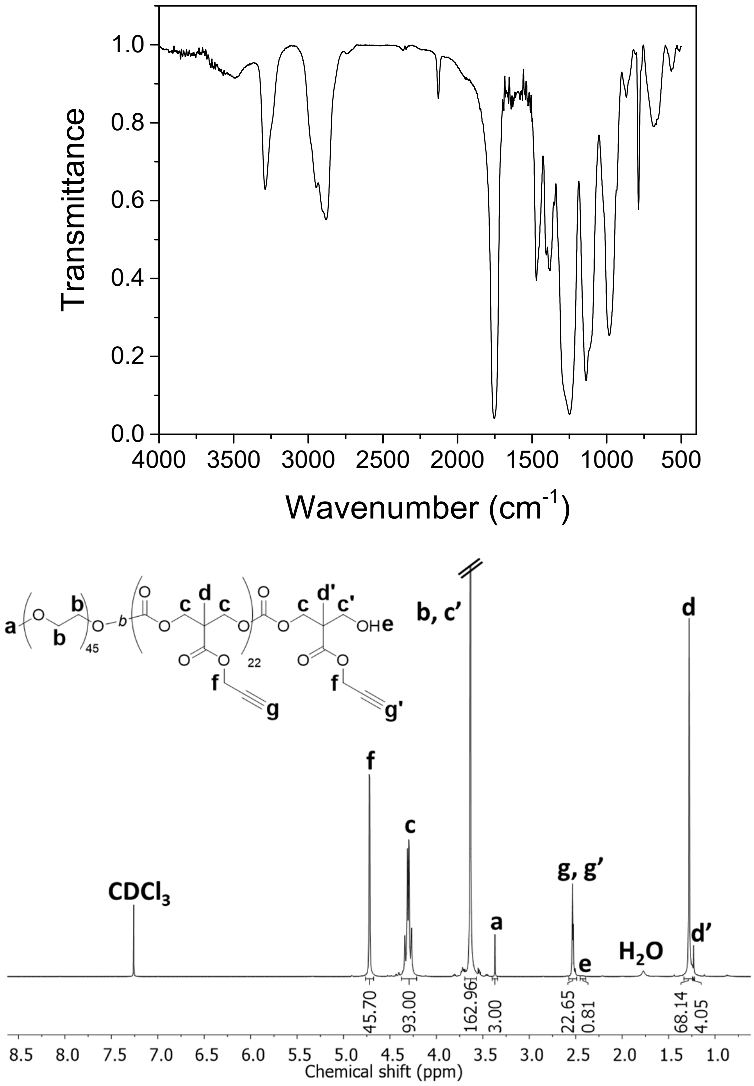

Figure S6. FTIR (top) and ^{1}H NMR (down) spectra of PEG$_{45}$-*b*-P(MPC)$_{23}$

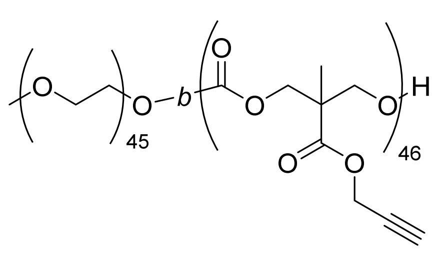

Characterization of PEG$_{45}$-b-P(MPC)$_{46}$. PEG$_{45}$-*b*-P(MPC)$_{46}$ was synthesized in a glovebox, following the aforementioned general procedure, employing the following reactants and solvents: MPC (1.20 g, 6.06 mmol), PEG$_{45}$-OH (220.4 mg, 0.11 mmol), DBU (9.0 μL, 0.06 mmol), TU (112.2 mg, 0.30 mmol) and DCM (12 mL). The polymer was isolated as a colourless oil. Yield: 57%. FTIR (KBr), (ν, cm^{-1}): 3291 (Csp-Hst), 2954, 2884 (Csp^3-Hst), 2131 (C≡Cst), 1751 (C=Ost). ^{1}H NMR (400 MHz, $CDCl_3$) δ (ppm): 4.75-4.70 (m, C(O)OC**H$_2$**CCH), 4.36-4.25 (m, OC(O)OC**H$_2$**), 3.68-3.57 (m, OC**H$_2$**C**H$_2$**O), 3.37 (s, CH$_2$OC**H$_3$**), 2.58-2.47 (m, CH$_2$CC**H**), 1.32-1.25 (m, CC**H$_3$**), 1.23 (s, CC**H$_3$**).

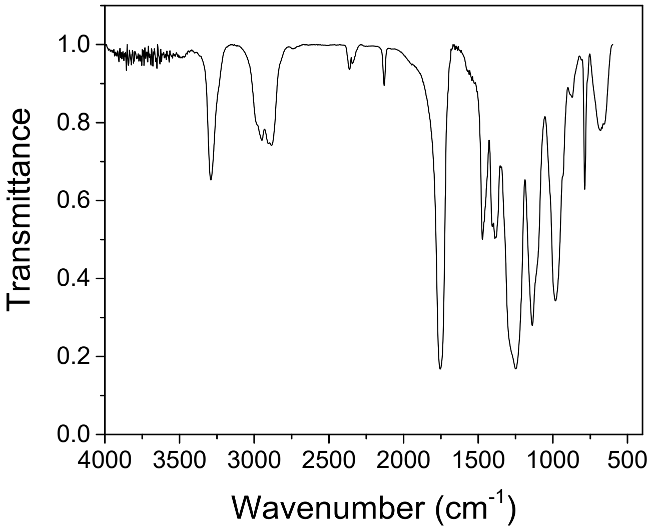

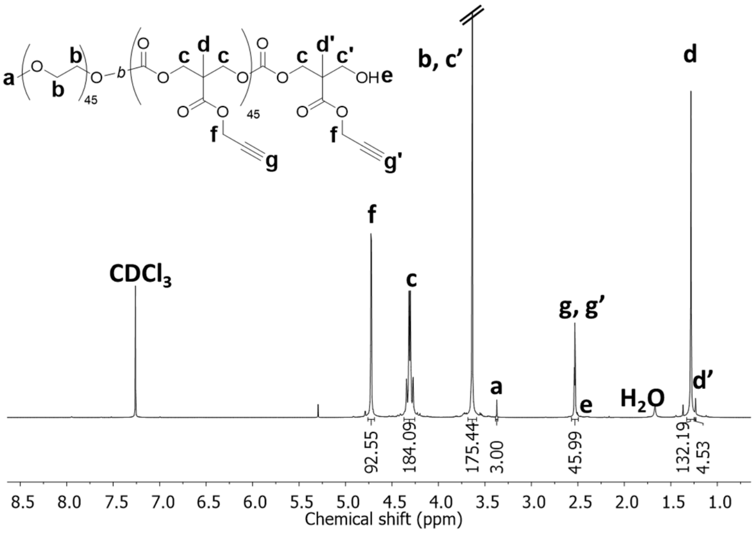

Figure S7. FTIR (top) and ^{1}H NMR (down) spectra of PEG_{45}-*b*-$P(MPC)_{46}$

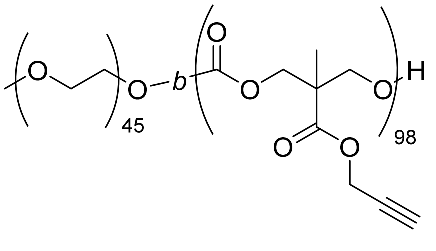

Characterization of PEG_{45}*-b-P(MPC)*$_{98}$. PEG_{45}-*b*-$P(MPC)_{98}$ was synthesized in a glovebox, following the aforementioned general procedure, employing the following reactants and solvents: MPC (1.50 g, 7.57 mmol), PEG_{45}-OH (144.2 mg, 0.07 mmol), DBU (11.3 µL, 0.08 mmol), TU (140.2 mg, 0.38 mmol) and DCM (15 mL). The polymer was isolated as a colourless glassy. Yield: 65%. FTIR (KBr), (ν, cm^{-1}): 3292 (Csp-H^{st}), 2963, 2903 (Csp^3-H^{st}), 2132 (C≡C^{st}), 1753 (C=O^{st}). ^{1}H NMR (400 MHz, $CDCl_3$) δ (ppm): 4.75-4.70 (m, C(O)OC$\mathbf{H_2}$CCH), 4.37-4.24 (m, OC(O)OC$\mathbf{H_2}$), 3.66-3.61 (m, OC$\mathbf{H_2}$C$\mathbf{H_2}$O), 3.37 (s, CH_2OC$\mathbf{H_3}$), 2.57-2.49 (m, CH_2CC**H**), 1.32-1.25 (m, C$\mathbf{H_3}$), 1.24 (s, C$\mathbf{H_3}$).

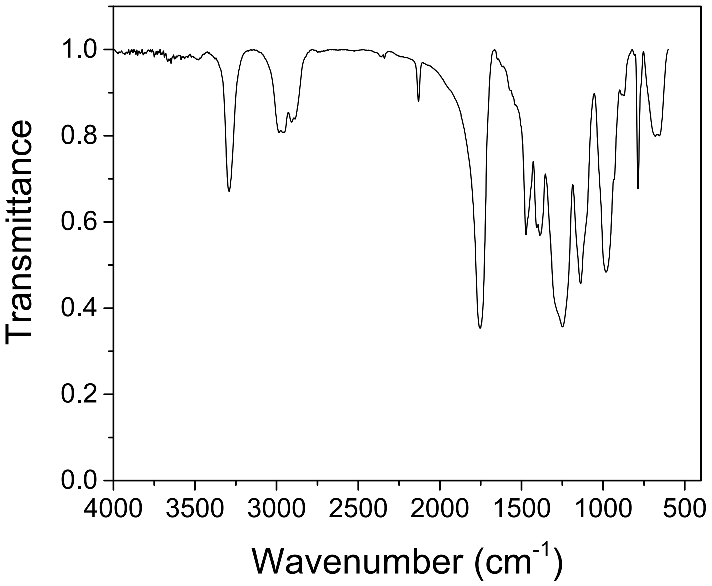

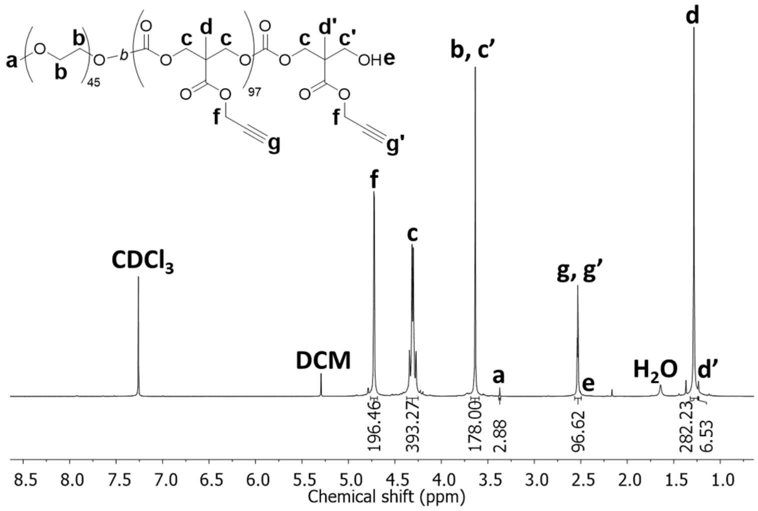

Figure S8. FTIR (top) and ^{1}H NMR (down) spectra of PEG$_{45}$-*b*-P(MPC)$_{98}$

3.4. General procedure for the thiol-ene or thiol-yne additions initiated by UV light

The allyl or propargyl polymer was dissolved in deoxygenated DMF (approx. 1 mL per 200 mg of polymer) with an excess of U-SH thiol (2:1 molar ratio of thiol/alkene or 10:1 molar of thiol/alkyne) under argon atmosphere. Then, DMPA (5 mmol% relative to the alkene or alkyne groups) was added to the mixture. The reaction was magnetically stirred and carried out under argon atmosphere at rt inside of a chamber equipped with interior mirrors and illuminated by a Philips PL-S-9W UV Hg lamp with a maximum emission at 365 nm, for 6-8h, until complete functionalization was confirmed by ^{1}H NMR. The reaction mixture was precipitated into diethyl ether and then purified.

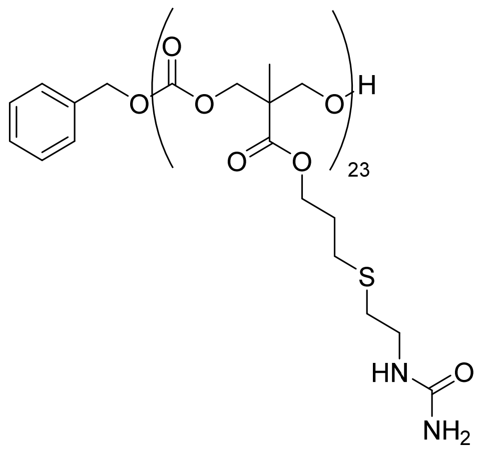

Synthesis and characterization of P(MACU)23. P(MACU)23 was synthesized following the aforementioned general procedure, employing the following reactants and solvents: P(MAC)23 (187.0 mg, 0.97 mmol of alkene groups), U-SH thiol (234.0 mg, 1.95 mmol), DMPA (12.5 mg, 0.05 mmol) and deoxygenated DMF (1 mL). The precipate was purified by washsing with DCM (3×10 mL) and Milli-Q® water (3×10 mL). Finally, the polymer was dried under vacuum to give P(MACU)23 as a vitreous solid. Yield: 47%. FTIR (KBr), (ν, cm^{-1}): 3464, 3371, 3218 ($N\text{-}H^{st}$), 2963 ($Csp^3\text{-}H^{st}$), 1753, 1655 ($C{=}O^{st}$), 1604 (NH_2^{δ}), 1549 ($N\text{-}H^{\delta}$). ^1H NMR (400 MHz, DMSO-d_6) δ (ppm): 7.41-7.34 (m, $C_6\mathbf{H_5}CH_2$), 6.06 (t, J = 5.2 Hz, N**H**), 5.50 (s broad, N$\mathbf{H_2}$), 5.14 (s, $C_6H_5C\mathbf{H_2}$), 5.06 (t, J = 5.5 Hz, O**H**), 4.35-4.08 (m, OC(O)OC$\mathbf{H_2}$, C(O)OC$\mathbf{H_2}$), 3.54-3.45 (m, C$\mathbf{H_2}$OH), 3.12 (q, J = 6.5 Hz, C$\mathbf{H_2}$NH), 2.57-2.50 (m, C$\mathbf{H_2}$SC$\mathbf{H_2}$), 1.82 (quint, J = 6.7 Hz, $CH_2C\mathbf{H_2}CH_2$), 1.22-1.15 (m, C$\mathbf{CH_3}$), 1.09 (s, C$\mathbf{CH_3}$).

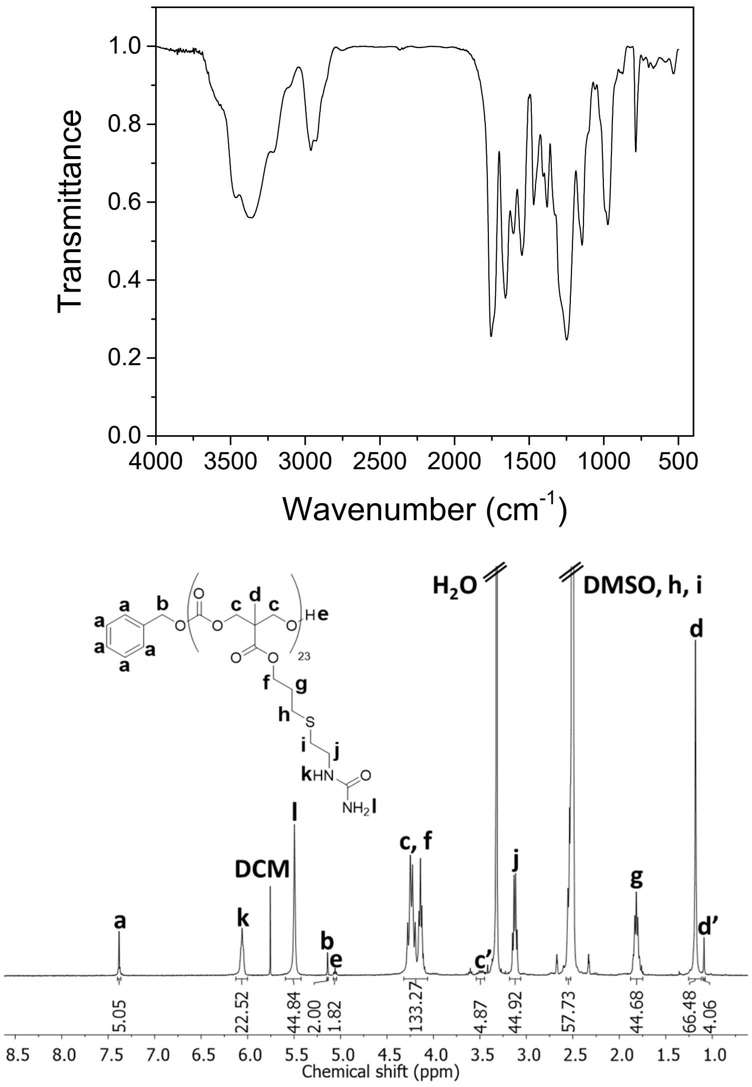

Figure S9. FTIR (top) and ^{1}H NMR (down) spectra of P(MACU)$_{23}$

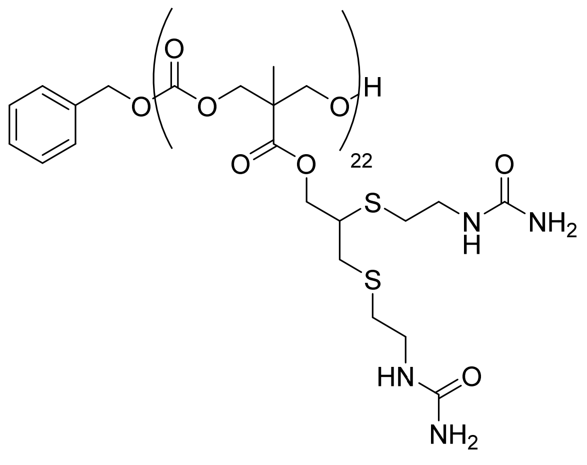

Synthesis and characterization of P(MPCU)$_{22}$. P(MPCU)$_{22}$ was synthesized following the aforementioned general procedure, employing the following reactants and solvents: P(MPC)$_{22}$ (200.0 mg, 0.98 mmol of alkyne groups), U-SH thiol (1.18 g, 9.824 mmol), DMPA (12.7 mg, 0.05 mmol) and deoxygenated DMF (1 mL). The precipate was purified by washing with DCM (3×10 mL) and Milli-Q® water (3×10 mL). Finally, the polymer was dried under vacuum to give P(MPCU)$_{22}$ as a white solid. Yield:

67%. FTIR (KBr), (ν, cm^{-1}): 3464, 3339, 3213 (N-H^{st}), 2930 (Csp^3-H^{st}), 1753, 1661 (C=O^{st}), 1609 (NH_2^{δ}), 1549 (N-H^{δ}). ^{1}H NMR (400 MHz, DMSO-d_6) δ (ppm): 7.41-7.36 (m, $C_6\mathbf{H_5}CH_2$), 6.29-6.00 (m, N**H**), 5.56 (s broad, N$\mathbf{H_2}$), 5.14 (s, $C_6H_5C\mathbf{H_2}$), 5.10 (t, *J* = 5.4 Hz, O**H**), 4.50-4.05 (m, OC(O)OC$\mathbf{H_2}$, C(O)OC$\mathbf{H_2}$), 3.58-3.43 (m, C$\mathbf{H_2}$OH), 3.22-3.07 (m, C$\mathbf{H_2}$NH, CH_2C**H**(CH_2)S), 2.87-2.68 (m, CHC$\mathbf{H_2}$S), 2.65-2.53 (m, SC$\mathbf{H_2}CH_2$), 1.22-1.16 (m, CC$\mathbf{H_3}$), 1.11 (s, CC$\mathbf{H_3}$).

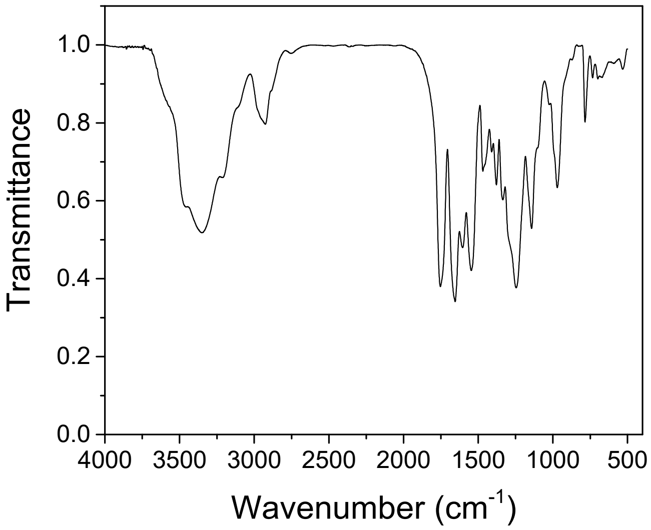

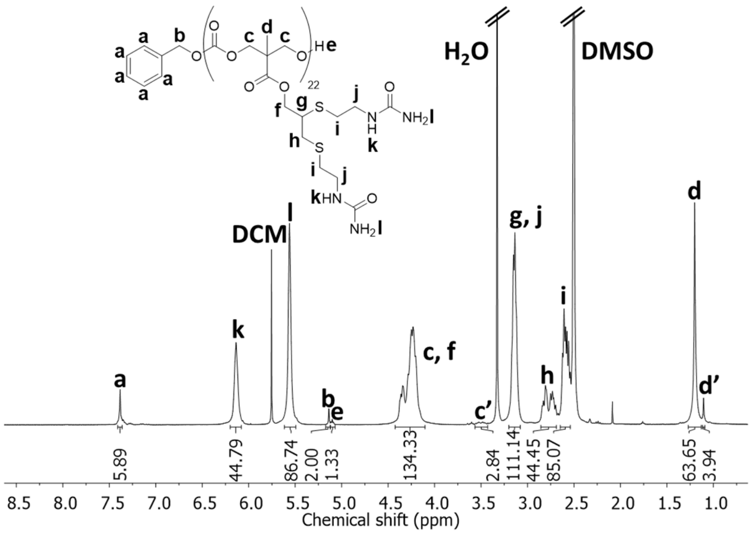

Figure S10. FTIR (top) and ^{1}H NMR (down) spectra of $P(MPCU)_{22}$

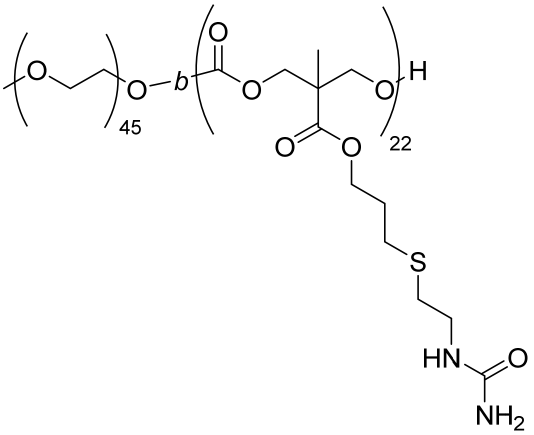

Synthesis and characterization of PEG_{45}-b-$P(MACU)_{22}$. PEG_{45}-*b*-$P(MACU)_{22}$ was synthesized following the aforementioned general procedure, employing the following reactants and solvents: PEG_{45}-*b*-$P(MAC)_{22}$ (150.0 mg, 0.51 mmol of alkene groups), U-SH thiol (123.5 mg, 1.03 mmol), DMPA (6.7 mg, 0.03 mmol) and deoxygenated DMF (1 mL) The precipate was purified by dialysis against Milli-Q® water (MWCO 1000 Da) for 48 h. Finally, the aqueous solution was freeze-dried to yield PEG_{45}-*b*-$P(MACU)_{22}$ as a viscous oil. Yield: 34%. FTIR (KBr), (ν, cm^{-1}): 3451, 3353, 3213 (N-H^{st}), 2921 (Csp^3-H^{st}), 1755, 1660 (C=O^{st}), 1608 (NH_2^{δ}), 1548 (N-H^{δ}). ^{1}H NMR (400 MHz, DMSO-d_6) δ (ppm): 6.08 (t, *J* = 6.0 Hz, N**H**), 5.51 (s, N**H₂**), 5.08 (t, *J* = 5.6 Hz, O**H**), 4.32-4.17 (m, OC(O)OC**H₂**), 4.14 (t, *J* = 6.0 Hz, C(O)OC**H₂**), 3.56-3.45 (m, OC**H₂**C**H₂**O), 3.24 (s, CH_2OC**H₃**), 3.12 (q, *J* = 6.8 Hz, C**H₂**NH), 2.58-2.50 (m, C**H₂**SC**H₂**), 1.81 (quint, *J* = 6.7 Hz, CH_2C**H₂**CH_2), 1.23-1.12 (m, CC**H₃**), 1.08 (s, CC**H₃**).

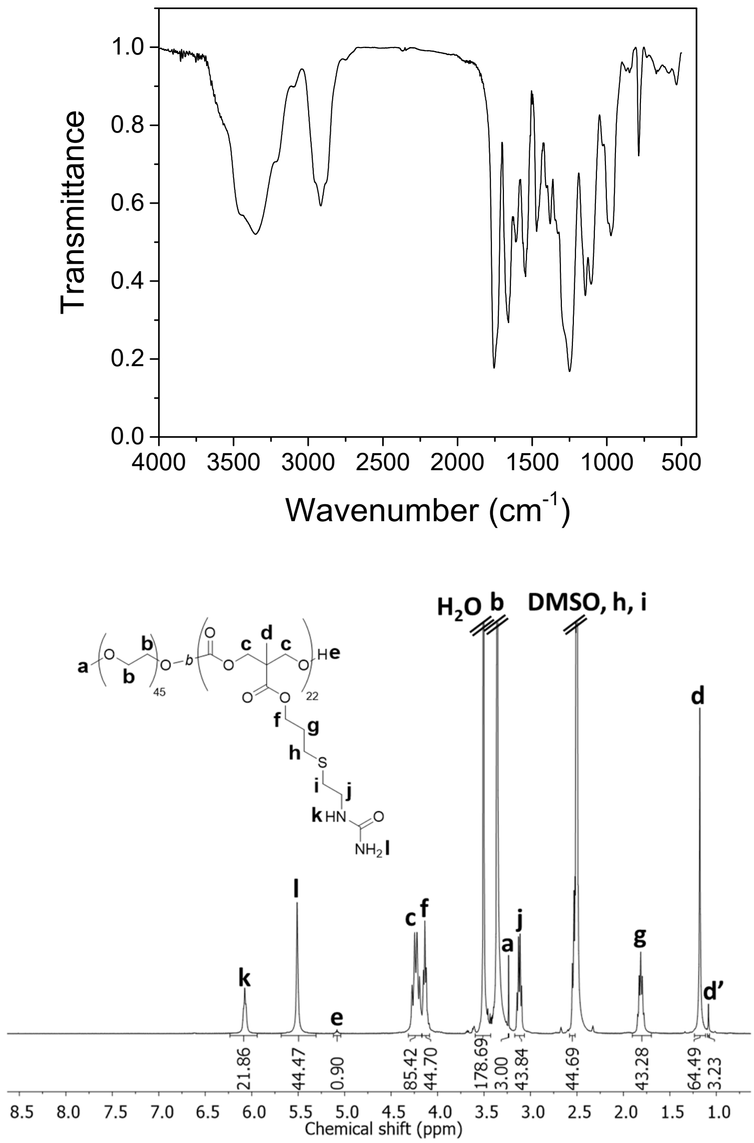

Figure S11. FTIR (top) and ^{1}H NMR (down) spectra of PEG$_{45}$-*b*-P(MACU)$_{22}$

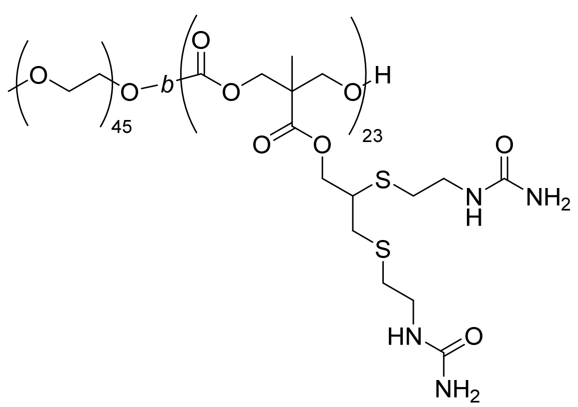

Synthesis and characterization of PEG$_{45}$-b-P(MPCU)$_{23}$. PEG$_{45}$-*b*-P(MPCU)$_{23}$ was synthesized following the aforementioned general procedure, employing the following reactants and solvents: PEG$_{45}$-*b*-P(MPC)$_{23}$ (150.0 mg, 0.51 mmol of alkyne groups), U-SH thiol (616.5 mg, 5.13 mmol), DMPA (6.6 mg, 0.03 mmol) and deoxygenated DMF (1 mL). Then, the precipate was purified by dialysis against Milli-Q® water (MWCO 1000 Da) for 48

h. Finally, the aqueous solution was freeze-dried to obtain PEG$_{45}$-*b*-P(MPCU)$_{23}$ as a colourless vitreous solid. Yield: 57%. FTIR (KBr), (ν, cm^{-1}): 3451, 3344, 3209 (N-Hst), 2916 (Csp^{3}-Hst), 1753, 1661 (C=Ost), 1605 (NH$_2$$^{\delta}$), 1549 (N-H$^{\delta}$). ^{1}H NMR (400 MHz, DMSO-d$_6$) δ (ppm): 6.15 (s, N**H**), 5.57 (s, N**H$_2$**), 5.11 (t, *J* = 5.4 Hz, O**H**), 4.49-4.04 (m, OC(O)OC**H$_2$**, C(O)OC**H$_2$**), 3.57-3.44 (m, OC**H$_2$**C**H$_2$**O), 3.24 (s, CH$_2$OC**H$_3$**), 3.21-3.04 (m, C**H$_2$**NH, CH$_2$C**H**(CH$_2$)S), 2.88-2.68 (m, CHC**H$_2$**S), 2.66-2.53 (m, SC**H$_2$**CH$_2$), 1.28-1.14 (m, CC**H$_3$**), 1.11 (s, CC**H$_3$**).

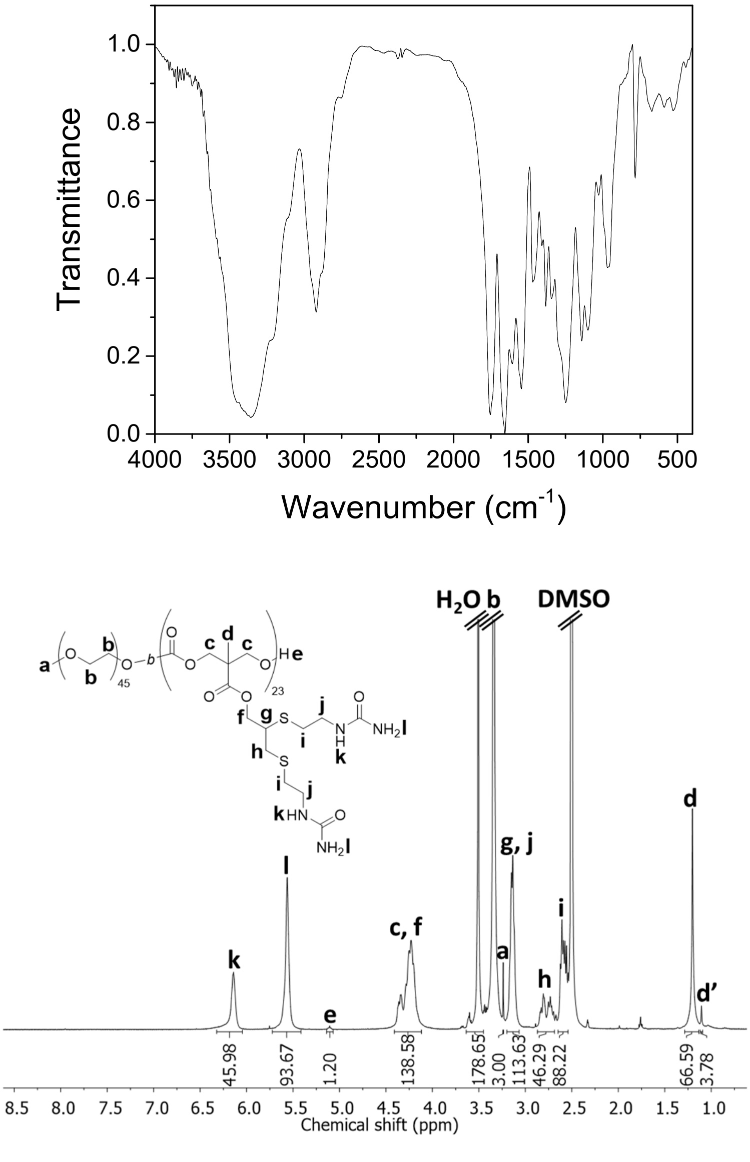

Figure S12. FTIR (top) and ^{1}H NMR (down) spectra of PEG$_{45}$-*b*-P(MPCU)$_{23}$

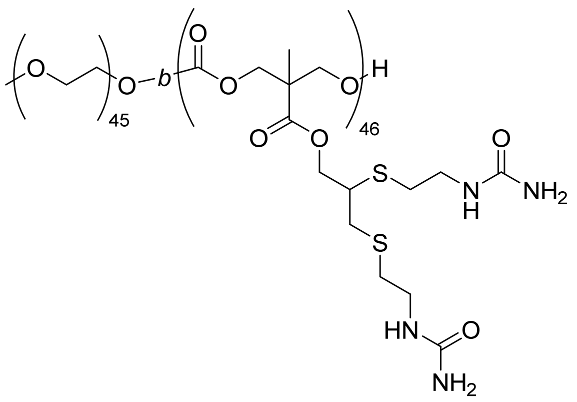

Synthesis and characterization of PEG$_{45}$-b-P(MPCU)$_{46}$. PEG$_{45}$-*b*-P(MPCU)$_{46}$ was synthesized following the aforementioned general procedure, employing the following reactants and solvents: PEG$_{45}$-*b*-P(MPC)$_{46}$ (200.0 mg, 0.82 mmol of alkyne groups), U-SH thiol (991.3 mg, 8.25 mmol), DMPA (10.6 mg, 0.04 mmol) and deoxygenated DMF (1 mL). The precipitate was purified by dialysis against Milli-Q® water (MWCO 1000 Da) for 48 h. Finally, the aqueous solution was freeze-dried to obtain PEG$_{45}$-*b*-P(MPCU)$_{46}$ as a white solid. Yield: 61%. FTIR (KBr), (ν, cm^{-1}): 3465, 3346, 3208 (N-Hst), 2920, 2878 (Csp^3-Hst), 1753, 1661 (C=Ost), 1604 (NH$_2$$^{\delta}$), 1547 (N-H$^{\delta}$). ^{1}H NMR (400 MHz, DMSO-d$_6$) δ (ppm): 6.16 (s, N**H**), 5.57 (s broad, N**H$_2$**), 5.11 (t, *J* = 5.2 Hz, O**H**), 4.44-4.08 (m, OC(O)OC**H$_2$**, C(O)OC**H$_2$**), 3.57-3.45 (m, OC**H$_2$**C**H$_2$**O), 3.24 (s, CH$_2$OC**H$_3$**), 3.21-3.08 (m, C**H$_2$**NH, CH$_2$C**H**(CH$_2$)S), 2.87-2.68 (m, CHC**H$_2$**S), 2.65-2.53 (m, SC**H$_2$**CH$_2$), 1.25-1.14 (m, CC**H$_3$**), 1.11 (s, CC**H$_3$**).

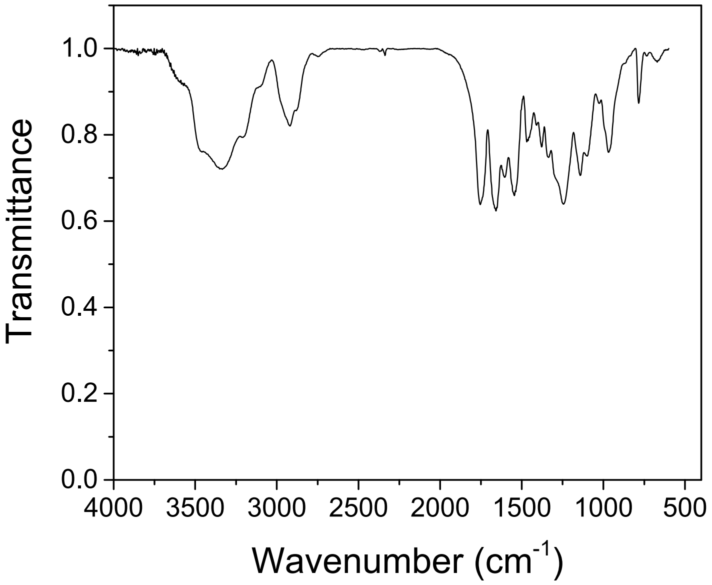

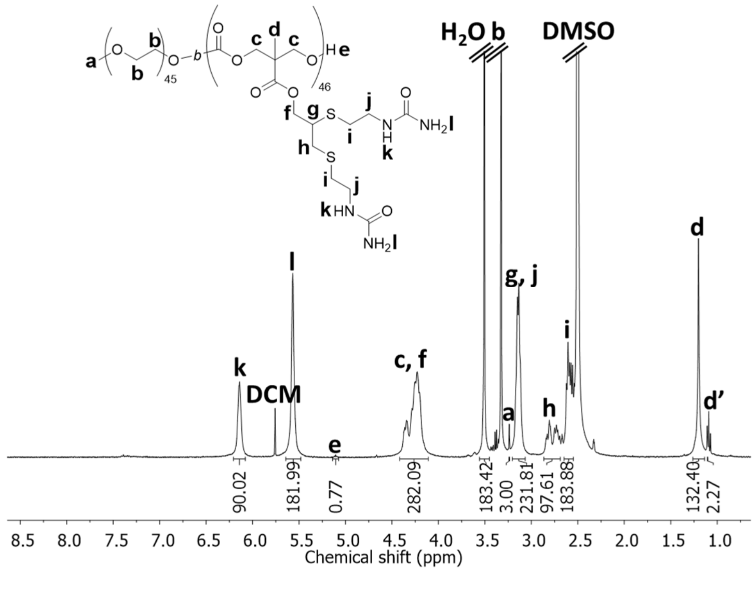

Figure S13. FTIR (top) and ^{1}H NMR (down) spectra of PEG$_{45}$-*b*-P(MPCU)$_{46}$

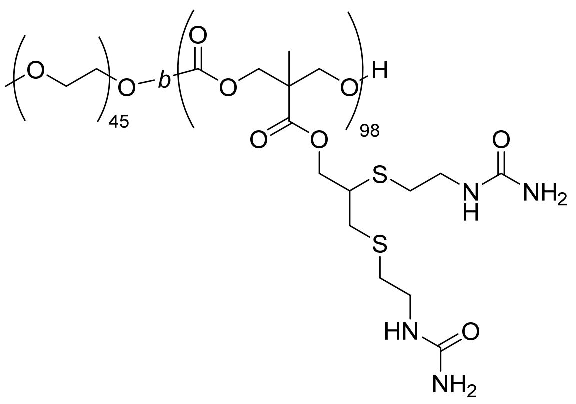

Synthesis and characterization of PEG$_{45}$-b-P(MPCU)$_{98}$. PEG$_{45}$-*b*-P(MPCU)$_{98}$ was synthesized following the aforementioned general procedure, employing the following reactants and solvents: PEG$_{45}$-*b*-P(MPC)$_{98}$ (200.0 mg, 0.92 mmol of alkyne groups), U-SH thiol (1.10 g, 9.15 mmol), DMPA (11.7 mg, 0.05

mmol) and deoxygenated DMF (1 mL),. The precipate was purified by dialysis against Milli-Q® water (MWCO 1000 Da) for 48 h. Finally, the aqueous solution was freeze-dried to obtain PEG_{45}-*b*-$P(MPCU)_{98}$ as a white solid. Yield: 66%. FTIR (KBr), (ν, cm^{-1}): 3469, 3336, 3208 (N-H^{st}), 2922, 2875 (Csp^3-H^{st}), 1755, 1661 (C=O^{st}), 1602 (NH_2^{δ}), 1545 (N-H^{δ}). ^{1}H NMR (400 MHz, DMSO-d_6) δ (ppm): 6.15 (s, N**H**), 5.57 (s broad, N**H₂**), 5.10 (t, *J* = 5.5 Hz, O**H**), 4.47-4.08 (m, OC(O)OC$\mathbf{H_2}$, C(O)OC$\mathbf{H_2}$), 3.54-3.48 (m, OC$\mathbf{H_2}$C$\mathbf{H_2}$O), 3.24 (s, CH_2OC$\mathbf{H_3}$), 3.22-3.05 (m, C$\mathbf{H_2}$NH, CH_2C**H**(CH_2)S), 2.87-2.68 (m, CHC$\mathbf{H_2}$S), 2.66-2.53 (m, SC$\mathbf{H_2}$$CH_2$), 1.25-1.15 (m, C$\mathbf{H_3}$), 1.11 (s, C$\mathbf{H_3}$).

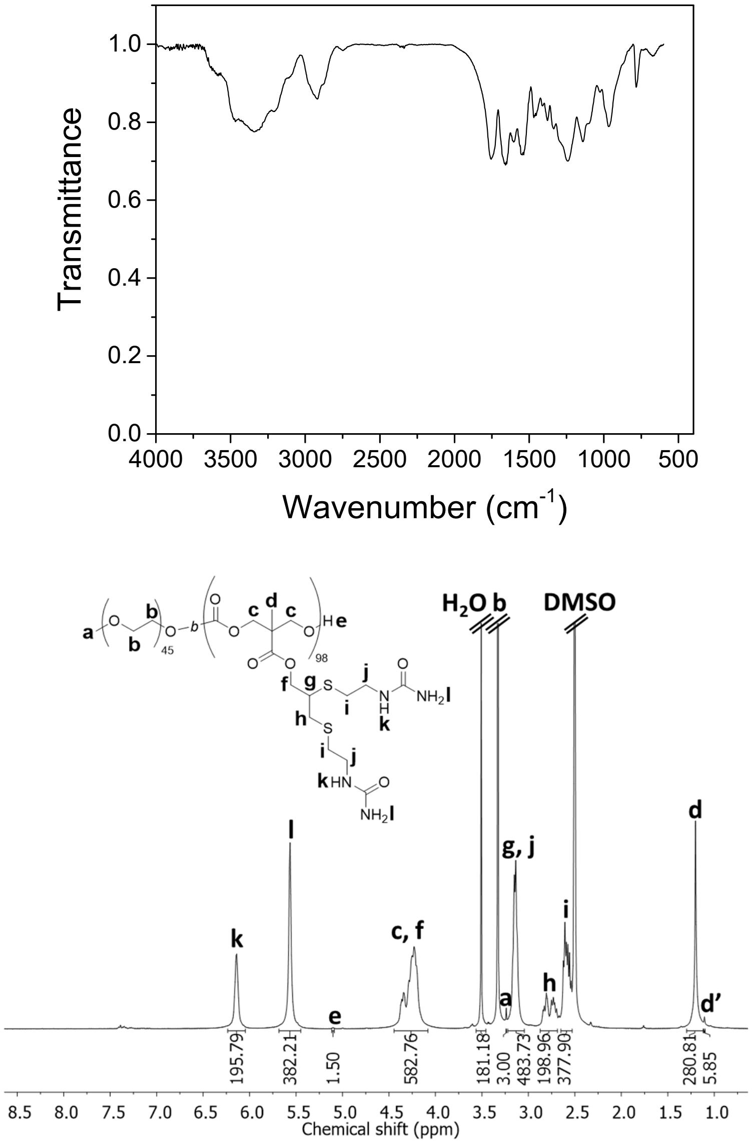

Figure S14. FTIR (top) and ^{1}H NMR (down) spectra of PEG$_{45}$-*b*-P(MPCU)$_{98}$

3.5. Synthesis and characterization of bis-MPA-U

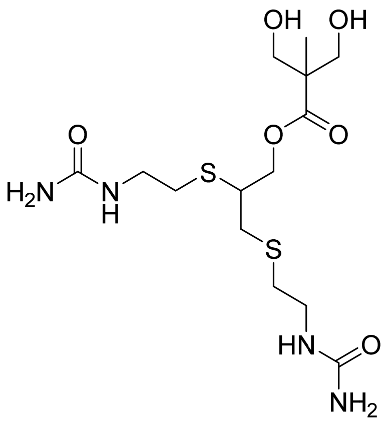

DMPA (2.2 mg, 0.01 mmol) was added to a solution of prop-2-yn-1-yl 3-hydroxy-2-(hydroxymethyl)-2-methylpropanoate (30.0 mg, 0.17 mmol) and U-SH thiol (102.6 mg, 0.85 mmol) in argon deoxygenated DMF (1 mL). The reaction was stirred at rt and irradiated with a Philips PL-S-9W UV Hg lamp, with a maximum emission at 365 nm, for 2 h. Then, the solution was concentrated under vacuum and the crude was purified by flash column chromatography on silica gel, eluting first with DCM/MeOH (85:15) and gradually increasing the polarity to DCM/MeOH (80:20). The bis-MPA-U product was isolated as a colourless viscous oil. Yield: 71%. FTIR (KBr), (ν, cm^{-1}): 3451, 3353 (O-H^{st}) (N-H^{st}), 2940 (Csp^3-H^{st}), 1720, 1654 (C=O^{st}), 1607 (NH_2^{δ}), 1552 (N-H^{δ}). ^{1}H NMR (400 MHz, DMSO-d_6) δ (ppm): 6.10 (t, *J* = 5.6 Hz, 2H, N**H**), 5.53 (s, 4H, N**H₂**), 4.70 (t, *J* = 5.5 Hz, 2H, O**H**), 4.27 (dd, *J* = 11.2, 4.9 Hz, 1H, C(O)OC**H**$_2$CH), 4.11 (dd, *J* = 11.1, 5.3 Hz, 1H, C(O)OC**H**$_2$CH), 3.51 (dd, *J* = 9.9, 5.4 Hz, 2H, CC**H**$_2$OH), 3.43 (dd, *J* = 10.5, 5.4 Hz, 2H, CC**H**$_2$OH), 3.16-3.06 (m, 5H, C**H**$_2$NH, CH_2C**H**(CH_2)S), 2.83-2.76 (m, 2H, CHC**H**$_2$S), 2.62 (t, *J* = 6.7 Hz, 2H, SC**H**$_2$$CH_2$), 2.58-2.53 (m, 2H, SC**H**$_2$$CH_2$), 1.06 (s, 3H, CC**H**$_3$). ^{13}C NMR (100 MHz, DMSO-d_6) δ (ppm): 174.4, 158.6, 158.5, 64.6, 63.9, 50.4, 44.0, 33.8, 32.6, 31.2, 16.9.

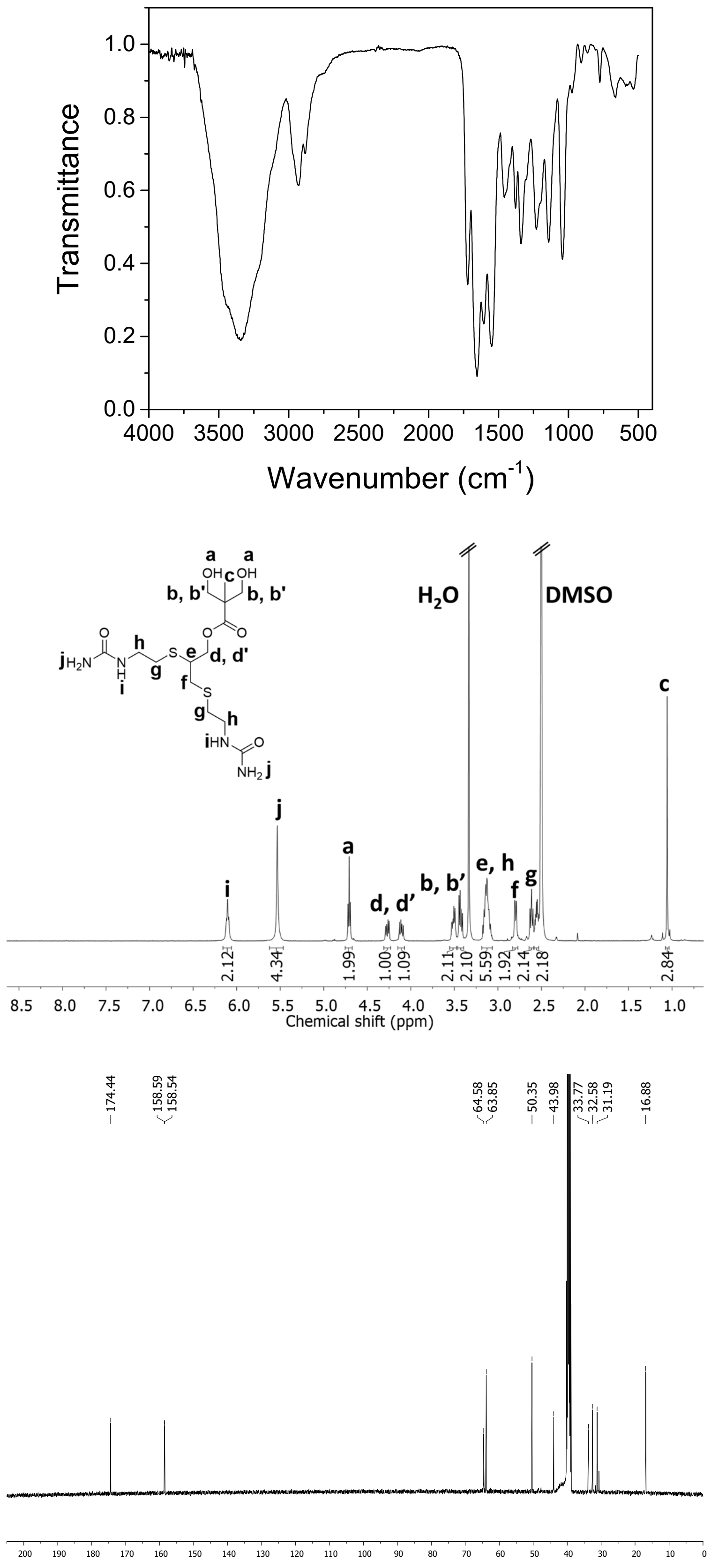

Figure S15. FTIR (top), ^{1}H NMR (middle) and ^{13}C NMR (bottom) spectra of Bis-MPA-U

4. Turbidity measurements

The optical transmittance at a 650 nm was recorded over three successive thermal cycles, by heating and then cooling from 20 to 90 ºC at a scanning rate of 1.0 ºC min^{-1}.

5. Variable temperature DLS measurements

Temperature-dependent DLS was conducted from 20 to 80 ºC at 5 ºC intervals with 5 min equilibration period between each measurement, beginning with the heating scan.

6. TEM images

For the preparation of the samples at rt, 10 μL of the BC suspension was deposited onto carbon-coated copper grid. After 30 s, the excess of water was removed by capillarity using a paper filter. Then, sample was stained adding 10 μL of uranyl acetate (1 wt %) solution in water onto the sample grid. After 30 s, the excess of staining agent was removed by capillarity using a paper filter. Finally, the grid was dried under vacuum overnight. For the preparation of the samples at 70 ºC, the carbon coated copper grid, the BC suspension and the staining agent, tungstic acid (3 wt %) solution in water, were heated in oven at 70 ºC for 30 min. Then, the BC dispersion was deposited onto grid at 70 ºC. The excess of water was removed by capillarity. Subsequently, tungstic acid solution was deposited onto the sample grid at 70 ºC. The excess of stain was removed by capillarity.

The TEM diameter was determined as the mean of 150 measurements histogram.

7. Variable temperature ^{1}H NMR experiments

BCs were dispersed in D_2O at a 5.0 mg mL^{-1} polymer concentration. The dispersion was heated and cooled from 20 to 80 ºC at 5 ºC intervals. Each measurement was preceded by a 5 min equilibration period.

8. Variable temperature SAXS experiments

For experiments performed upon heating and cooling ramps, a capillary containing the sample was inserted into a Linkam THM600 hot stage. SAXS patterns were acquired while the sample was heated from 20 to 80 °C at a controlled rate of 1.0 °C min^{-1} and the measurements were taken every 5 °C. The angular (q axis) was calibrated using standard samples of Silver Behenate. The scattering from water and that of the containing capillary was subtracted from the total scattering contribution.

9. Preparation of self-assemblies in water

Self-assembly of amphiphilic BCs by direct dispersion in water

BCs were dispersed in Milli-Q® water at the required concentration and sonicated in an ultrasonic bath (LC20H Ultrasonic) for 10 min.

Self-assembly of amphiphilic BCs by the co-solvent method

A solution of the BC (5.0 mg) in DMSO (1 mL) was prepared, and Milli-Q® water was gradually added while monitoring the absorbance of the solution at 650 nm using a UV-Vis spectrometer. Changes in absorbance served as an indirect determination of the loss of transmitted light resulting from the scattering of incident light due to the formation of self-assemblies. The addition of Milli-Q® water was concluded when absorbance values remained unchanged. Subsequently, the mixture was dialysed against Milli-Q® water using a Spectra Por® dialysis membrane (MWCO 1000 Da) for 3 days, with the water changed three times during the dialysis process.

Self-assembly of amphiphilic BCs by microfluidics

A BC solution of 5.0 mg mL^{-1} in DMSO (HPLC grade) was filtered through a 0.2 μm poly(tetrafluoroethylene) (PTFE) membrane. Then, this organic solution and Milli-Q® water were fed into a passive micromixer using two syringe pumps Harvard Apparatus PHD Ultra CP 4400, one for each solution. This micromixer, which has an inner volume of 8 μL, was designed for dividing the inlet streams into 15 channels of 40 μm each that were merged at the outlet, achieving instant mixing.[3] The mixing of solutions was carried out at an aqueous/organic solution phase ratio of 8:2 and residence time of 48 ms (10 mL min^{-1} of total flow rate). After the collection of self-assembly dispersions, the organic solvent was removed by dialysis against Milli-Q® water using a Spectra Por® dialysis membrane (MWCO 1000 Da) for 3 days, with the water changed three times during the dialysis process.

10. Determination of the Critical Aggregation Concentration (CAC)

CAC was determined by fluorescence spectroscopy using Nile Red as polarity sensitive probe. A solution of Nile Red in DCM at concentration of 6.0×10^{-6} M was prepared. Then, 100 μL of this solution was added to a vial, and the solvent evaporated at rt for 6 h. Subsequently, 600 μL of the self-assembly dispersion, with concentrations ranging from 1.0×10^{-4} to 1.0 mg mL^{-1}, were added into the vial, achieving a final Nile Red concentration of 1.0×10^{-6} M. The mixture

was then stirred overnight in orbital shaker. Finally, the emission spectra of Nile Red solution were registered from 530 to 900 nm while exciting at 550 nm.

11. Degradability studies of $P(MPCU)_{22}$ in water

$P(MPCU)_{22}$ (5.0 mg) was dispersed in Milli-Q® water (5 mL). Then, the polymeric suspension was heated to 80 ºC. At a specific time, the polymeric dispersion was cooled at rt and then was freeze-dried. The solid polymer was analysed by ^1H NMR spectroscopy in DMSO-d_6.

12. Preparation and quantification of Cur loaded into polymer micelles

A solution of BC (10.0 mg) and Cur (1.5 mg) in DMSO (2.0 mL) was prepared. Then, 5.0 mL of Milli-Q® water was gradually added to polymer solution for 1 h. Then, the dispersion was dialyzed against Milli-Q® water (MWCO 1000 Da) overnight. The concentration of polymer/Cur was approx. 1.0 mg, determined in triplicate by gravimetry. For it, three polymer/Cur aliquots (100 µL) were evaporated and weighed. The drug loading (DL) and encapsulation efficiency (EE) of Cur were determined by UV-Vis spectroscopy at 436 nm, using a standard calibration curve of Cur in DMSO. Three aliquots of polymer/Cur suspension (300 µL) were freeze-dried, and the resulting solid was dissolved in DMSO (2 mL) for analysis by UV-Vis spectroscopy.

The DL and EE were calculated by the following equations (1) and (2):

$$DL = \frac{mass\ of\ encapsulated\ Cur}{mass\ of\ polymer} \times 100\ (1)$$

$$EE = \frac{mass\ of\ encapsulated\ Cur}{mass\ of\ Cur\ in\ feed} \times 100\ (2)$$

Values for PEG_{45}-*b*-$P(MACU)_{22}$: DL = 9.5 ± 0.8%; EE = 63 ± 5%; Curcumin concentration = 114 ± 9 µg mL^{-1}.

Values for PEG_{45}-*b*-$P(MPCU)_{23}$: DL = 9.6 ± 0.1%; EE = 64 ± 0.6%; Curcumin concentration = 115 ± 1 µg mL^{-1}.

13. In vitro release of Cur from polymer self-assemblies

The release of Cur from BC self-assemblies was tested at 25 and 50 °C. The self-assemblies loaded with Cur were prepared by co-solvent method as explained in the above section. The dispersions were diluted at 0.1 mg mL^{-1} polymer concentration. The release of Cur was followed by UV-Vis spectroscopy, registering the evolution of absorbance at 436 nm with the time.

14. Supplementary figures and tables

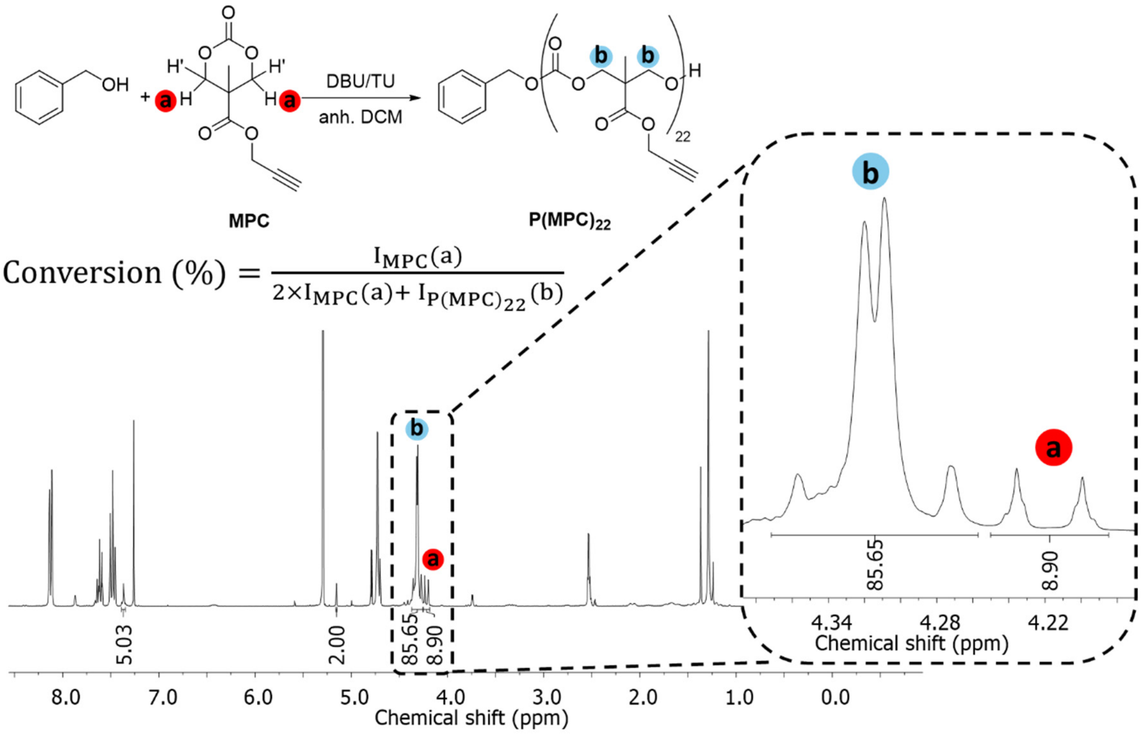

Figure S16. ^{1}H NMR (400 MHz, $CDCl_3$) spectra of $P(MPC)_{22}$ after two hours of polymerization to calculate experimental conversion (83 %) This monomer conversion was estimated by comparing the relative integration of the methylenic protons of the monomers at 4.21 ppm (OC(O)OC$\mathbf{H_2}$ labelled as *a*) with methylenic protons of polycarbonate backbone at 4.35-4.25 ppm (OC(O)OC$\mathbf{H_2}$ labelled as *b*)

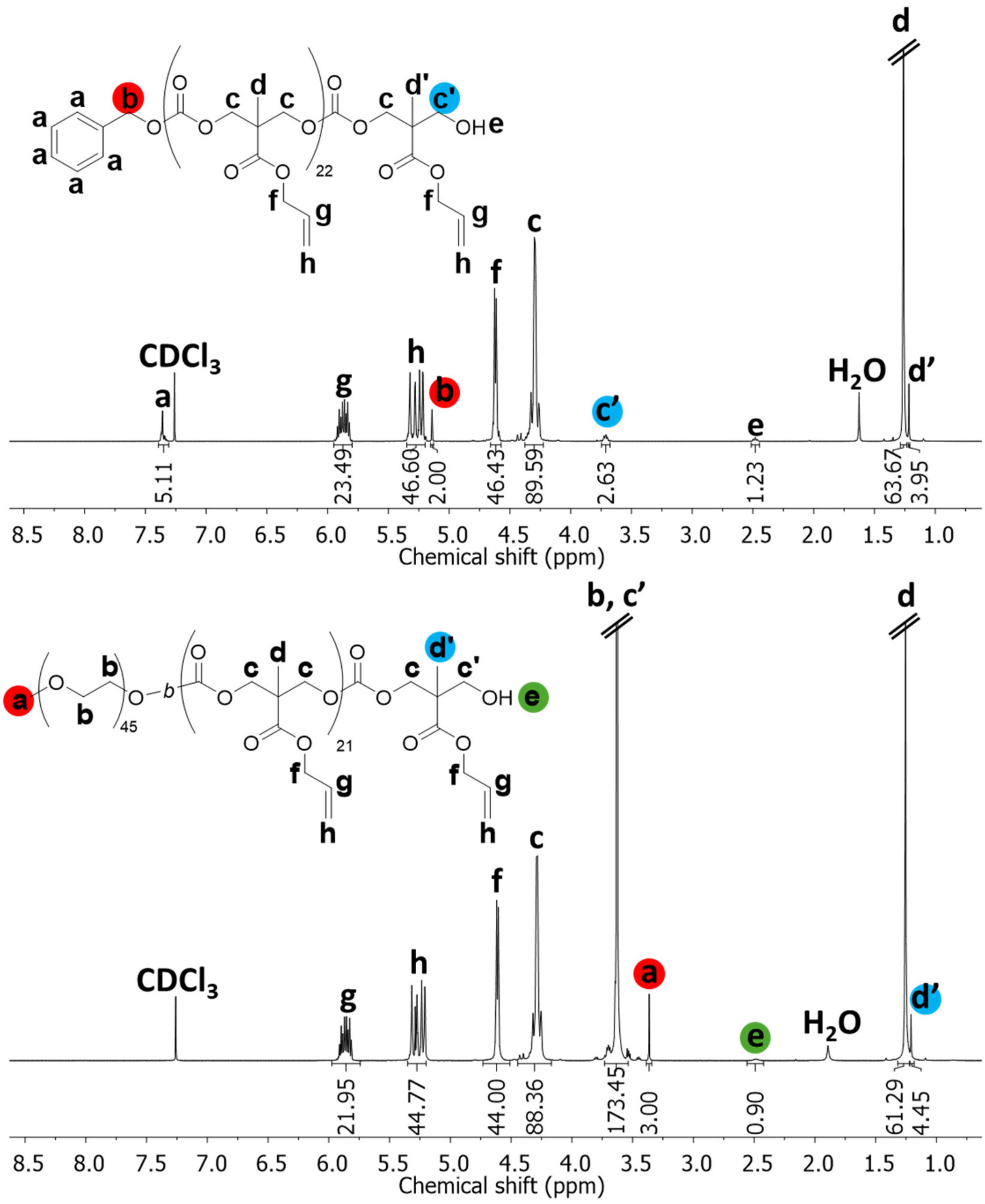

Figure S17. ^{1}H NMR (400 MHz, $CDCl_3$) spectra of $P(MAC)_{23}$ (top) and PEG_{45}-*b*-$P(MAC)_{22}$ (down) to verify the end-group fidelity. For the homopolycarbonates, the relative integration of ω-methylenic protons attached to hydroxyl end group at 3.72 ppm ($CC\mathbf{H_2}OH$ labelled as *c'*) and α-methylenic protons of the terminal benzyloxy group at 5.14 ppm ($C_6H_5C\mathbf{H_2}$ labelled as *b*) was about 1:1 ratio. In the case of BCs, a well end-group fidelity was also confirmed by the comparison between the integration of the α-terminal methoxy protons of the PEG segment at 3.37 ppm ($CH_2OC\mathbf{H_3}$ labelled as *a*) and the integration of the either ω-terminal hydroxyl protons at 2.49 ppm (**HO** labelled as *e*) or methyl protons at 1.21 ppm ($C\mathbf{CH_3}$ labelled as *d'*). Values match with the expected values for a controlled polymerization

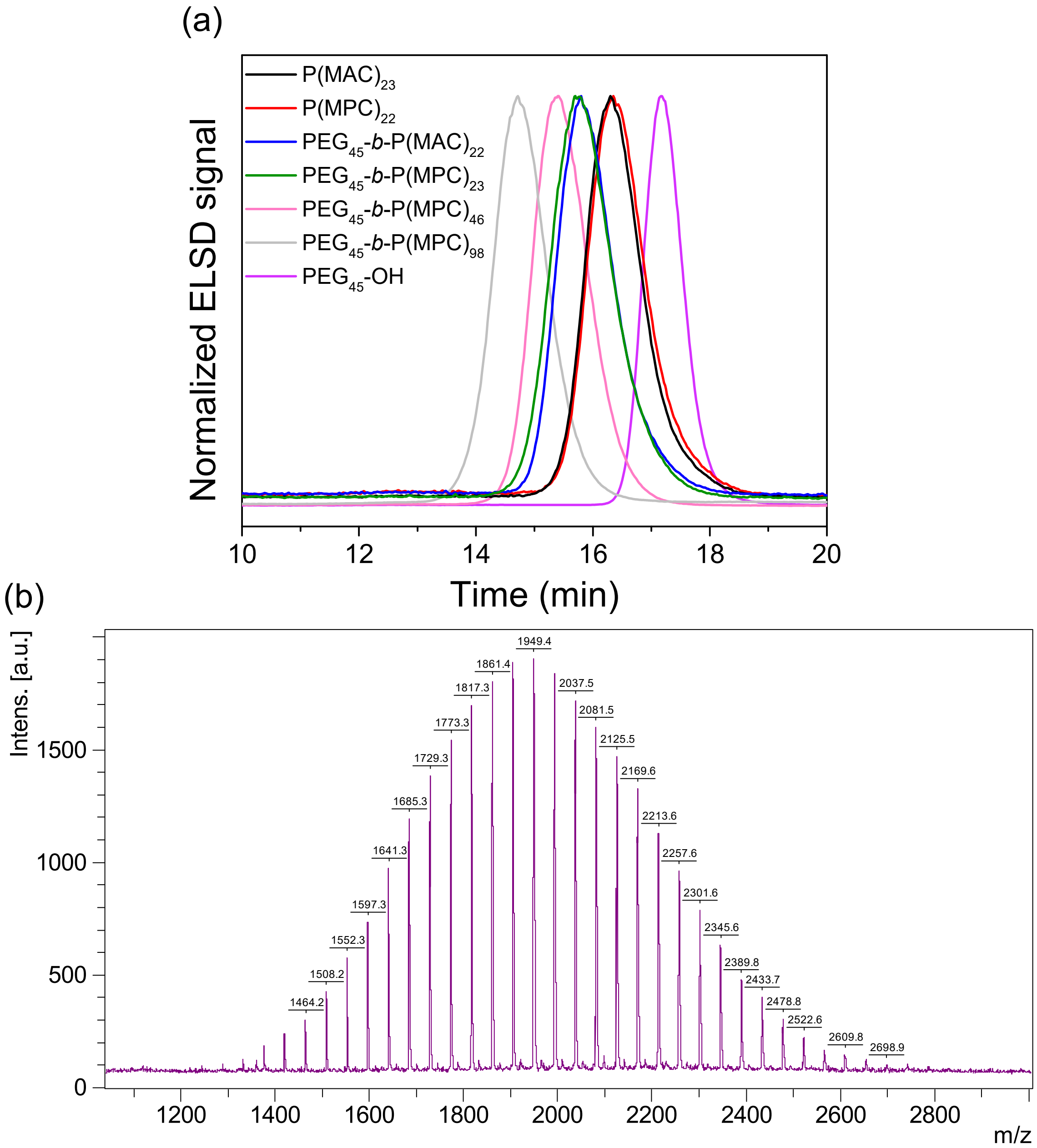

Figure S18. (a) SEC curves of homopolycarbonates and amphiphilic BCs (see Table 1 in the main text). **(b)** MALDI-TOF mass spectrum of PEG$_{45}$-OH polymer

14.1. MALDI-TOF analysis of homopolycarbonate and BC precursors

The MALDI-TOF mass spectra of homopolycarbonates exhibited a single mass distribution, which was consistent with the initiation by benzyl alcohol. Only when the polymerization was performed at higher monomer concentrations and for longer durations, a second distribution corresponding to the polycarbonate with both hydroxyl-terminated ends appeared due to a possible ROP initiation by residual water or monomer, or, more likely from back-biting and

transesterification side reactions (Figure S19).[4,5] However, detailed characterization of the resultant BCs by MALDI-TOF mass spectroscopy was not possible because, as the molar mass increases, the resolution and accuracy of the mass spectrometer decreases.[6] Therefore, well-resolved mass spectra featuring individual macromolecular species were not obtained; instead, broad mass distributions were observed.

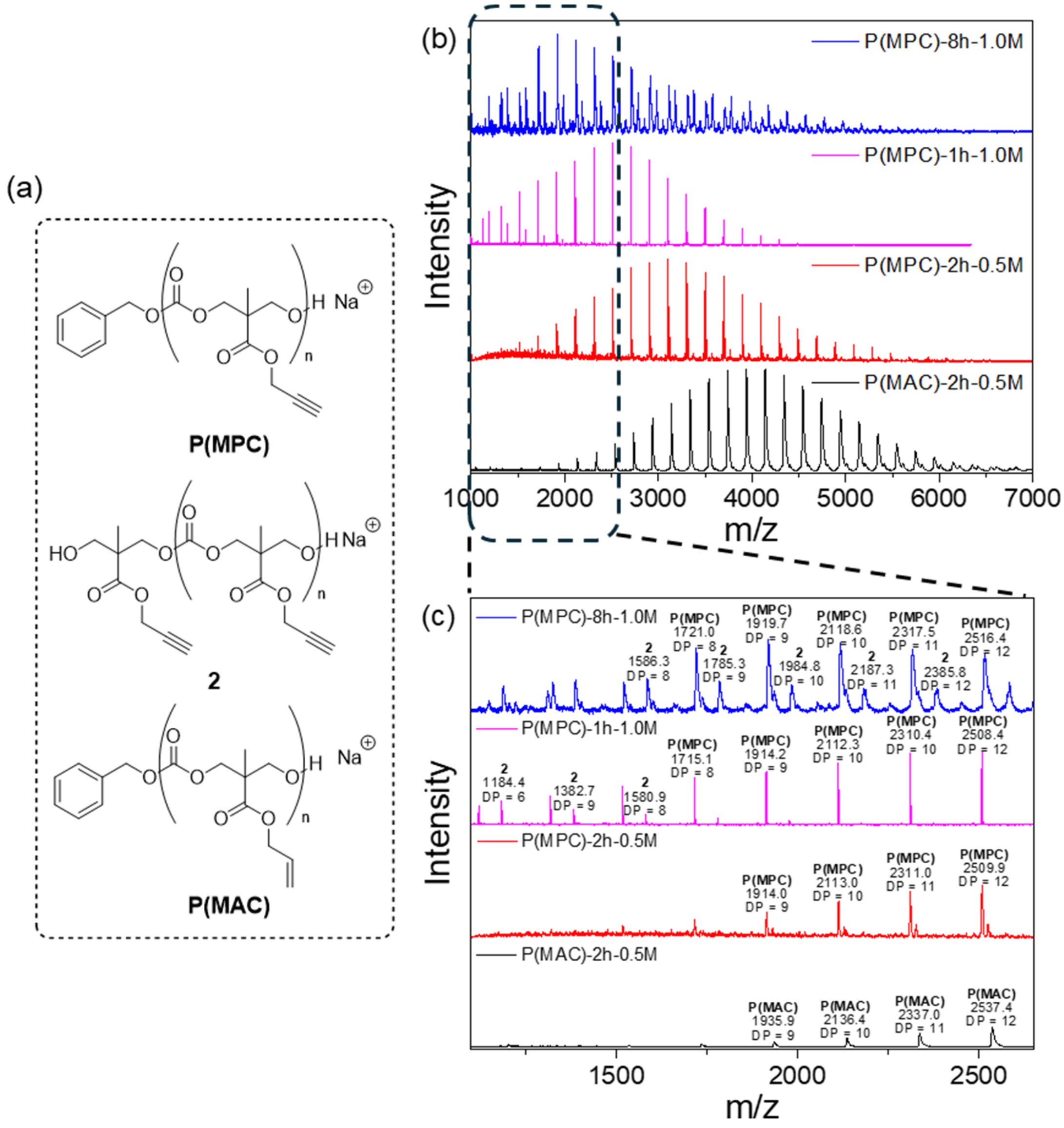

Figure S19. **(a)** Chemical structure of the target P(MAC)n and P(MPC)n and by-product detected in the ROP polymerization of MPC. **(b)** MALDI-TOF mass spectra analysis of ROP of MPC at 1.0 M, 35 ºC for 8 h, ROP of MPC at 1.0 M, 35 ºC for 1 h and ca. 80% conversion, ROP of MPC at 0.5 M, 35 ºC for 2 h and ca. 80% conversion and ROP of MAC at 0.5 M, 35 °C for 2 h and ca. 80% conversion (from 1000 to 7000 m/z range). **(c)** Expanded views of macromolecular species of DPs from 6 to 12 (from 1100 to 2650 m/z range). Matrix employed trans-2-[3-(4-tert-butylphenyl)-2-methyl-2-propenylidene]malononitrile (DCTB)

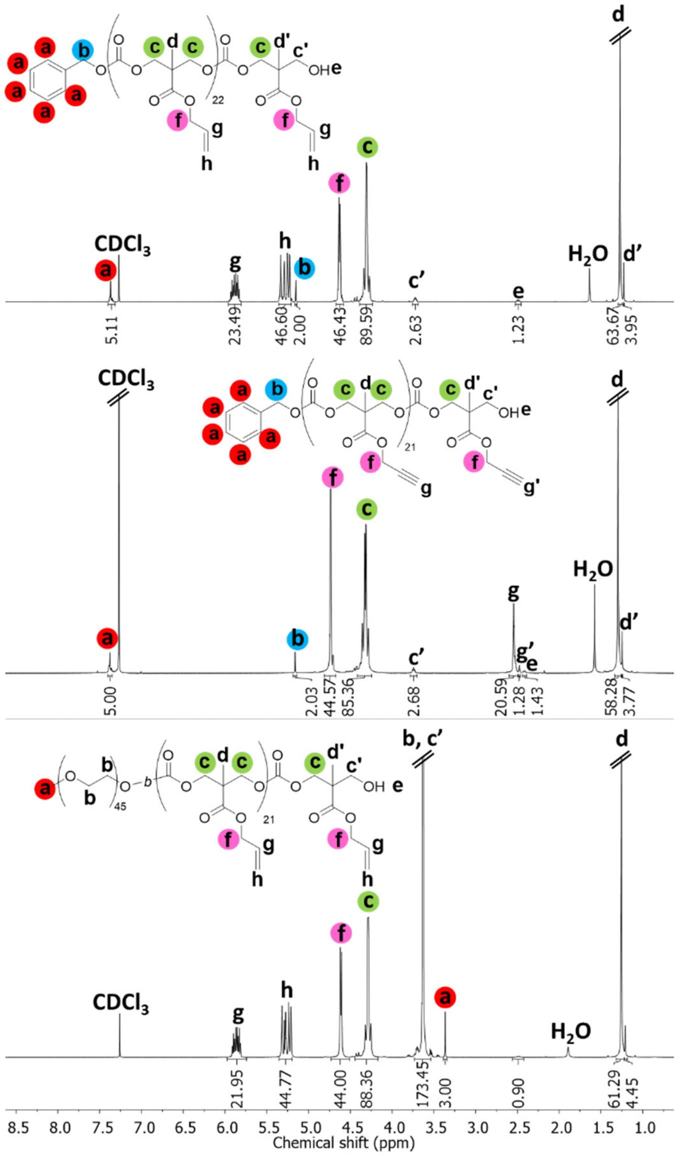

Figure S20. ^{1}H NMR (400 MHz, $CDCl_3$) spectra of $P(MAC)_{23}$ (top), $P(MPC)_{22}$ (middle) and PEG_{45}-*b*-$P(MAC)_{22}$ (down) to determine the DP. Average DP of homopolycarbonates was estimated from the relative intensities of signals corresponding to the α-terminal chain end coming from the benzylic alcohol initiator species (aromatic protons labelled as *a* at 7.38-7.32 ppm or methylenic protons labelled as *b* at 5.14 ppm) with those from methylenic protons of the side groups labelled as *f* at 4.62 ppm for $P(MAC)_n$ and at 4.66 ppm for $P(MPC)_n$ or those from the polycarbonate main chain labelled as *c* at 4.35-4.25 ppm. The estimated values were 23 for $P(MAC)_n$ and 22 for $P(MPC)_n$. Therefore, polymers were denoted as $P(MAC)_{23}$ and $P(MPC)_{22}$. In the case of BCs, the DP^{NMR} was determined from the relative integration of resonances corresponding to the α-terminal methoxy protons of the PEG segment labelled as *a*

at 3.37 ppm against those of the methylenic protons of the polycarbonate backbone labelled as *c* at 4.35-4.22 ppm.

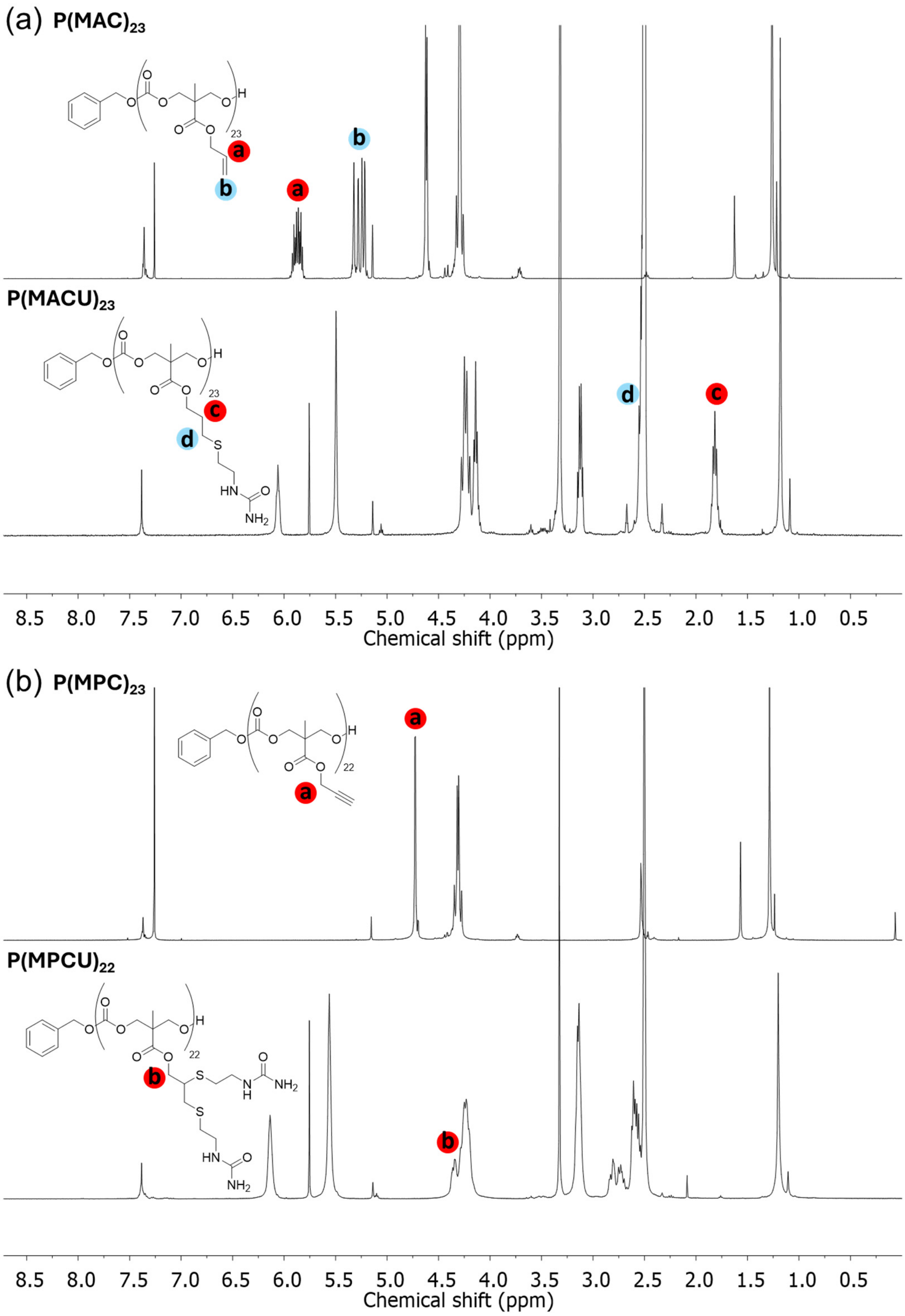

Figure S21. (a) ^{1}H NMR (400 MHz, $CDCl_3$) spectra of $P(MAC)_{23}$ (top), and ^{1}H NMR (400 MHz, DMSO-d_6) spectra of $P(MACU)_{23}$ (down) and **(b)** ^{1}H NMR (400 MHz, $CDCl_3$) spectra of $P(MPC)_{22}$ (top), and ^{1}H NMR (400 MHz, DMSO-d_6) spectra of $P(MPCU)_{22}$ (down)

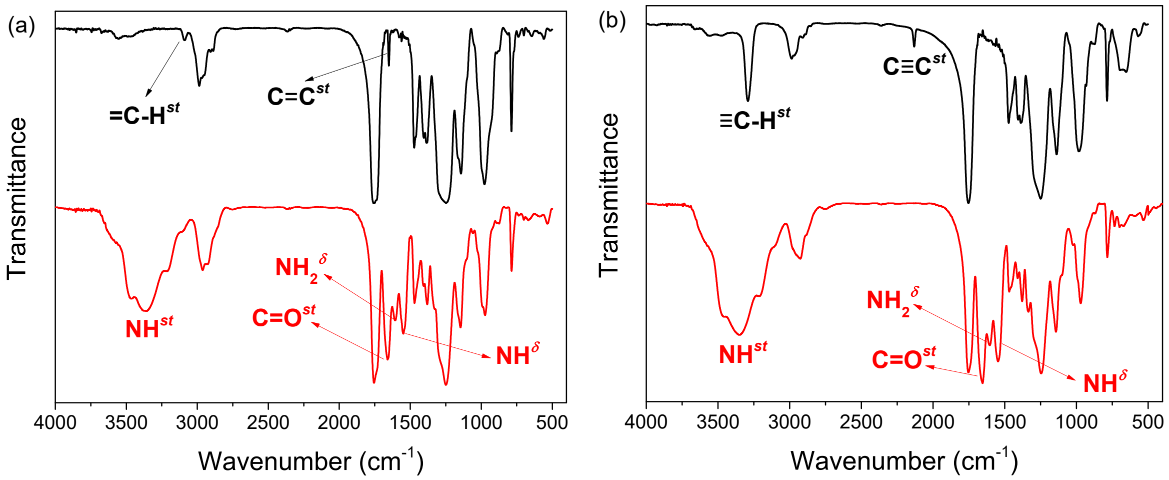

Figure S22. FTIR spectra of **(a)** P(MAC)$_{23}$ (top) and P(MACU)$_{23}$ (down) and **(b)** P(MPC)$_{22}$ (top) and P(MPCU)$_{22}$ (down)

Table S2. Thermal properties of homopolycarbonates and BCs

		1st Heating scan		2nd Heating scan
Polymer	TGA [°C] [a]	T_g [°C] [b]	T_m [°C] (ΔH_m (J g^{-1})) [c]	T_g [°C] [b]
P(MACU)$_{23}$	193	19	73 (1.2)	17
P(MPCU)$_{22}$	187	43	96 (0.4)	40
PEG$_{45}$-*b*-P(MACU)$_{22}$	192	–8	-	–7
PEG$_{45}$-*b*-P(MPCU)$_{23}$	191	21	-	19
PEG$_{45}$-*b*-P(MPCU)$_{46}$	189	33	-	32
PEG$_{45}$-*b*-P(MPCU)$_{98}$	189	40	-	42

[a] Decomposition temperature associated to mass loss determined by TGA as the onset point in the weight loss curve recorded at 10 ºC min^{-1} heating rate (nitrogen atmosphere). [b] Glass transition temperature (T_g) determined by DSC at the half height of baseline jump during the corresponding heating scan. [c] Melting temperature (T_m) given at the maximum of the corresponding peak and associated enthalpy (ΔH_m) determined by DSC during the first heating scan at 10 ºC min^{-1} rate. DSC scans from -50 ºC to 130 ºC at 10 ºC min^{-1}.

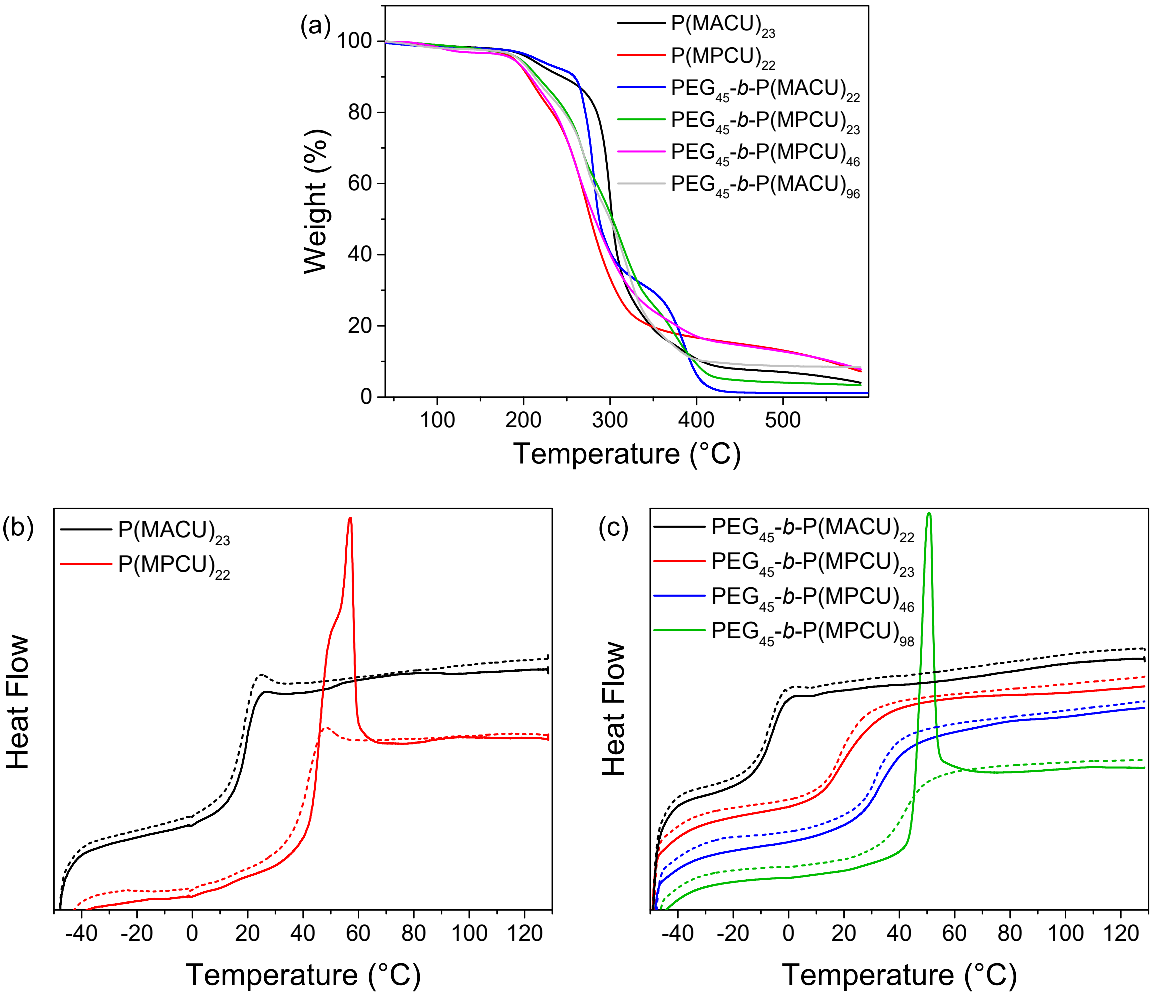

Figure S23. (a) TGA curves of polymers registered at 10 ºC min^{-1} heating rate under nitrogen atmosphere. DSC curves of **(b)** homopolycarbonates and **(c)** BCs corresponding to the first heating scan (solid line) and second heating scan (dashed line) registered at a 10 ºC min^{-1} rate (exo down)

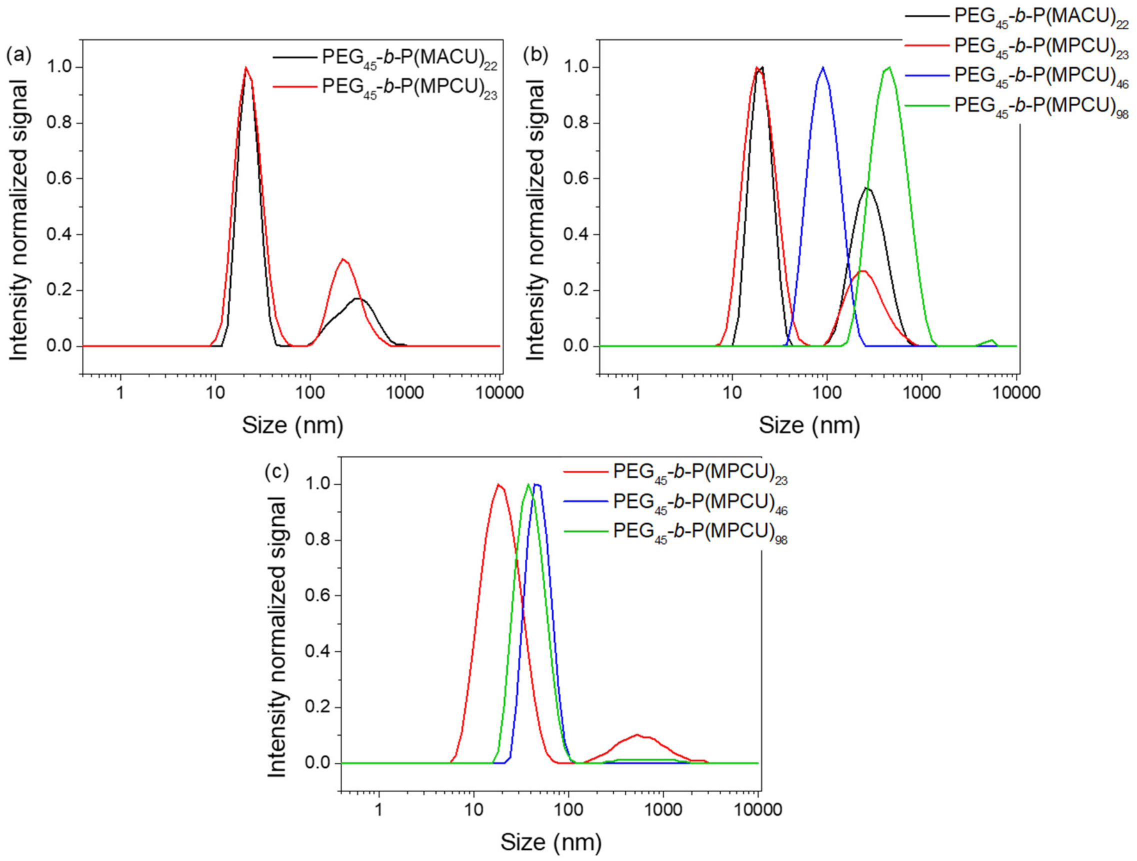

Figure S24. DLS intensity size distributions at 25 ºC of BC self-assemblies in water prepared by **(a)** direct dispersion, **(b)** co-solvent method and **(c)** microfluidics at a concentration of 1.0 mg mL^{-1}

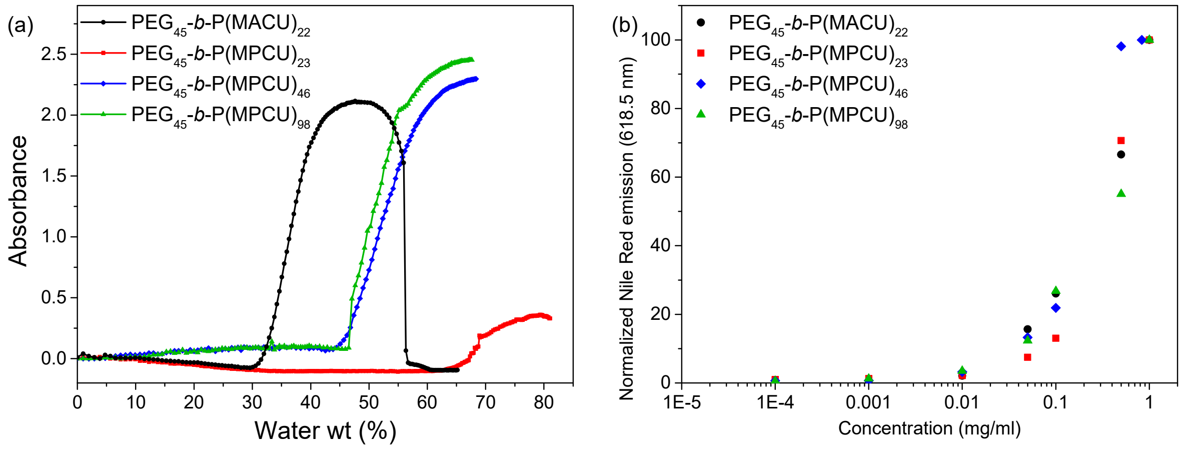

Figure S25. (a) Turbidity plot registered as the absorbance modification at 650 nm upon self-assembly when water was slowly added to a 5 mg mL^{-1} DMSO solution of the amphihilic BCs. **(b)** Normalized fluorescence emission of Nile Red at 618.5 nm (λ_{exc} = 550 nm) vs amphiphilic BC concentration

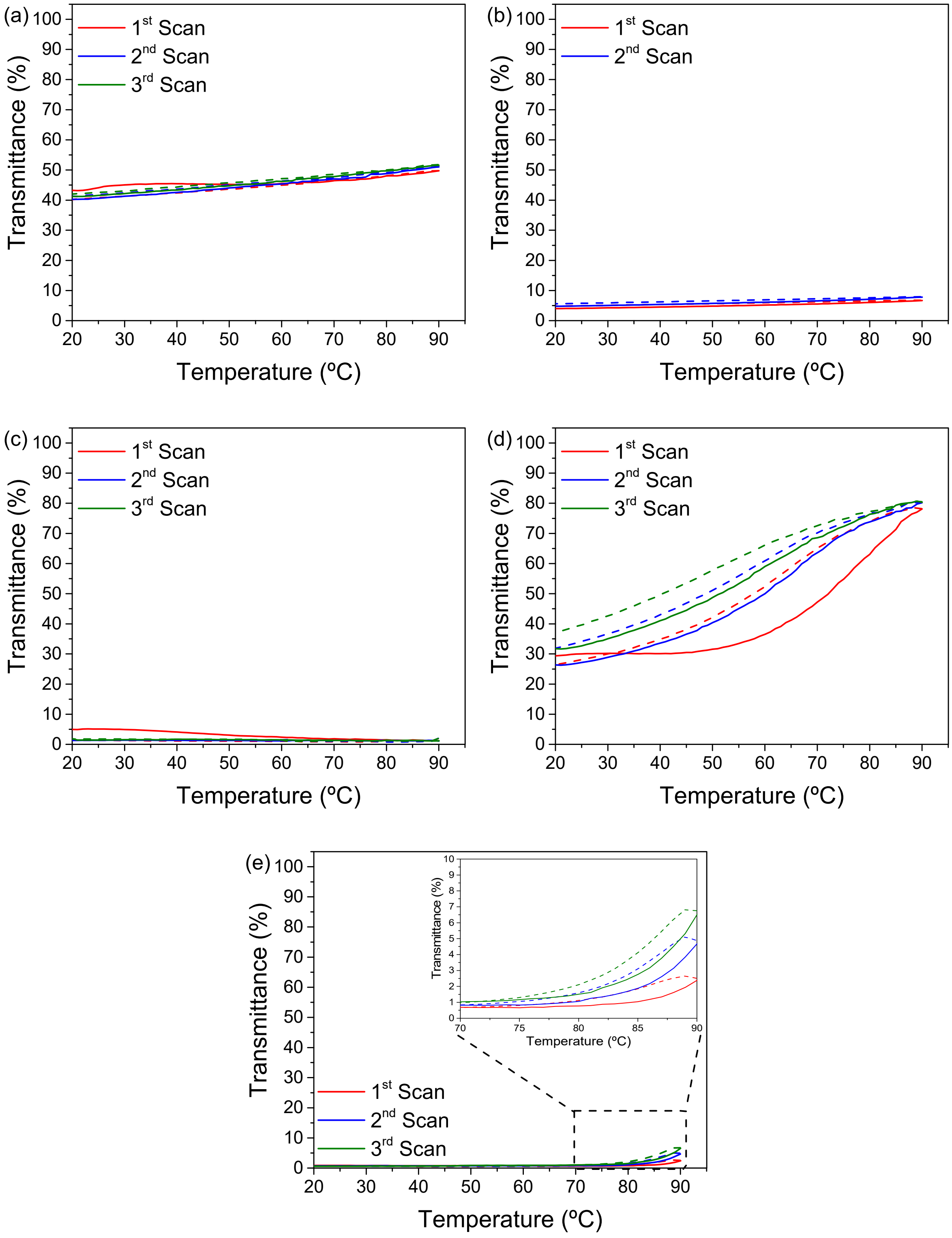

Figure S26. Temperature-dependent transmittance curves of $P(MACU)_{23}$ in water at **(a)** 0.1, **(b)** 0.3 and **(c)** 1.0 mg mL^{-1} polymer concentration and temperature-dependent transmittance curves of $P(MPCU)_{22}$ in water at **(d)** 0.1 and **(e)** 1.0 mg mL^{-1} polymer concentration upon repeated heating (solid line) and cooling (dashed line) at a scanning rate of 1.0 °C min^{-1}

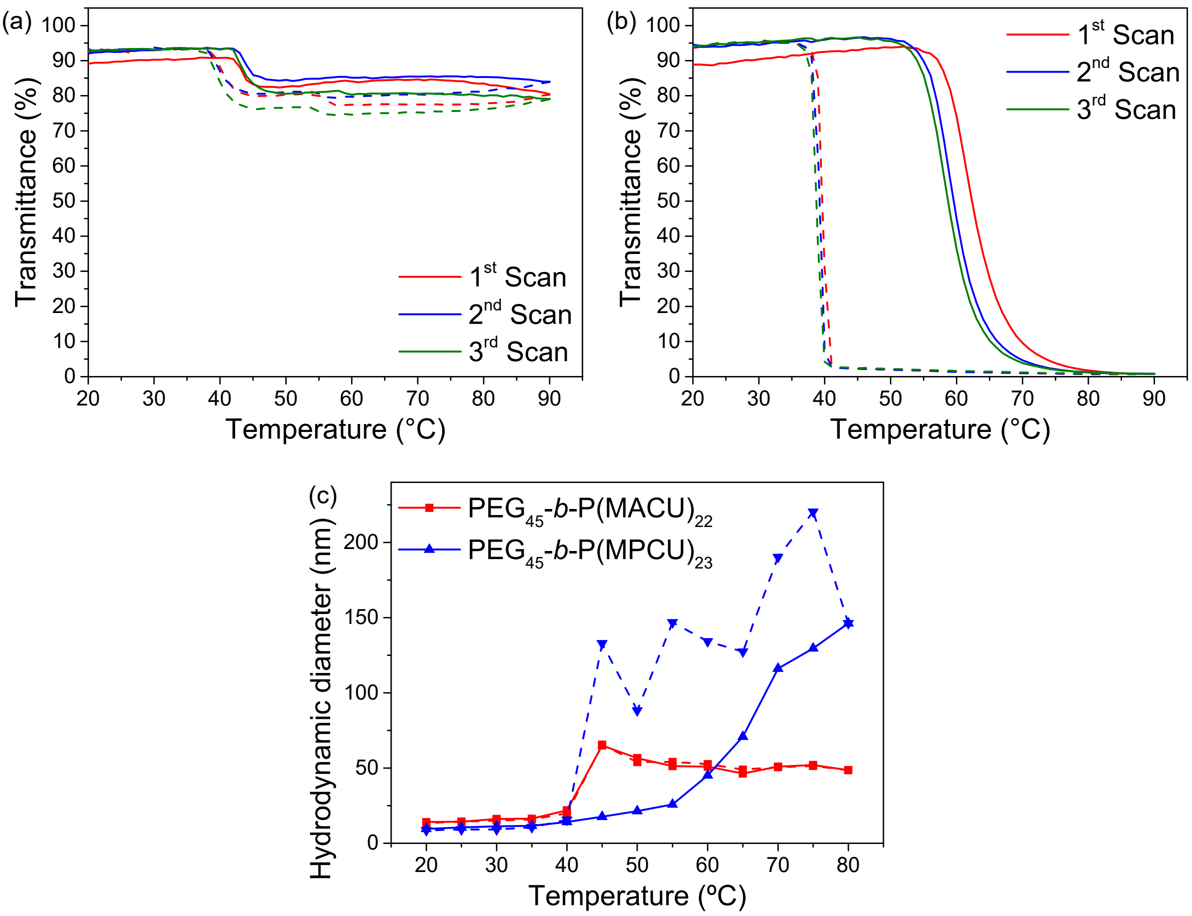

Figure S27. Temperature-dependent transmittance curves of **(a)** PEG_{45}-*b*-$P(MACU)_{22}$ and **(b)** PEG_{45}-*b*-$P(MPCU)_{23}$ dispersions at 5.0 mg mL^{-1} polymer concentration upon three consecutive heating (solid line) and cooling (dashed line) at a scanning rate of 1.0 °C min^{-1}. **(c)** Temperature evolution of number average D_h for PEG_{45}-*b*-$P(MACU)_{22}$ and PEG_{45}-*b*-$P(MPCU)_{23}$ micelles upon heating (solid line) and cooling (dashed line), with temperature intervals of 5 °C and equilibrating time of 5 min before measuring

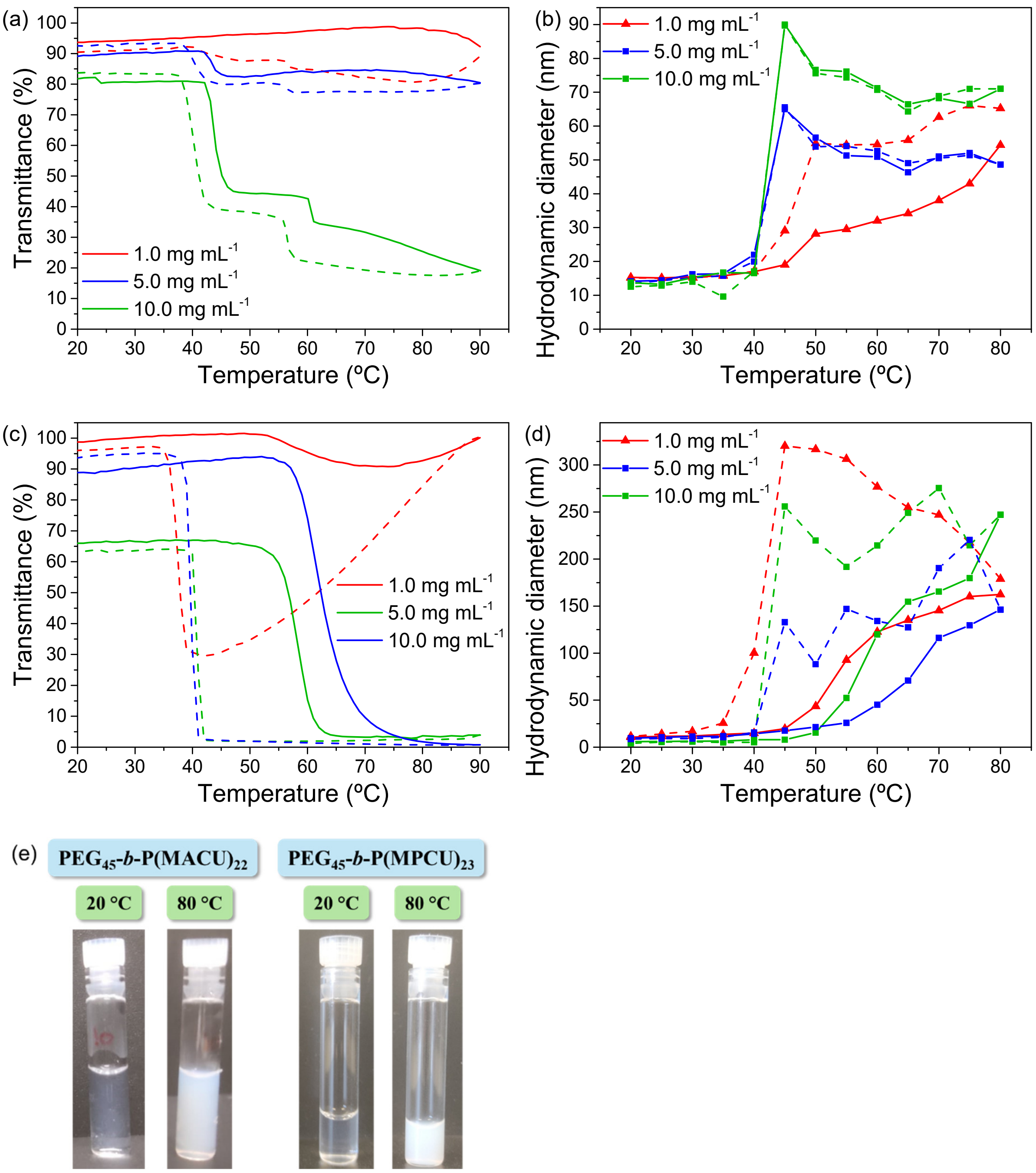

Figure S28. (a) Temperature-dependent transmittance curves and **(b)** temperature evolution of number average D_h for PEG$_{45}$-*b*-P(MACU)$_{22}$ dispersions. **(c)** Temperature-dependent transmittance curves and **(d)** temperature evolution of number D_h for PEG$_{45}$-*b*-P(MPCU)$_{23}$ dispersions. Solid line: heating; dashed line: cooling. **(e)** Photographs of PEG$_{45}$-*b*-P(MACU)$_{22}$ and PEG$_{45}$-*b*-P(MPCU)$_{23}$ dispersions at polymer concentration of 10.0 mg mL^{-1}, taken at temperatures of 20 and 80 °C

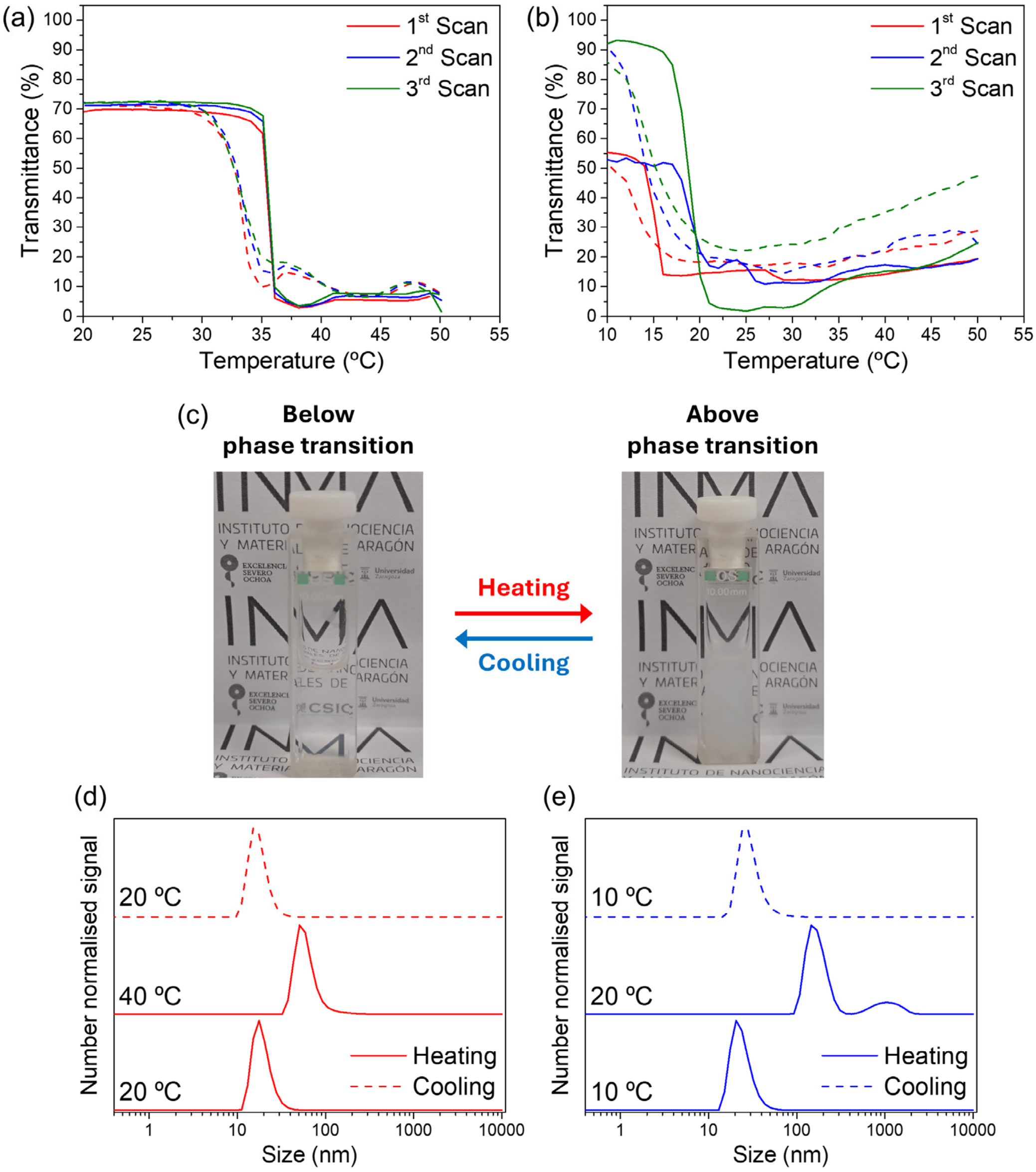

Figure S29. Temperature-dependent transmittance curves of **(a)** PEG_{45}-*b*-P(MACU)_{22} and **(b)** PEG_{45}-*b*-P(MPCU)_{23} dispersions in PBS at 5.0 mg mL^{-1} polymer concentration upon three consecutive heating (solid line) and cooling (dashed line) at a scanning rate of 1.0 °C min^{-1}. **(c)** Photographs of PEG_{45}-*b*-P(MPCU)_{23} dispersions in PBS (pH = 7.4) at polymer concentration of 5.0 mg mL^{-1}, taken at below and above transition temperature. DLS number size distributions of **(d)** PEG_{45}-*b*-P(MACU)_{22} and **(e)** PEG_{45}-*b*-P(MPCU)_{23} at 5.0 mg mL^{-1} concentration in PBS

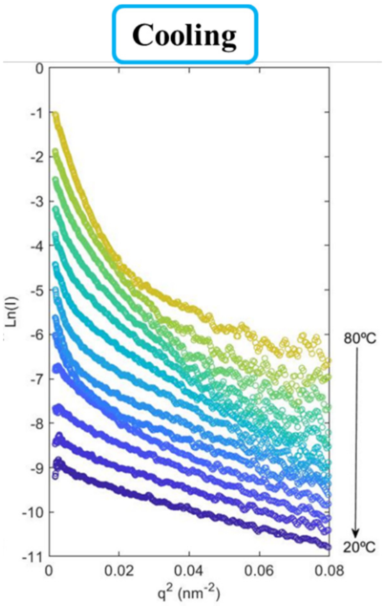

Figure S30. Guinier plots of the scattering from the PEG_{45}-*b*-$P(MPCU)_{23}$ dispersion at 1.0 mg mL^{-1} upon cooling at a scanning rate of 1.0 °C min^{-1}. Sample prepared by co-solvent method

(a)

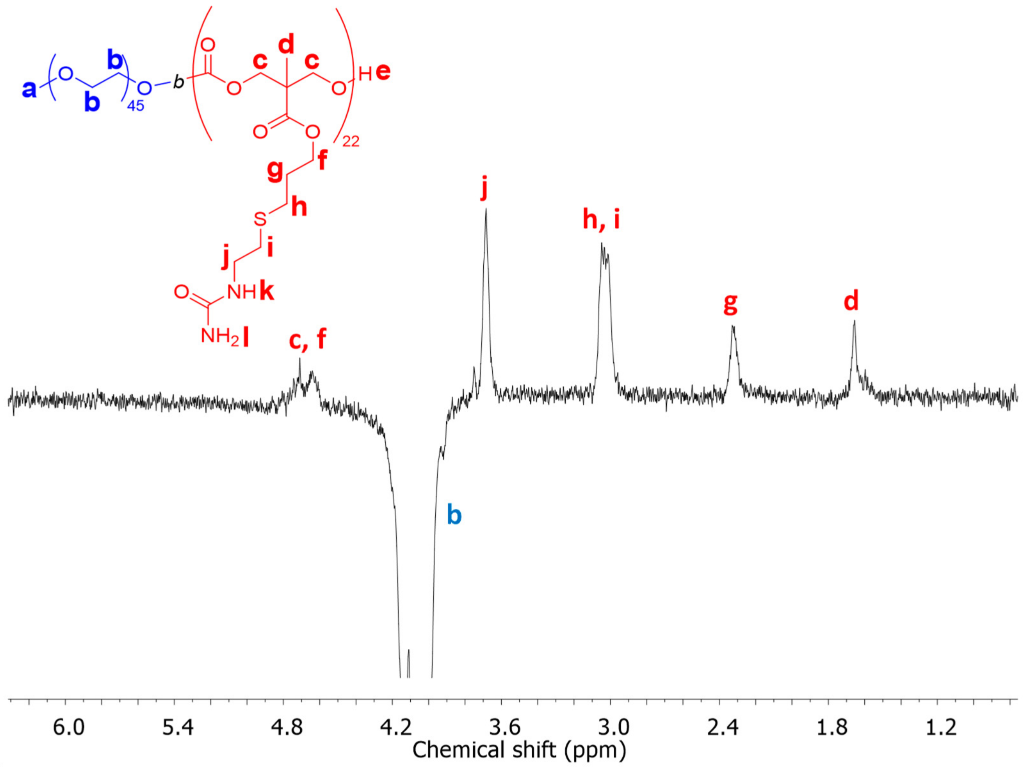

(b)

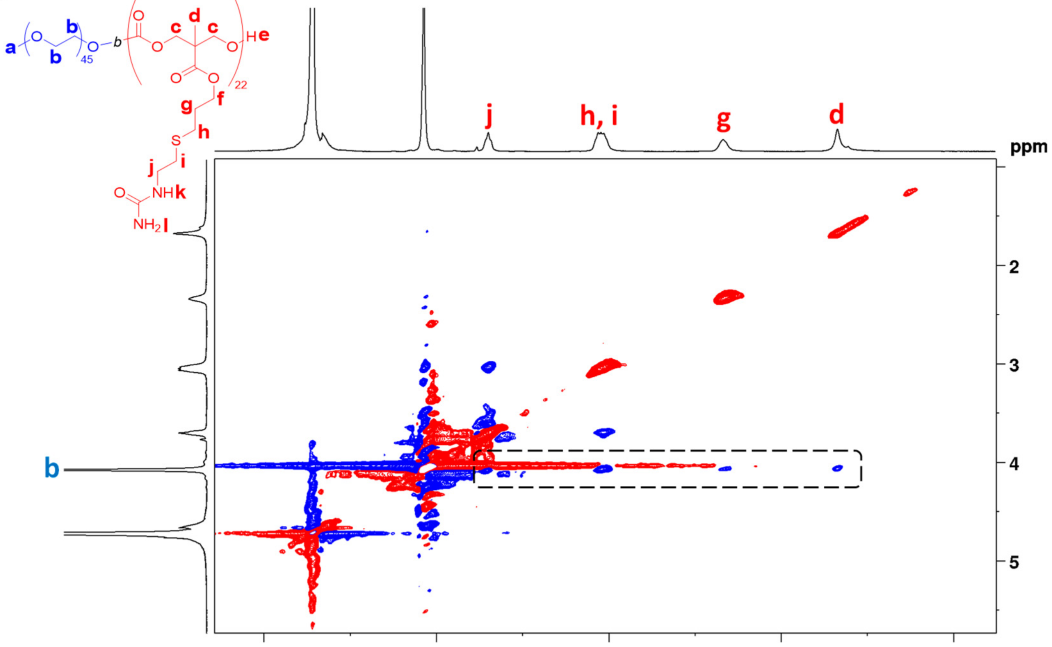

Figure S31. (a) ^{1}H ROE selective spectra of PEG$_{45}$-*b*-P(MACU)$_{22}$ BC in D_2O at 5.0 mg mL^{-1} irradiating the methylenic protons of PEG (labelled as *b*) at 4.06 ppm. **(b)** ^{1}H-^{1}H ROESY spectra of PEG$_{45}$-*b*-P(MACU)$_{22}$ BC in D_2O at 5.0 mg mL^{-1}

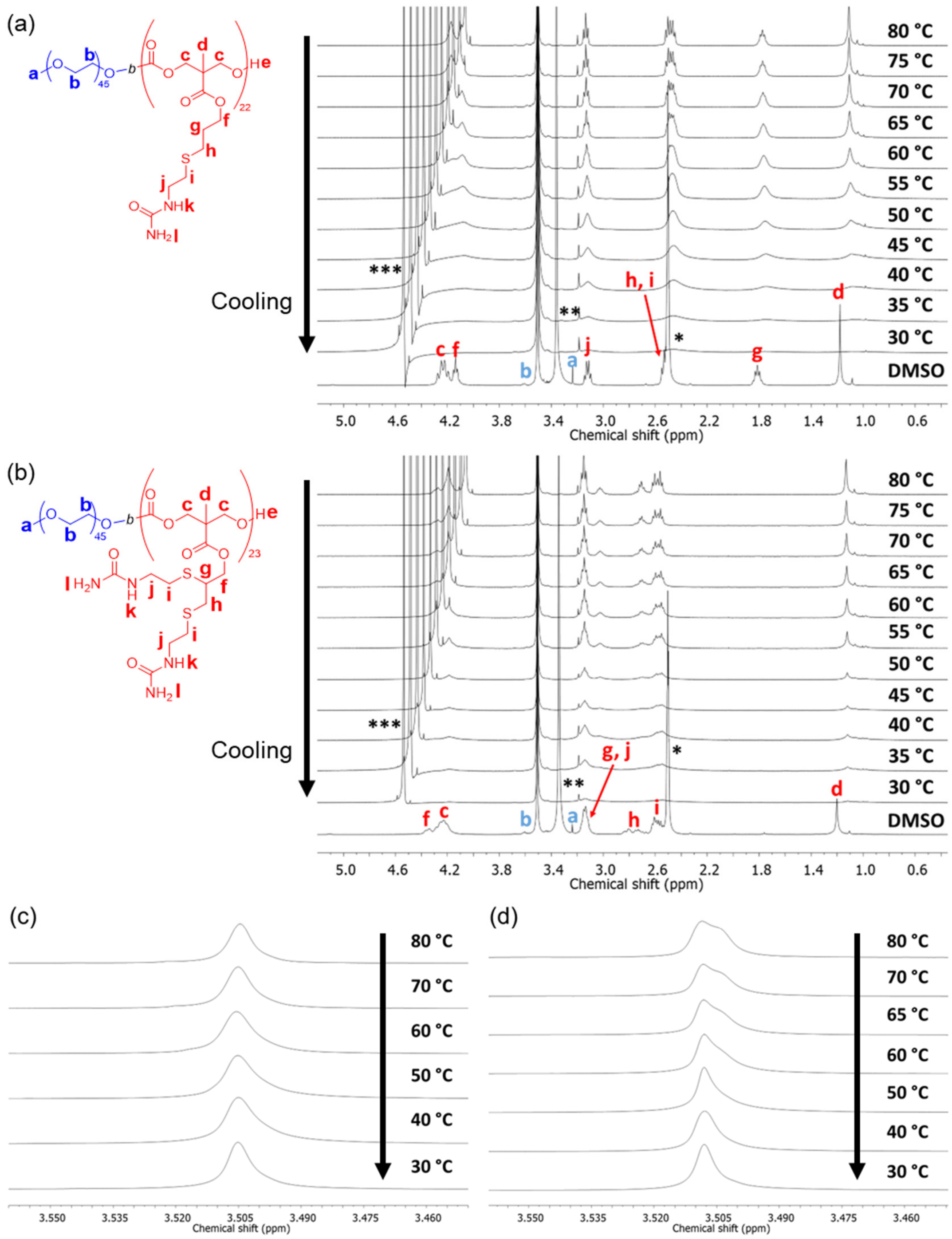

Figure S32. ^{1}H NMR (400 MHz, DMSO-d_6) reference spectrum at 25 °C and temperature-dependent ^{1}H NMR (400 MHz, D_2O) spectra of **(a)** PEG$_{45}$-*b*-P(MACU)$_{22}$ and **(b)** PEG$_{45}$-*b*-P(MPCU)$_{23}$ at 5.0 mg mL^{-1} concentration registered upon cooling, with temperature intervals of 5 °C and equilibrium time of 5 min between each measurement. Partial ^{1}H NMR spectra of **(c)** PEG$_{45}$-*b*-P(MACU)$_{22}$ and **(d)** PEG$_{45}$-*b*-P(MPCU)$_{23}$ depicting the methylenic protons of the PEG block (labelled as *b*) at 3.56-3.45 ppm upon cooling. * Resonances corresponding to protons of DMSO. ** Resonances corresponding to protons of water. *** Resonances corresponding to protons of HDO

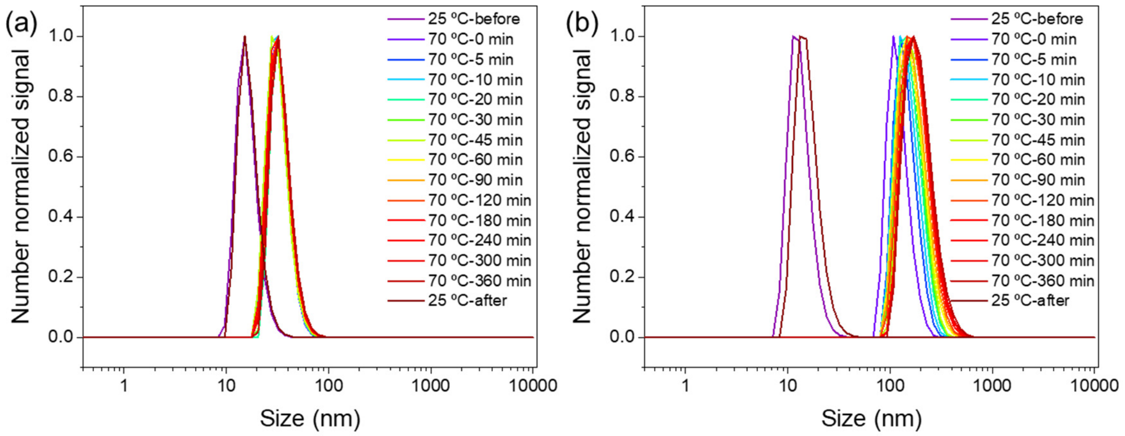

Figure S33. DLS number size distributions of **(a)** PEG45-*b*-P(MACU)22 and **(b)** PEG45-*b*-P(MPCU)23 dispersions (1.0 mg mL^{-1}) quickly heated from 25 to 70 ºC

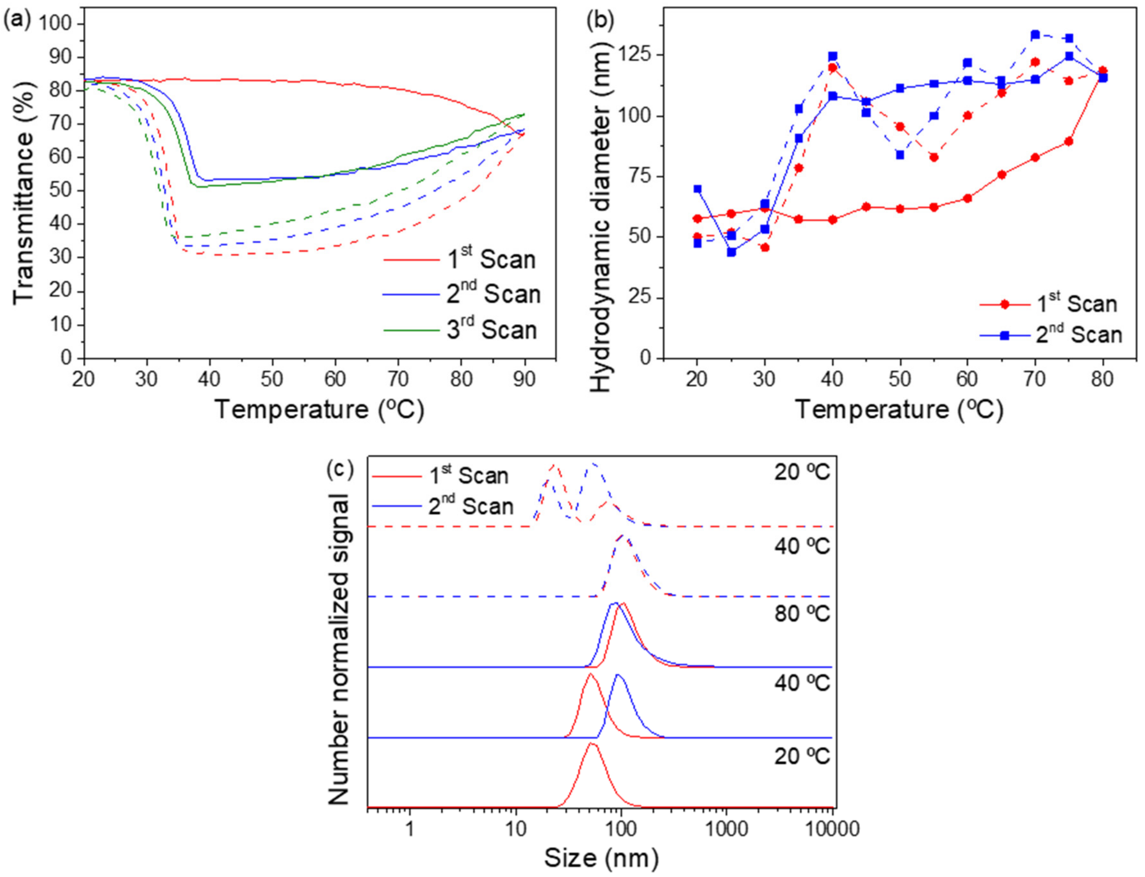

Figure S34. Temperature-dependent **(a)** transmittance curves and **(b)** temperature evolution of number average D_h for PEG45-*b*-P(MPCU)46 dispersions upon heating (solid line) and cooling (dashed line). **(c)** DLS number size distributions at different temperatures of PEG45-*b*-P(MPCU)46 dispersion upon heating (solid line) and cooling (dashed line). Samples were prepared by the co-solvent method at a polymer concentration of 1.0 mg mL^{-1}

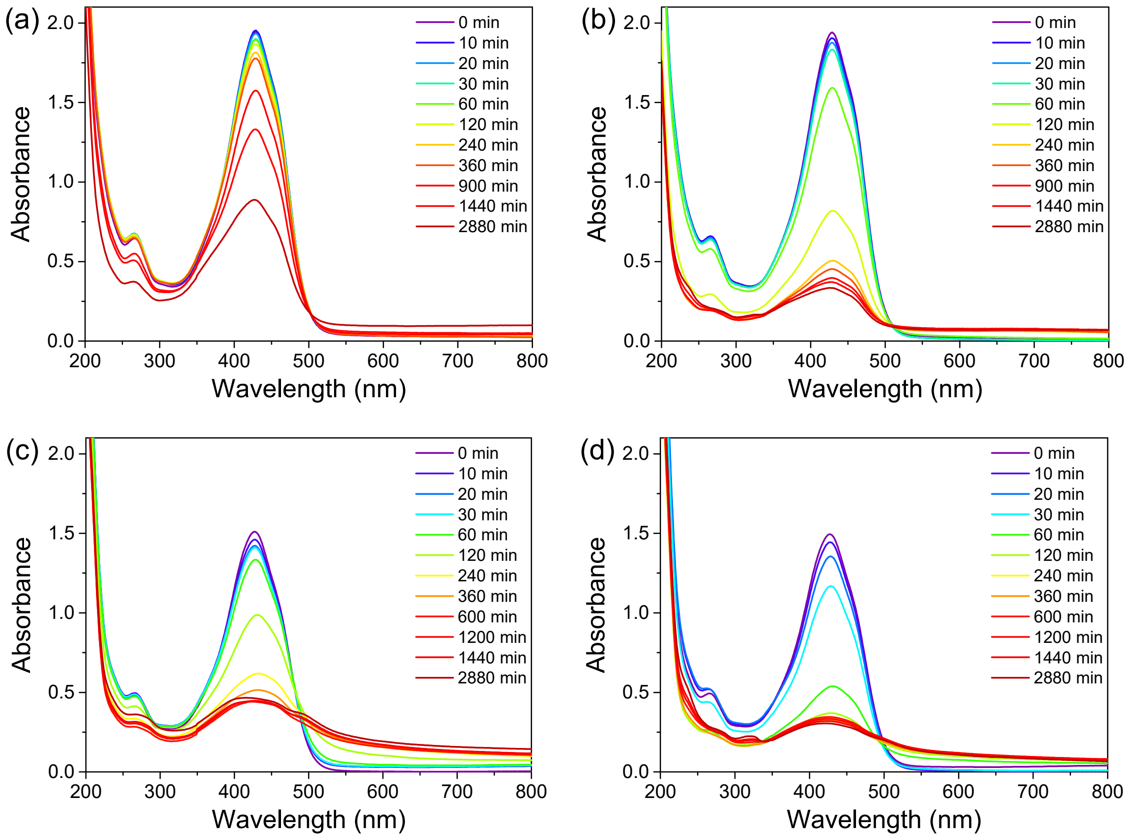

Figure S35. UV-vis spectra of Cur/PEG45-*b*-P(MACU)22 upon heating at **(a)** 25 °C and **(b)** 50 °C and UV-vis spectra of Cur/PEG45-*b*-P(MPCU)23 upon heating at **(c)** 25 °C and **(d)** 50 °C. Samples were prepared by co-solvent method and diluted at 0.1 mg mL^{-1} polymer concentration

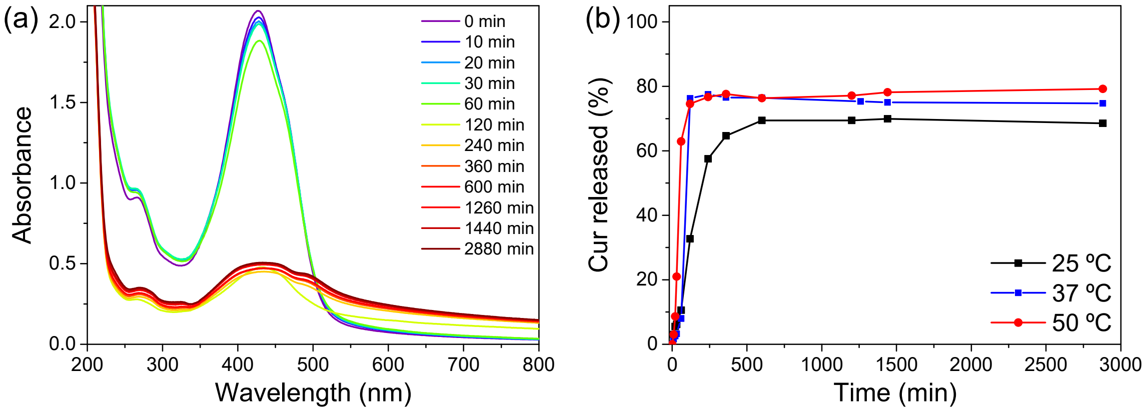

Figure S36. (a) UV-vis spectra of Cur/PEG45-*b*-P(MPCU)22 upon heating at 37 °C. **(b)** Cur release profiles over the time from PEG45-*b*-P(MPCU)23 nanocarriers in water at 0.1 mg mL^{-1} polymer concentration at 25, 37 and 50 °C

References

[1] E. W. P. Tan, J. L. Hedrick, P. L. Arrechea, T. Erdmann, V. Kiyek, S. Lottier, Y. Y. Yang, N. H. Park, *Macromolecules* **2021**, *54*, 1767.
[2] R. C. Pratt, B. G. G. Lohmeijer, D. A. Long, P. N. P. Lundberg, A. P. Dove, H. Li, C. G. Wade, R. M. Waymouth, J. L. Hedrick, *Macromolecules* **2006**, *39*, 7863.
[3] I. Ortiz de Solorzano, L. Uson, A. larrea, M. Miana, V. Sebastian, M. Arruebo, *Int. J. Nanomedicine* **2016**, *11*, 3397.
[4] S. Tempelaar, L. Mespouille, P. Dubois, A. P. Dove, *Macromolecules* **2011**, *44*, 2084.
[5] C. Czysch, T. Dinh, Y. Fröder, L. Bixenmann, P. Komforth, A. Balint, H.-J. Räder, S. Naumann, L. Nuhn, *ACS Polym. Au* **2022**, *2*, 371.
[6] A. Can, E. Altuntas, R. Hoogenboom, U. S. Schubert, *Eur. Polym. J.* **2010**, *46*, 1932.
